# The structure of executive functions in preschool children and chimpanzees

**DOI:** 10.1038/s41598-022-08406-7

**Published:** 2022-04-19

**Authors:** Christoph J. Völter, Eva Reindl, Elisa Felsche, Zeynep Civelek, Andrew Whalen, Zsuzsa Lugosi, Lisa Duncan, Esther Herrmann, Josep Call, Amanda M. Seed

**Affiliations:** 1 School of Psychology and Neuroscience, University of St Andrews, St Andrews, UK; 2 Comparative Cognition, Messerli Research Institute, University of Veterinary Medicine Vienna, Medical University of Vienna, University of Vienna, Vienna, Austria; 3 Department of Anthropology, Durham University, Durham, UK; 4 Department of Comparative Cultural Psychology, Max Planck Institute for Evolutionary Anthropology, Leipzig, Germany; 5 Roslin Institute, University of Edinburgh, Edinburgh, UK; 6 Division of Psychology, University of Stirling, Stirling, UK; 7 School of Medicine, Medical Sciences and Nutrition, University of Aberdeen, Aberdeen, UK; 8 Department of Psychology, University of Portsmouth, Portsmouth, UK

**Keywords:** Human behaviour, Animal behaviour

## Abstract

Executive functions (EF) are a core aspect of cognition. Research with adult humans has produced evidence for unity and diversity in the structure of EF. Studies with preschoolers favour a 1-factor model, in which variation in EF tasks is best explained by a single underlying trait on which all EF tasks load. How EF are structured in nonhuman primates remains unknown. This study starts to fill this gap through a comparative, multi-trait multi-method test battery with preschoolers (N = 185) and chimpanzees (N = 55). The battery aimed at measuring working memory updating, inhibition, and attention shifting with three non-verbal tasks per function. For both species the correlations between tasks were low to moderate and not confined to tasks within the same putative function. Factor analyses produced some evidence for the unity of executive functions in both groups, in that our analyses revealed shared variance. However, we could not conclusively distinguish between 1-, 2- or 3-factor models. We discuss the implications of our findings with respect to the ecological validity of current psychometric research.

Human cognition is unique in the animal kingdom. However, there is intense debate concerning the precise mechanisms that underlie this departure from the cognitive capacities of nonhuman animals^1,2^. In order to identify similarities and differences between human and nonhuman animal cognition, researchers typically use experiments that directly compare performance between two or more species on some target ability. However, the ability under investigation is usually assessed with only a single task. Such studies have established several candidate mechanisms for the ‘small change that makes a big difference’, such as theory of mind, episodic memory, future planning, causal reasoning, inequity aversion, to name just a few. But often, the content validity of a particular task is not established (i.e., whether the task elicits theoretically predicted response profiles), nor is its construct validity (i.e., convergence in performance with other tasks measuring the same ability and divergence in performance on tasks supposed to measure different abilities). This makes it difficult to assess to what extent performance differences are really due to the cognitive ability in question^3^. Nevertheless, differences in test performance between species are readily interpreted as indicative of a difference in the cognitive ability that the test was supposed to measure (e.g., “children are flexible, chimpanzees are conservative”). In order to make progress on the question of what cognitive differences exist between species, more systematic studies are needed that use multi-trait, multi-method test batteries, with several tasks per construct and established construct validity for each tested species^3–7^.

A prominent explanation for human uniqueness focuses on a set of socio-cognitive abilities such as understanding communicative cues, imitation, teaching, theory of mind, and prosociality^8,9^. Herrmann et al.^9^ conducted a systematic study comparing the performance of human children, chimpanzees and orangutans on a test battery assessing physical and social cognition skills (physical: space, quantities, causality; social: communication, theory of mind, social learning). Their findings suggested that 2.5-year-old humans’ abilities already exceed those of nonhuman great apes (hereafter great apes) in the social domain, whereas basic physical cognition skills are on a comparable level (see also^10^). Yet, it was noted that children also exceeded great ape performance in two causality tasks^9^. Therefore, it is possible that humans differ in a broader set of cognitive skills, such as in the ability to understand (social or non-social) unseen causal forces. Alternatively, humans could differ in even more domain-general abilities such as executive functions (EF), an umbrella term describing the set of cognitive capacities allowing for goal-directed behaviour, planning, mental flexibility, and self-monitoring^1,11–15^, that come into play when dealing with hidden, incomplete or conflicting information, and when executing a complex behavioural sequence. The three main cognitive capacities encompassed by the term EF are inhibition (deliberate overcoming of a prepotent response), working memory (WM; involving maintenance, monitoring, updating, and manipulation of short-term memory contents, also in the face of interference), and attentional set shifting (flexibly switching between mental sets or tasks). However, part of the difficulty in diagnosing the source of cognitive differences between human and nonhuman primates is that it is not clear which abilities hang together. Despite being referred to as “domain-general”, we do not know if inhibition, WM, and attention shifting have evolved together, or are rather dissociable skills that might have undergone separable changes in response to selective pressure.

In the human adult literature a large number of studies have used factor analyses to investigate the structure of EF^16^. Factor analysis refers to a statistical method that aims to reduce a number of correlated variables (here: task performances), based on common variance between them, to a smaller number of latent variables, or factors. While no one model has received unequivocal support, there is considerable evidence for both unity and diversity, i.e., while discrete EF dimensions could be identified, tasks across EF also have shared variance. The most commonly accepted model has a nested structure with a common EF factor (all tasks load on this factor) and two nested factors, one specific to attention shifting, the other one specific to WM updating^17^ (but see^18^). According to Friedman and Miyake^17,19^, the common EF factor might reflect the ability to effectively manage and maintain goals and to influence lower-level information processing in line with these goals. The shifting-specific factor can be interpreted as flexibility to switch between representations of goals, tasks, and mental sets whereas the updating-specific factor might refer to the precision of information maintenance and updating. However, it is not clear if this structure is present from early in life or emerges as a result of experience and/or maturation, with factors such as language enriching all aspects of cognitive control in a correlated fashion, leading to the shared variance.

The developmental trajectory of the structure of EF is therefore an important piece of the puzzle. While there are a great number of studies showing developmental improvement in different EF^20^, the structure of EF in young children and their development across the lifespan is less well-studied^16,21^. It has been suggested that the structure of EF changes with development, starting out with a single factor and differentiating across early and middle childhood^16,21^. However, there are fewer studies compared to the adult literature, and these have been criticized for not including measures of attentional set shifting or doing so with only a single task^16,21^.

Compared to the extensive literature on the development and structure of EF in human adults^22,23^, very little is known about the capacities of nonhuman primates. There is suggestive evidence for an increase in EF capacity in the lineage leading to humans. MacLean et al.^24^ have shown differences in the capacity for self-control between nonhuman primates, with a particular increase in ability in the lineage leading to the great apes. Similarly, ManyPrimates et al.^25^ found a marked increase in short-term memory abilities in the lineage leading to the great apes. Studies that directly compared great apes and humans found evidence that this pattern of increasing capacity with increasing brain size continues: young human children outperform great apes in attentional control and inhibition^25–28^. While these studies are a crucial first step, they have only focused on a single construct and a single task at a time, so leave open the question of what mechanisms underpin these changes in capability.

The present study aimed to systematically investigate and compare the structure of EF in great apes and human children. For this, following the human adult cognitive literature, we developed a test battery of nine non-verbal EF tasks, spanning the three main capacities of WM, attentional set shifting, and inhibition (three tasks per function^29^), and administered it to a sample of 3- to 5-year-old children as well as chimpanzees, one of our closest living primate relatives. In creating our test battery, we started by identifying the ability of interest that the tasks within each EF should capture and which response profiles and error patterns they ought to produce (to establish content validity^3^). The goal was to develop tasks which showed the predicted response profile/error patterns, captured individual variation (i.e., did not show floor or ceiling effects), and did not require extensive training periods (chimpanzees) or verbal instructions (children). After having established the content validity of the tasks in pilot phases with both chimpanzee and human samples (see Tables 1 and S1), tasks were combined to form a multi-trait multi-method test battery. Triangulating on each putative trait with multiple tasks differing in surface features aims to address the task impurity problem, i.e., that each task measuring one of the EF components also measures one or more of the other components to some degree^29,30^. The multi-trait aspect aims at establishing construct validity, by providing scope to uncover both convergent and discriminant validity^3,31^. We conceived our study as a parallel investigation of the structure of EF in children and chimpanzees, but did not aim to make direct comparisons between the two species.

**Table 1:** Overview of descriptions and signatures for the nine executive functions tasks.

Function	Task		
	Boxes	Cylinder	Grid
**Inhibition**			
Challenge: Inhibit reaching to visible, inaccessible reward	Forced choice between 2 boxes	Search 24 cylinders, 12 baited	Search 13 doors, 6 (children)/7 (chimpanzees) baited
Dependent variable	Proportion correct (out of 12 possible trials)	Proportion correct (out of first 12 unique searches)	Proportion correct (first 6 unique searches)
Signature: Initial bias for option with visible, inaccessible reward	Children 88% Chimpanzees 85%	Children 94% Chimpanzees 81%	Children 82% Chimpanzees 65%

	Boxes	Shelf	Tray
**Shifting**			
Challenge: Control attention within stimulus set	Compound discrimination with 2 boxes—attend to relevant dimension	Context-dependent discrimination, different box rewarded on each shelf	Compound discrimination—shift away from previously rewarded dimension
Dependent variable	Proportion correct (children: out of max 24 searches; chimpanzees: max 72)	Proportion of possible shifts achieved (out of max 11)	Proportion correct (children: out of max 36; chimpanzees: max 48)
Signature: Error pattern	Children: better performance in Simple Discrimination task, in which only one dimension is present	Shifting errors (out of all errors): Children 76% Chimpanzees 69%	Pilot study with 43 3- to 5-year-old children showed bias towards dimension “boxes” if children had previous experience with tasks in which boxes were used as hiding places

	Boxes	Grid	Updating
**Working memory**			
Challenge: Remember location of reward resisting interference from a secondary task	1 reward in 4 boxes; secondary task another reward in an identical array	1 reward in a 4 × 4 grid; secondary task 1 reward in 3 cups	4 rewards in 4 cups; secondary task 4 more rewards in an identical array
Dependent variable	Proportion correct (children: out of 8 searches; chimpanzees: 24)	Mean proximity to correct location (from 12 trials)	Proportion correct (children: 6 searches; chimpanzees 24 searches)
Signature: Mean performance in single task compared to dual task	Children: 1 task 85%, 2 tasks 56% Chimpanzees: 1 task 60%, 2 tasks 38%	Children: 1 task 94%, 2 tasks 77% Chimpanzees: 1 task 63%, 2 tasks 65%	Children: 1 task 88%, 2 tasks 74% Chimpanzees: 1 task 59%, 2 tasks 51%

Data presented in the “signature” rows stem from the current study (for the sample sizes for each task, see Tables 2 and 3), with the exception of the Shifting Tray task, for which we established content validity in a pilot study with 43 3- to 5-year-old children. For most tasks, we also ran pilot studies and these confirmed the signatures (see Table S1). We did not use “proportion correct” dependent variables for the WM Grid and the Shifting Shelf task because the alternative dependent variables (WM Grid: proximity to correct location, standardized to values between 0 and 1 with higher values indicating higher proximity to the correct location; Shifting Shelf: proportion of achieved shifts) yielded more variation between individuals and evidence for above-chance performance in children and chimpanzees (see Supplementary Material).

To our knowledge, this is the first time that the structure of EF has been tested with a 3 (constructs/capacities) × 3 (tasks per construct/capacity) test battery in preschool children and chimpanzees, and the first time that all tasks are non-verbal (Table 1, Fig. 1). The three inhibition tasks required choosing an opaque container over a transparent one with a visible but inaccessible reward. The three WM tasks required subjects to remember the location of a reward over a short period in which they faced interference from a secondary task. Finally, the three shifting tasks required overcoming fixation on a previously relevant stimulus dimension and focusing on another dimension.

**Figure 1:**
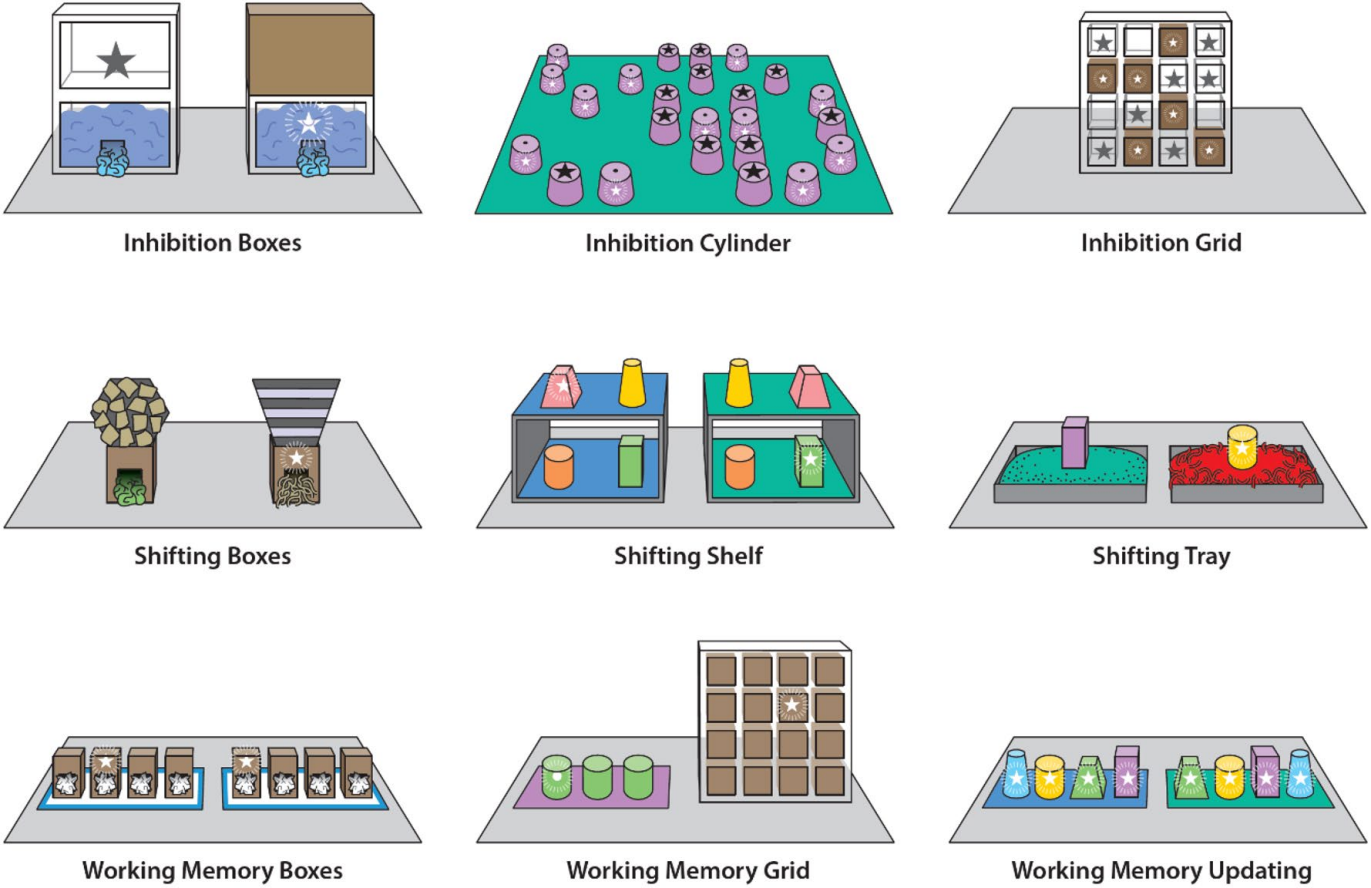
The nine executive functions tasks administered. In the Inhibition tasks, subjects were supposed to choose an opaque container (box, cylinder, or grid cell) with a non-visible reward over a transparent one with a visible but inaccessible reward. In the Shifting Boxes and Shifting Tray tasks, subjects were required to overcome a fixation on a previously relevant stimulus dimension and focus on another dimension. In the Shifting Shelf task, subjects had to switch between two different target cups depending on the context (i.e., the shelf they were choosing from). The WM Boxes and WM Grid tasks required subjects to remember the location of a reward over a short period in which they faced interference from a secondary task. In the WM Updating task, subjects had to remember their previous choices to determine which cups still contained a reward while alternating between two identical sets of cups.

## Results

### Establishing content validity

We first established the content validity of the tasks (see Table 1 for data from the current study and Table S1 from data from the pilot studies). As expected, both children and chimpanzees started the Inhibition tasks with a prepotent bias towards the visible but inaccessible reward item. They performed better in the warm-up trials of the WM tasks in which they did not experience any interference from a distractor task compared to the test trials (except for the chimpanzees in the WM Grid task). In the Shifting Shelf task, both species exhibited a specific type of error (switching mistake) indicative of attention capture by the previously relevant dimension. This error was less present during the warm-up trials, which posed the same choice without the demands on attention shifting. In the Shifting Tray task, pilot work with children demonstrated that if participants had previous experience with a memory task involving the same boxes as hiding places as in the Shifting Tray task, they needed more trials to shift their attention to the dimension “filling material” than when they had no prior experience. Finally, the Shifting Boxes task performance revealed a difference between the initial discrimination without distraction and the compound discrimination with a competing stimulus dimension (though the direction of that difference varied between children and chimpanzees, see “Methods” section).

### Investigating the structure of executive functions in chimpanzees and children

**Figure 2:**
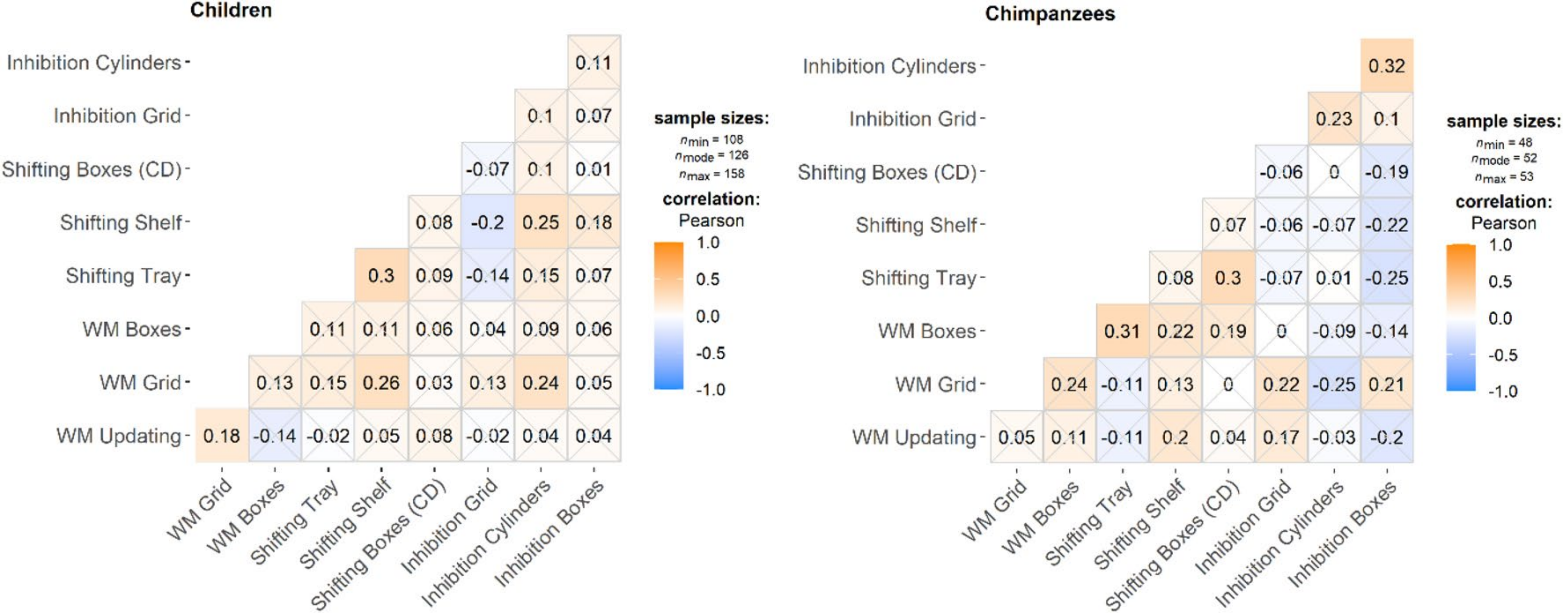
Pearson correlations across all nine tasks for the child sample (left, n = 185) and the chimpanzee sample (right, N = 55; variables centered by site) using pairwise complete observations. Non-significant correlations (*p* > 0.05) are crossed out. Positive correlation parameters are depicted in orange, negative values in blue.

Next, correlations between tasks were examined. For both species, correlations between the dependent variables of the nine tasks were low to moderate and significant correlations were not confined to tasks within the same EF (Fig. 2, Tables S7 and S40).

The finding of correlations not being restricted to occur between tasks within an EF is not surprising, given the “unity” aspect of EF, and fits with previous literature on the structure of EF in humans^16,29,31–36^. For preschoolers, unlike the chimpanzees, most correlations were positive, something that has been referred to in the psychometric literature as the “positive manifold”^37^, describing the phenomenon that scores on cognitive tests are often positively correlated, regardless of the exact nature of the task^38,39^. However, while for our child participants the sample size (n = 185) was sufficiently large to result in stable correlation estimates, the estimates for the chimpanzees (n = 55) probably reflect some larger fluctuations from the true correlations^40^. Descriptive statistics for the nine EF tasks are summarized in Tables 2 and 3 (see also Fig. S23 and S27).

**Table 2:** Descriptive statistics for the nine executive functions tasks for the entire child sample group (N = 190).

Task	Mean	sd	n	Proportion of participants contributing to CFA
Inhibition Boxes	0.74	0.24	126	0.68
Inhibition Cylinder	0.65	0.28	144	0.78
Inhibition Grid	0.54	0.26	155	0.86
Shifting Boxes	0.56	0.18	132	0.71
Shifting Shelf	0.57	0.26	152	0.82
Shifting Tray	0.74	0.13	148	0.80
WM Boxes	0.51	0.18	148	0.80
WM Grid	0.77	0.08	127	0.67
WM Updating	0.74	0.15	184	0.99

**Table 3:** Descriptive statistics for the nine executive functions tasks for the entire chimpanzee sample (N = 55).

Task	Mean	sd	n	Proportion of participants contributing to CFA
Inhibition Boxes	0.16	0.14	53	0.96
Inhibition Cylinder	0.47	0.13	52	0.95
Inhibition Grid	0.47	0.19	54	0.98
Shifting Boxes	0.70	0.12	53	0.96
Shifting Shelf	0.52	0.22	50	0.91
Shifting Tray	0.57	0.12	52	0.95
WM Boxes	0.37	0.12	53	0.96
WM Grid	0.65	0.07	53	0.96
WM Updating	0.51	0.14	53	0.96

### Confirmatory factor analyses

For each EF (Inhibition, Attentional Set Shifting, WM), we first examined whether the tasks within a function actually loaded on a common factor. For both species, we found that for each function, a common, function-specific factor could not describe the data better than the baseline model (which assumed each task to be one factor), suggesting that there was no evidence that the three tasks within each function loaded on a common factor (Tables S8, S9, S39, S40). This was not surprising given the few and low correlations. This finding is not unprecedented in the literature: For example, concerning the measurement of inhibitory control, several studies have shown that even commonly used tasks to assess inhibition yield low correlations and do not load onto a single factor^40–43^. Similarly, comparative studies did not find support for correlations between different inhibition tasks^3,43–46^.

Next, we fitted several models based on Miyake and colleagues’^29^ seminal study on the structure of EF in human adults (Table 4) to explore the structure of performance in our tasks.

**Table 4:** Overview of the fitted CFA models.

Model	Latent factors and loading tasks
9-factor	WM Updating
	WM Boxes
	WM Grid
	Shifting Tray
	Shifting Shelf
	Shifting Boxes
	Inhibition Cylinder
	Inhibition Boxes
	Inhibition Grid
1 factor	Common factor ~ WM Updating + WM Boxes + WM Grid + Shifting Tray + Shifting Shelf + Shifting Boxes + Inhibition Cylinder + Inhibition Boxes + Inhibition Grid
MF2012 (“Miyake & Friedman, 2012”)	WM ~ WM Updating + WM Boxes + WM Grid
	Shifting ~ Shifting Tray + Shifting Shelf + Shifting Boxes
	Common factor ~ WM Updating + WM Boxes + WM Grid + Shifting Tray + Shifting Shelf + Shifting Boxes + Inhibition Cylinder + Inhibition Boxes + Inhibition Grid
3 factors (allowed to correlate)	WM ~ WM Updating + WM Boxes + WM Grid
	Shifting ~ Shifting Tray + Shifting Shelf + Shifting Boxes
	Inhibition ~ Inhibition Cylinder + Inhibition Boxes + Inhibition Grid
3 independent factors	WM ~ WM Updating + WM Boxes + WM Grid
	Shifting ~ Shifting Tray + Shifting Shelf + Shifting Boxes
	Inhibition ~ Inhibition Cylinder + Inhibition Boxes + Inhibition Grid
2factor1	WM + Shifting ~ WM Updating + WM Boxes + WM Grid + Shifting Tray + Shifting Shelf + Shifting Boxes
	Inhibition ~ Inhibition Cylinder + Inhibition Boxes + Inhibition Grid
2factor2	WM + Inhibition ~ WM Updating + WM Boxes + WM Grid + Inhibition Cylinder + Inhibition Boxes + Inhibition Grid
	Shifting ~ Shifting Tray + Shifting Shelf + Shifting Boxes
2factor3	Shifting + Inhibition ~ Shifting Tray + Shifting Shelf + Shifting Boxes + Inhibition Cylinder + Inhibition Boxes + Inhibition Grid
	WM ~ WM Updating + WM Boxes + WM Grid

#### Children

The factor loadings for all Confirmatory Factor Analyses (CFA) models are presented in Table S11 (for run 2 in Table S16). We first compared each model against the 9-factor baseline model (Table S10; run 2: Table S15). Both runs found that the 1-factor, 3-factor, and 2factor3 models fitted the data significantly better than the baseline model. However, there was no significant difference for the MF2012 (“Miyake & Friedman (2012)”), 3 independent factor, 2factor1, and 2factor2 models.

As the 1-factor model has been suggested to fit the structure of EF in young children best^16,21^, we explored this model in more detail. We investigated whether the 1-factor model would fit the data significantly better than the competing models. While the 1-factor model was found to have a better fit than the MF2012 model in run 2, it did not do so in run 1 (Tables S12 and S16), indicating that the 1-factor model could not be reliably distinguished from any competing model. The proportion of variance in performance explained by the shared factor on each of the nine EF tasks was highest for the Shifting Shelf task, followed by Shifting Tray, WM Grid, and Shifting Boxes (Table S13; run 2: Table S18). This finding indicates that the individual variation in the underlying shared factor was most predictive of performance on the Shifting Shelf task, and suggests that other un-modeled factors explain a greater proportion of the variation for the other tasks.

We also compared the variance–covariance matrix of model 1 (Table S14; run 2: Table S19) with the correlations between tasks from Table S7 in order to check whether the 1-factor-model could pick up the correlations between the tasks. We found that for both runs, the model picked up most correlations involving the Shifting Shelf task. However, the correlations between the WM Grid and WM Updating tasks, and the correlations involving the Inhibition Cylinder task were not highlighted by either model. In addition, the covariance between the Shifting Boxes and the Shifting Tray tasks was highlighted.

As the CFA was not able to distinguish between the different competing models, we decided to additionally conduct an Exploratory Factor Analysis (EFA) in order to gain insight into other models which might better explain the data (see below). Given the issues with the WM Updating task found in the EFA (see below), we conducted another two runs of CFAs for which we removed the WM Updating task in order to further check the robustness of our findings (see Supplementary Material). In broad agreement with the main analysis, we found that in both runs the 1-factor, 3-factor, 2factor3, but also the MF2012 models fitted the data better than the 9-factor baseline model. The 1-factor model also fitted the data better than the MF2012 and 3 independent factors model.

In sum, the result of all our CFAs for the child sample was that the 1-factor, 3-factor, and 2factor3 models fitted the data better than a baseline model in which each of the nine tasks was treated as a separate factor. In addition, the 1-factor model fitted the data better than the “unity and diversity” (MF2012) model^17^ in most, but not all, CFAs. While this may hint at a 1-factor structure underlying children’s performance, which would fit the tentative conclusions emerging from the previous literature^16,21^, there was overall no strong evidence that the 1-factor-model outperformed any of the competing models, which again was not surprising given the few and low correlations. As it became clear from the EFA (see below), some of the tasks (WM Updating, WM Boxes, and Inhibition Grid) were loading on independent factors, which probably made it difficult for the CFA to fit the models.

#### Chimpanzees

As for the children, we fitted several models to explore the structure of performance on the EF tasks (see Supplementary Material). We compared each model against the 9-factor baseline model (Table S43). No model fitted the data significantly better than the baseline model (see Table S44 for the factor loadings for all models). However, due to the small sample size, no strong conclusions should be drawn from the CFA, and we focused on the EFA instead.

### Exploratory factor analyses

#### Children

**Figure 3:**
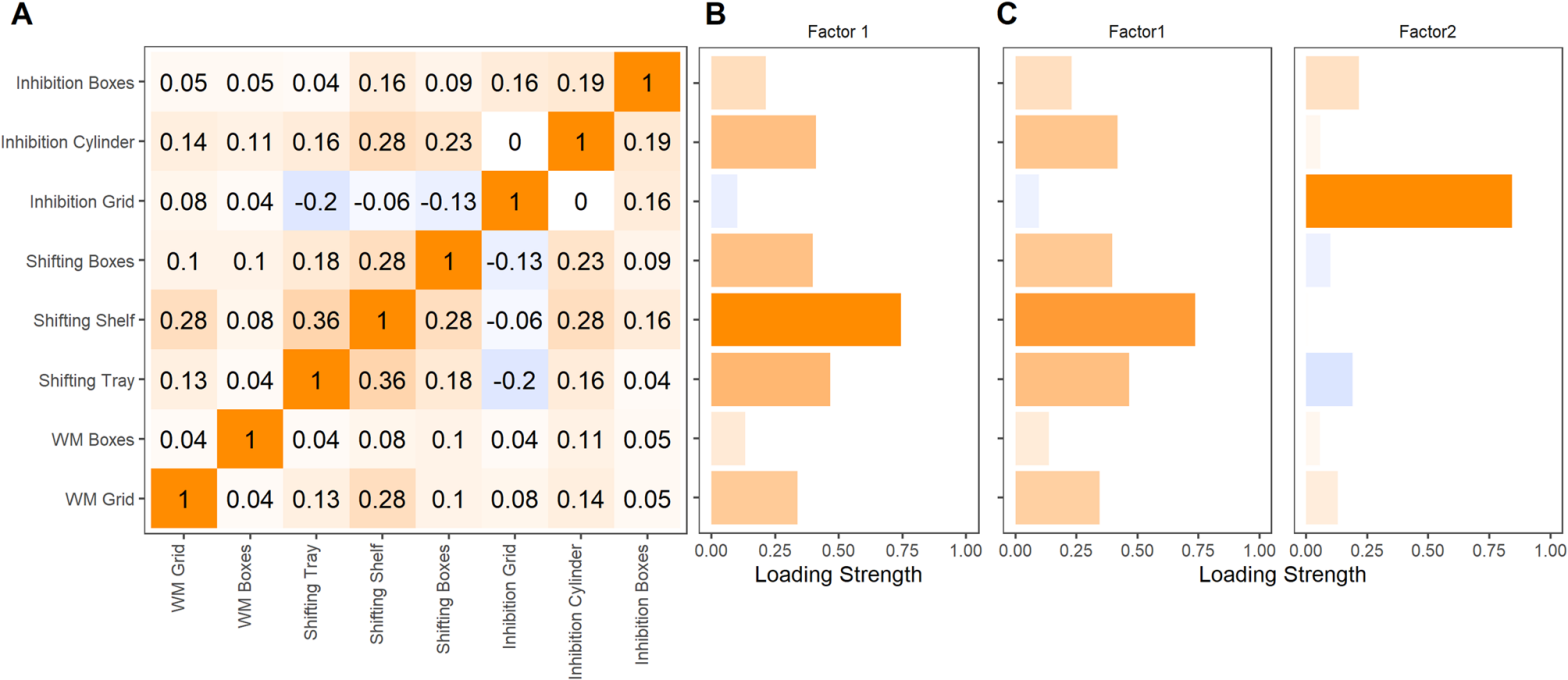
The Pearson correlation matrix (**A**) and the loading strength of all eight tasks (mean centered by site and standardized across sites) that were included in the 1-factor (**B**) exploratory factor analysis (EFA), and for comparison the 2-factor EFA (**C**) for the child sample. Positive values are depicted in orange, negative values in blue. Only participants that completed all eight tasks are included in these analyses (n = 95).

For the EFA, we only included those children who had valid data on all nine EF tasks (n = 95; for the correlation matrix, see Fig. 3A and Table S45). We excluded one task—WM Updating—due to a low Kaiser–Mayer–Olkin measure of sampling adequacy (MSA; see “Methods” section). To determine the number of factors to retain, we investigated how many factors had eigenvalues larger than 1 (with their 90% confidence interval (CI) also being larger than 1^47^), the corresponding scree plot, and conducted a parallel analysis^48,49^. The parallel analysis recommended a 1-factor model (Fig. S31). All tasks loaded on the common factor apart from the WM Boxes and Inhibition Grid tasks, with the strongest loadings found for the Shifting tasks, the WM Grid and Inhibition Cylinder tasks (Fig. 3B; Table S46). The common factor explained 16% of the variance in children’s performance across tasks. The sums of squared loadings was 1.28. Given that a second largest eigenvalue of the correlation matrix was greater than 1 (including its CI; Fig. S31, S32), we also fitted an EFA with two factors for comparison (Fig. 3C, Table S46). Here, the clustering suggested that the Shifting tasks, the WM Grid and Inhibition Cylinder tasks loaded on one factor, with the Inhibition Grid task loading on a second factor. In sum, the EFA also revealed some evidence for the presence of one factor; all the Shifting tasks loaded on this common factor most strongly, followed by the WM Grid and the Inhibition Cylinder tasks. However, the proportion in the variance in performance accounted for was small (16%).

#### Chimpanzees

**Figure 4:**
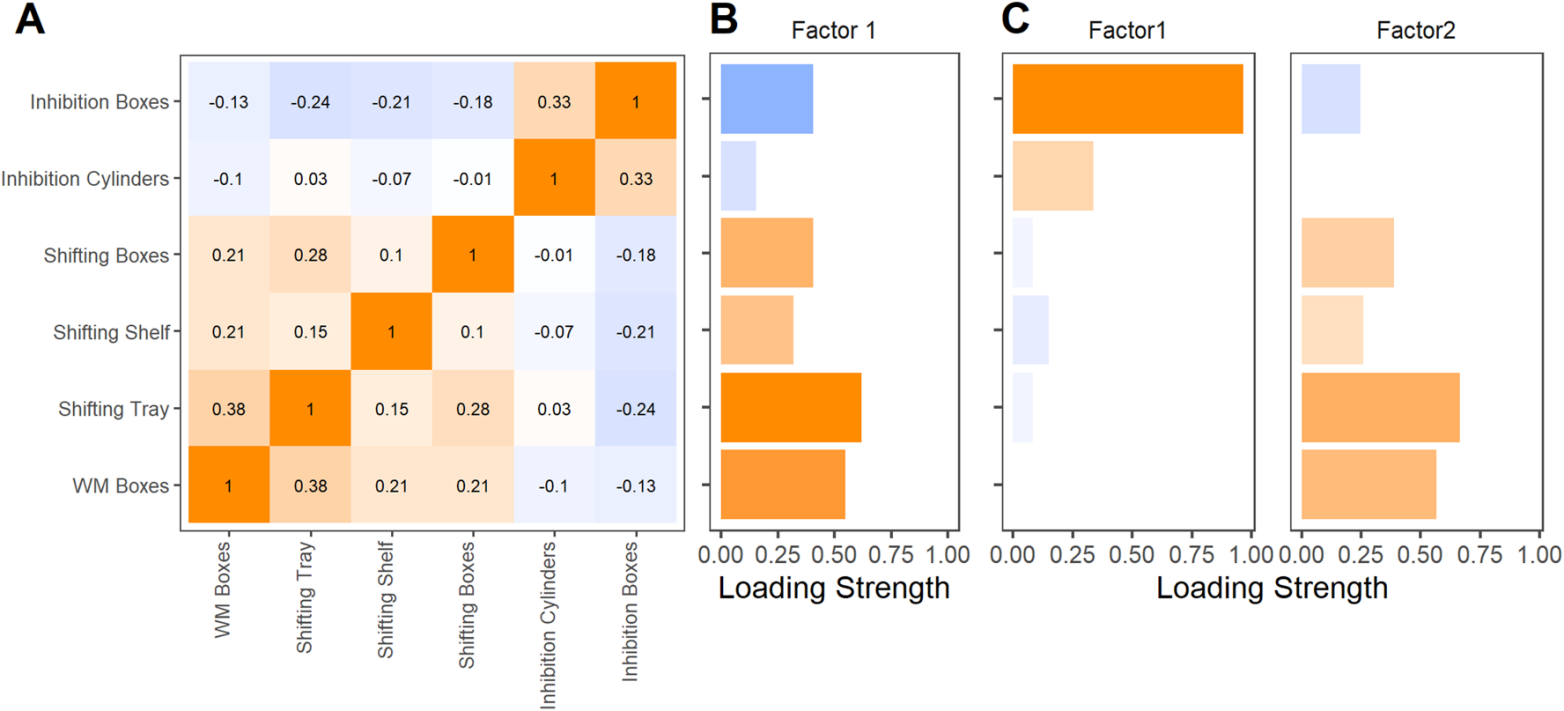
The Pearson correlation matrix (**A**), the loading strength of all eight tasks (mean centered by site and standardized across sites) that were included in the 1-factor (**B**) exploratory factor analysis (EFA), and for comparison the 2-factor EFA (**C**) for the chimpanzee sample. Positive values are depicted in orange, negative values in blue. Only subjects that completed all six tasks are included in these analyses (n = 48).

We only included data of the chimpanzees that had valid data in all nine EF tasks in this analysis (n = 48; for the correlation matrix, see Fig. 4A). We excluded three tasks—Inhibition Grid, WM Grid, and WM Updating—due to low MSA values^50^ and a non-significant Bartlett’s test of sphericity (see “Methods” section). The parallel analysis recommended a one-factor model (Fig. S33). The factor had sums of squared loadings of 1.14 and explained a proportion of 19% of the variance. The clustering of the tasks into factors suggested that all three Shifting tasks and the remaining WM task loaded positively on a common factor, in contrast to the Inhibition tasks. Given that a second largest eigenvalue of the correlation matrix had a 90% CI greater than 1, we also fitted EFAs with two factors for comparison (Fig. 4, S33, S34, Table S47). The two factors had sums of squared loadings of 1.08 and 1.04 and they explained a proportion of the variance of 18% and 17%, respectively. Similar to the 1-factor model, the clustering of the tasks into factors suggested that all three Shifting tasks and the remaining WM task loaded positively on a common factor (factor 2), whereas the Inhibition tasks loaded on another factor (factor 1).

In sum, we found evidence for one latent factor that can be interpreted as a combined WM/Shifting construct. A 2-factor model seemed to be in line with such an interpretation and additionally included an Inhibition factor. However, the variance explained by the factors was relatively low (17% and 18%, respectively). Due to the limited sample size in the study, the 1- and 2-factor models had similar fits to the data, and there was no conclusive evidence that would support a 1-factor model over a 2-factor model.

## Discussion

This study is the first to systematically compare the structure of EF in human preschoolers and chimpanzees. It is also the first to implement a 3 (constructs) × 3 (tasks) test battery for young children and the first to investigate all three main functions of EF (WM, inhibition, attentional set shifting) across two species. Our results overall indicate some unity in the structure of individual differences, consistent with the evidence for adult humans. However, we found no strong evidence for an unequivocal structure in the EF tasks for either preschool children or chimpanzees. Task performance only correlated at low to moderate levels and there was no clear indication of a preference for any one of the models proposed in the literature (see also^16^).

One explanation for the pattern of results is that performance in EF tasks in children and chimpanzees is not well explained by the presence of several dissociable underlying capacities. The lack of a clear structure among EF fits recent theoretical work challenging the notion that EF and their development can be reduced to the workings of clearly separable components^51,52^. These researchers have argued that because EF capacities vary across situations and with experience, they might be better described as skills that emerge as a result of the dynamic interplay of motivations, beliefs, emotions, and prior knowledge rather than context-free abilities that individuals possess. Similarly, at a neural level, the exercise of executive control might best be understood as a dynamic and distributed process involving heavily overlapping networks. In other words, EF are a “moving target” rather than discrete, domain-independent skills. We found no evidence that these constructs are natural kinds that can be identified by tasks raising the same demand in different experimental contexts.

However, the question then arises why research has consistently found a structure involving separable skills of shifting and updating in the EF of human adults. One feature of this research with adults which contrasts with our approach is that the attempt to minimize the task impurity problem has led to the creation of highly standardized tasks for each theoretical skill. The tasks also often overlap in their demands on literacy and numeracy skills. These aspects might facilitate the detection of dissociable, underlying factors (see e.g., the multi-trait mono-method by van der Sluis et al.^53^). Standardizing tasks to remove the influence of other EF, as well as past experience with the environment and previous knowledge, leads to a reduction of ecological validity^51,52^. In contrast, our tasks within each EF were designed to capture the same theoretical trait while differing as much as possible in their general layout and idiosyncratic task demands (e.g., inhibition was measured with a forced-choice task involving boxes, a free search task using a grid apparatus, and a free search task laid out on the floor). The psychometric literature on EF in human adults might have optimized the internal validity of the measures at the expense of their external, ecological validity. It is possible that much of the existing knowledge about the structure of EF is tied to the use of homogeneous task demands and that more research is needed to understand EF in ecologically valid contexts^5,51^.

Conversely, it might be possible that a large part of the variance in our tasks was due to their idiosyncratic features, preventing us from finding a common factor. For example, the Inhibition tasks likely differed in the strength of the elicited prepotent response, and other demands: for instance, in the Inhibition Cylinder task, some children exhibited a spatial search strategy (i.e., lifting cups in an order such as going from left to right) without being selective. Additionally, the contribution of learning abilities might have varied across tasks. In the Inhibition tasks, Shifting Boxes and the Shifting Tray task, the participants had to learn new stimulus–response associations (which conflicted with prior expectations or experience). While our exploratory analysis did not suggest that these tasks loaded on a shared factor, the varying contribution of learning abilities might have blurred the latent variable structure. Relatedly, the use of different dependent variables across tasks might have masked the latent structure. While we used proportion correct for seven out of the nine tasks, we used proportion of achievable switches for the Shifting Shelf task and proximity to the correct location for the WM Grid task. These measures were selected because they provided evidence for substantial inter-individual variation and above-chance performance. The pattern of correlations does not suggest that these two tasks biased the results of the factor analyses. In fact, for the preschool children each of these two tasks was significantly correlated with another task in the same putative executive function. Finding a way to account for variation in learning rates or measures which exclude the learning phase would be desirable for future psychometric research.

It might be the case that dissociable EF constructs indeed exist in children and chimpanzees, but of a different kind to that hypothesized. Perhaps it would be necessary to consider different sub-processes^42,54,55^: Instead of slicing tasks into those that should tap into WM, inhibitory control, and attention shifting, another hypothetical structure might better explain variation in performance in children and chimpanzees, such as along the lines of goal identification, goal maintenance, attentional control, or inhibition versus mental-attentional activation (“M-capacity”)^55–59^. If so, our task demands would have cut across those processes and we would have been unable to identify this latent structure.

One important issue that needs to be addressed is the reliability or repeatability of the measures because it limits the correlation that can be found between them^60,61^. Moderate to high test–retest reliability has been reported for executive function tasks with preschool children^62,63^. In the comparative literature, a recent meta-analysis^64^ found evidence for significant but low to moderate reliability of various tasks including those that aim at measuring learning, memory, physical cognition, and reasoning abilities in different species. However, there was large variation of repeatability across tasks ranging from negative repeatability (larger within-individual than between-individual variability) to highly positive repeatability^64^. Similarly a longitudinal study with great apes found a significant but low to moderate repeatability of different tasks aiming at measuring the same cognitive construct but varying levels of reliability across different tasks^65^.

For the current tasks we found evidence for high test–retest reliability for the WM Updating task with chimpanzees^66^. Additionally, we found significant correlations across sessions for the inhibition tasks with two sessions (chimpanzees only), but these were only moderately sized (see Supplementary Material). These findings suggest that moderate reliability of some measures could indeed impose a limit to establishing construct validity^60,61^. Future research should systematically address this issue in human and nonhuman samples.

Not finding clear evidence for a structure in the EF could also be the result of insufficient power of the latent factor analyses. Firstly, sample sizes were low not only for the chimpanzees, for which sample size usually is an issue^67^, but also for the children, for which testing had to be stopped due to the SARS-CoV-2 pandemic and for which there were also a number of missing data points (of the 185 children entered into the CFA, only 95 (51%) had valid data on all nine EF tasks). It has been suggested that for correlational work sample sizes should approach 250^40^ and for complex latent factor analyses should be even higher^68^. In addition, even though most of the missing data were “missing at random” (MAR) (i.e., because we had to stop testing or due to experimenter error), we cannot rule out that a smaller part of the missing data was not random and might have been related to EF abilities. If so, this would have reduced power as our CFAs treated missing data as MAR or may have created spurious correlations between unconnected tasks. However, if a theoretical clustering of EF in early childhood into a 1- or 2-factor model is indeed a meaningful phenomenon, as has been suggested in recent years^21^, then such clustering should be expected to be also found in our study, despite its limitations, given the fact that we had established the content validity of the tasks, included all three EF components into our test battery, and used three indicators for each component.

For future research, gathering larger sample sizes will be important, as it is particularly desirable for the psychometric approach. In addition, in order to make species comparisons, comparative developmental studies are needed in which human children/adults can be compared to another species’ sub-adults/adults. The current study only consisted of a sample of human children, whose EF are still developing, and a sample of adult chimpanzees. This warrants presenting the results on both species in parallel, but no direct comparisons. Thus, larger and broader sample sizes will be needed to properly tease apart phylogenetic and ontogenetic differences in cognition.

Larger sample sizes, particularly for chimpanzees (and even more so for comparative developmental studies), can likely only be obtained by collaboratively pooling data from different sites^67^. This raises the complication, but also the opportunity, of detecting concurrent population and individual differences. In the current project, we found evidence for such population differences: children from urban areas performed significantly better than those from rural areas in two out of the nine tasks (WM Boxes and Inhibition Boxes task; Fig. S22). The chimpanzees in one sanctuary (Ngamba Island) performed significantly better than those from the other one (Sweetwaters Chimpanzee Sanctuary) in three out of nine tasks (WM Boxes, Inhibition Cylinders and Inhibition Grid task), whereas the opposite was true for one Inhibition task (Inhibition Boxes task; Fig. S26). Other studies looking at short-term memory, learning, and problem-solving abilities also found differences between sanctuary-living and zoo-housed chimpanzees^25,69^. Documenting these population differences might allow over time to link performance variability across populations to external variables including differences in housing and rearing conditions.

In conclusion, we presented the first comparative multi-trait multi-method test battery for the psychometric assessment of EF. The results provide evidence for shared variance underpinning these disparate skills, but no evidence for an unequivocal structure of EF in preschool children or chimpanzees. In line with previous research, these results suggest that the unity and diversity model of EF might play a limited role for explaining individual differences in young children and chimpanzees, particularly when using tasks deviating from the few highly standardized tasks typically administered in the psychometric literature with human adults. However, the conclusions that can be drawn from this study are limited by the small sample size. Nevertheless, the current study introduces a number of new tasks amenable to comparative study, and provides a principled approach for the psychometric assessment of executive functions in nonhuman animals. Future research could build on the new methods described here to shed more light on the structure of individual differences in EF. Documenting the psychometric properties of novel tasks including their between-subject variability and test–retest reliability will be important in this endeavour^60^. Based on the performance levels, error patterns, reliability, and between-subject variability, the Shifting Shelf, Inhibition Cylinder, WM Updating and WM Grid tasks appear to be the most promising candidates for future comparative investigations of individual differences in EF. Finally, large-scale research collaborations will allow us in the future to increase the power and robustness of such analyses^25,67^. This approach will eventually elucidate whether there is indeed no meaningful diversity in the structure of EF in young children and chimpanzees, and ultimately contribute to our understanding of how cognition evolves.

## Methods

### Participants

Ethical approval for all experimental procedures was granted by the University of St Andrews, UK, School of Psychology and Neuroscience Ethical Review Committee prior to the study (child study: approved on 24/04/2018; chimpanzee study: approved on 13/09/2018). All methods were carried out in accordance with the relevant guidelines and regulations. Informed written consent was obtained from the parents/guardians of the children prior to the study. For the chimpanzees, research permits by the local authorities were obtained prior to the study. The study is reported in accordance with ARRIVE guidelines^70^.

#### Children

We tested 190 children (103 girls, 87 boys) in 18 nurseries and schools recruited in small towns (n = 114; “rural area”) and a medium-sized city (n = 76, “urban area”) in Scotland, UK, between June 2019 and March 2020. Of those, 185 children contributed data to the CFA (i.e., they completed at least one EF task). Our target sample size was 200, however testing had to stop early due to the SARS-CoV-2 pandemic. For the EFA, we could only include those children who had valid data on all nine tasks (n = 95). We had consent from another 10 children, but did not test them because they were too shy (2x), below the age of 3 (2x), did not want to take part (1x), stopped the first game and did not want to play on any of the following days (1x), or could not start participation as testing had to stop due to the SARS-CoV-2 pandemic (4x).

We calculated each child’s age in months when they were at the midpoint of their testing duration. The 190 tested children ranged from 3 years 0 months to 6 years 0 months (M = 49.65 months, sd = 7.03 months). Only regarding those 185 children who contributed data to the CFA, mean age was 49.50 months (sd = 6.99, range 3 years 0 months to 6 years 0 months). The sample was predominantly White and highly educated. The ethnic background was given for 165 of the 190 tested children (87%). Of these 165 children, most parents self-identified their children as “White British” (77%), “White” or “Caucasian” (6, 4%), or “European White” (5, 3%). Information on the highest educational degree obtained in the household was provided by 161 parents (85%). Of these, most parents (71%) had a university degree.

#### Chimpanzees

We tested 55 chimpanzees in two sanctuaries (Sweetwaters Chimpanzee Sanctuary, Ol Pejeta Conservancy, Kenya: n = 30; Ngamba Island Chimpanzee Sanctuary, Uganda: n = 25; age range: 5–35 years; median age: 20 years; 30 females; 25 males). We collected the data between October and December 2018 (Sweetwaters) and between October and December 2019 (Ngamba Island). The sample size was determined by the number of available chimpanzees and the available testing times. All chimpanzees were included in the CFA, 48 chimpanzees completed all nine tasks and were included in the EFA. More information about sample characteristics and the recruitment process can be found in the Supplementary Methods. Details about participant numbers and dropouts for each individual task can be found in the Supplementary Results.

### Materials

Information on the materials used for the nine tasks, separated by species, can be found in the Supplementary Material.

### Design and procedure

Nine EF tasks were administered (three tasks per EF; Fig. 1, Tables 1, S3–S5). The common feature of the Inhibition tasks was that participants had to learn to inhibit reaching towards an attractive, but inaccessible reward and instead reach towards an unattractive option which revealed an accessible reward. We used three different versions of this task: the Inhibition Boxes task as a forced-choice task involving boxes, the Inhibition Cylinder task as a free search task with cylinders laid out on the floor, and the Inhibition Grid task as a free search task involving a grid apparatus with flap doors.

The common feature of the Attentional Set Shifting tasks was that participants had to overcome fixation on an item or dimension and shift their attention to another item or dimension. In the Shifting Boxes task, participants first received training in different phases of a forced-choice discrimination learning task involving a variety of boxes serving to focus participants’ attention on one of two dimensions (either box shape or filling material). In the critical phase, participants had to switch their attention away from the more salient dimension towards a less salient one. In the Shifting Shelf task, participants were presented with two identical shelves holding four boxes each. In order to choose the correct box on each shelf participants needed to shift their attention between boxes based on the location of the shelf they were currently presented with. The Shifting Tray task was a forced-choice discrimination learning task in which participants were presented with trays filled with different substrates and different cups on top. Participants had to learn that one of the substrates was predictive of a reward. The Shifting Tray task was placed after a series of tasks in which participants gained extensive experience with tasks involving boxes as containers for rewards. A pilot study (unpublished) with 43 3- to 5-year-old children showed that experience with games involving boxes and cups across 3 days was sufficient to establish a bias in participants towards orienting their attention to the dimension box (children with such an experience needed fewer trials to reach the learning criterion than children who had no such experience). Thus, the Shifting Tray task required participants to shift their attention away from the boxes involved in the task to the unusual dimension filling material.

The common feature of the WM tasks was that participants had to remember and/or update information on a task while resisting interference from a distractor task. In the WM Boxes task, a reward was hidden in a box that was identical to another three boxes on a platform. Participants had to remember where the reward was hidden, while during the retention time another reward was hidden on a second, identical platform. Participants had to remember the location of the first reward while resisting interference from the memory trace of the location of the second reward on the second, identical platform. In the WM Grid task, participants had to remember the location of a reward in a 4 × 4 grid. During the retention interval, participants experienced interference from a distractor game (also a visuo-spatial memory game) in which they had to track the location of a reward in a short object permanence (transposition) task. In the WM Updating task, participants were presented with two identical sets of boxes on top of two identical, adjacent platforms in which rewards were hidden. Participants alternated between both platforms and could choose one box at a time. Participants had to remember and update information on which boxes they had already searched on which platform. The boxes were identical on both platforms to create an interference effect on participants’ memory.

The order and length of the EF tasks is presented in Tables S3 and S4. The order of the tasks was the same for each participant and determined in a way that no two tasks of the same EF were administered in consecutive sessions. The tasks were distributed over 12 (children) and 18 (chimpanzees) testing days (not necessarily consecutive). Participants were tested individually and only one EF task (or a part of it) was administered per day. Further information on the procedure can be found in the Supplementary Methods.

### Scoring

#### Dependent variables

We coded a number of variables to describe performance in each task (see Supplementary Methods). We extracted one main dependent variable for each task which was entered into the factor analyses (Tables 1, S5). We selected those variables as dependent variables which showed large individual variation. For the Inhibition tasks, we included the proportion of correct searches (i.e., choosing the hidden but accessible reward). For the chimpanzees, we chose performance within the first session given that this session performance provided evidence for a prepotent response and that we observed a side bias in their second session of the Inhibition Boxes task. Focusing on the first-session performance also increased the comparability to children who completed only one session in the Inhibition Boxes and Inhibition Cylinder task.

For the Shifting Boxes task, we used the proportion of correct searches in the compound discrimination (CD) phase (as a measure of susceptibility to interference from another stimulus dimension) given that the Extradimensional Shift (EDS) phase resulted in chance-level performance for the chimpanzees and that only very few children advanced to the EDS phase (and those who did made only few mistakes). The dimension predictive of the reward in the CD phase differed for chimpanzees (filling material) and children (box shape) as previous, unpublished, work had shown that when children learned to focus their attention on the filling material in the CD phase, the EDS phase revealed floor effects. Therefore, it was decided to reverse the rewarding dimension for the children as initially it was planned to use performance in the EDS as a dependent variable. In the Shifting Shelf task, we used the proportion of achieved platform switches out of all possible switches. For the Shifting Tray task, we used proportion correct in session 2 for the chimpanzees, as performance in session 1 was at chance level (children completed only one session).

In the WM tasks, all dependent variables captured the performance in the presence of a secondary task. For the WM Boxes and WM Updating tasks, we included the proportion of correct choices on both platforms. For the WM Grid task, we chose the average proximity of the first choice to the baited compartment (standardized to a range between 0 and 1).

### Interrater reliability

For the child sample, 19–33% of the sessions for each task were coded by one of four second coders. This revealed that coding and data entry for Inhibition Cylinder, Inhibition Grid, and Shifting Boxes tasks had been rather error-prone, and so 100% of the data for these tasks were coded by a second coder. Mismatches between the two sets of codings were checked again and resolved, resulting in perfect agreement. For the remaining tasks, perfect agreement was found for WM Updating, Shifting Tray, and Shifting Shelf. For WM Boxes, coding mistakes were found in 3% of the datapoints, in WM Grid 19% and in Inhibition Boxes 4%; all of them were double-checked and corrected, resulting in perfect agreement. For the chimpanzee sample, a second coder scored 20% of all sessions of all nine tasks to assess interobserver reliability, which was good for all tasks (Table S6).

### Establishing content validity

Validity of the tasks in both species was checked by investigating the presence of predicted signature limits (Table S1). For the Inhibition tasks, we checked whether participants exhibited a prepotent response bias at the beginning of testing. In the Inhibition Boxes task, we found a significant preference for the transparent box in the first trial for both species, indicating a strong initial bias for choosing the box with the visible reward: 111 out of 126 tested children (88%) chose the transparent box in trial 1 (binomial test: *p* < 0.001; Fig. S24) as did 45 of the 53 chimpanzees (85%; *p* < 0.001; Fig. S28). In the Inhibition Cylinder task, we expected the majority of participants to pick the cylinder with the attractive reward on display initially (children: warm-up trial; chimpanzees: first test trial). Of the 130 children for which the warm-up was recorded (not recorded for 14 children), 122 (94%) chose the cylinder showing the attractive sticker first and so showed a significant preference for this type of cylinder (binomial test: *p* < 0.001; Fig. S25). Likewise, the chimpanzees (who did not receive a warm-up) exhibited a significant preference for a transparent cylinder in their first choice (42 of the 52 of the chimpanzees chose a transparent cylinder, 81%; binomial test: *p* < 0.001; Fig. S29). In the Inhibition Grid task, both species showed a significant preference for a transparent door in the first choice: 127 of 155 children (82%) touched one of the transparent doors first (binomial test, *p* < 0.001) as did 35 of the 54 chimpanzees (65%; *p* = 0.006; Fig. S30).

In the Shifting Boxes task, we investigated how well children focused their attention on the dimension shape when there was no interference from the second dimension filling material. The latter dimension was more salient as it was closer to the reward and directly handled by the children. For this, we investigated performance in the Simple Discrimination (SD) phase in which the filling material was identical across the boxes: Children’s (n = 141) mean proportion of correct trials was 0.74 (sd = 0.15, range 0.33–1; significantly above chance level (0.5; t(140) = 18.65, *p* < 0.001)) and thus higher than in the CD phase (n = 132; 0.56, sd = 0.18, range 0.21–1; significantly above chance level (t(131) = 3.58, *p* < 0.001)). In order to investigate whether performance in these two phases differed significantly, we also examined a subsample of children who had valid data on both phases (*n* = 130). Children’s mean proportion in the SD phase (0.74, sd = 0.15, range 0.33–1) was significantly higher than in the CD phase (0.56, sd = 0.18, range 0.21–1), two-sided, paired-samples t-test t(129) =  − 9.25, *p* < 0.001.

The learning criterion was reached by 111 children (79%), in contrast to 36 of the 132 children (27%) in the CD phase. In the SD phase, the 111 children who reached the criterion needed on average 10.95 trials (sd = 4.39, range 6–24) to do so, whereas the 32 children who reached criterion in the CD phase needed on average 13.31 trials (sd = 6.51, range 6–24). In contrast, the chimpanzees in the Shifting Boxes task learnt to focus on their relevant dimension filling material increasingly faster across test phases (mean trials to criterion ± sd, range: SD: 28.70 ± 16.11, 7–66; CD: 15.00 ± 6.88, 6–42; Intradimensional Shift (IDS): 8.47 ± 3.23, 6–18), consistent with the notion that their attention was increasingly fixated on the filling material. The chimpanzees performed significantly better in CD than in SD (t(52) =  − 3.45, *p* = 0.001). In all of these phases, they performed significantly above chance (SD: mean correct ± sd: 0.63 ± 0.11; t(52) = 8.31, *p* < 0.001; CD: 0.70 ± 0.12; t(52) = 11.87, *p* < 0.001; ID: 0.88 ± 0.09; t(52) = 29.69, *p* < 0.001). So while adding another stimulus dimension in the CD phase made the task more difficult for the children this was not the case for the chimpanzees possibly due to an increasing fixation on the relevant stimulus dimension. This pattern is consistent with findings from a previous study using the ID/ED task with chimpanzees and preschool children^71^.

In the Shifting Shelf task, we examined the presence of a specific error type in the test phase, namely switching mistakes. These were defined as participants choosing the cup that was predictive of the reward on the other, not the currently presented, shelf. It was assumed that if mistakes were made in the test phase, the majority of participants would commit a switching mistake (choosing the box that was just being rewarded on the other platform) instead of random errors (i.e., choosing a box that had never been rewarded). Indeed, we found that for both species, the majority of mistakes in the test phase were switching mistakes (children: mean proportion ± sd: 0.76 ± 0.19, range 0.21–1; chimpanzees: 0.69 ± 0.23, 0.07–1). We also compared the type of mistakes between the test and the training phases in which there was no conflict between stimuli when participants switched shelves. For this, we examined whether the proportion of trials in which any of the two cups on the wrong level was chosen was higher in the test phase compared to the training phases in which the distractor cups were present (training 2). This was the case for both species: for children, the mean proportion of trials in which a cup on the wrong level was chosen out of all incorrect trials was higher in the test phase (mean proportion ± sd: 0.90 ± 0.11, range 0.5–1) than in both training 2a (0.79 ± 0.26, range 0–1) and in training 2b (0.49 ± 0.40 (range 0–1). In order to investigate whether these differences were statistically significant, we ran two-sided, paired samples t-tests on two subsamples of children: those with datapoints for both training 2a and the test phase (*n* = 141), and those with datapoints for training 2b and the test phase (*n* = 99). The mean proportion of trials in which a cup on the wrong level was chosen out of all incorrect trials was significantly higher in the test phase (mean proportion ± sd: 0.89 ± 0.11, range 0.5–1) than in training 2a (0.80 ± 0.26, range 0–1), t(140) =  − 4.04, *p* < 0.001, as well as in training 2b (0.49 ± 0.41, range 0–1), t(98) =  − 9.17, *p* < 0.001. For the chimpanzees, the proportion of trials in which a cup on the wrong level was chosen out of all incorrect trials was also significantly higher in the test phase (chimpanzees: 0.78 ± 0.20, 0.13–1) compared to the training phases (chimpanzees: 0.48 ± 0.31, range 0–1), t(46) =  − 6.69, *p* < 0.001.

For the Shifting Tray task, an unpublished pilot study with 43 3- to 5-year-old children showed that experience with games involving boxes across three testing days established a bias in participants towards orienting their attention to the dimension box, resulting in children needing significantly more trials to reach the learning criterion compared to children who received the Shifting Tray task without prior experience.

In the WM tasks, we confirmed that participants’ performance was better in the absence of the distractor task. In the WM Boxes task, both species’ proportion of correct trials in the warm-up was significantly higher than in the test phase: Children’s mean proportion of correct trials in the warm-up was 0.85 (sd = 0.24, range 0–1), which was significantly above chance level (chance: 0.25; t(147) = 30.92, *p* < 0.001) and significantly greater than the proportion of correct trials on platform 1 in the test (mean proportion ± sd: 0.56 ± 0.25, range 0–1), t(147) =  − 10.61, *p* < 0.001, which was also significantly above chance level (chance: 0.25; t(147) = 15.02, *p* < 0.001). Likewise, chimpanzees’ mean proportion correct in the warm-up was 0.60 (sd: 0.30, range 0–1), which was significantly better than chance (t(52) = 8.59, *p* < 0.001) and better than their performance on platform 1 in the test (mean proportion ± sd: 0.38 ± 0.15, range: 0.13–0.75, t(53) =  − 4.92, *p* < 0.001), which was still significantly above chance (t(52) = 6.56, *p* < 0.001).

In the WM Grid task, children’s mean proximity to the reward in the warm-up trials was almost perfect: 0.94 (sd = 0.09, range 0.56–1; significantly above chance level (0.46; based on Monte-Carlo simulations assuming random sampling), t(126) = 57.547, *p* < 0.001) and significantly better than performancein the test trials (0.77, sd = 0.08, range 0.55–0.95; significantly above chance level (0.46), t(126) = 40.391, *p* < 0.001), t(126) =  − 18.68, *p* < 0.001. For the chimpanzees, performance in the warm-up trials was similar to the test trials. In both phases, however, the chimpanzees chose doors in their first attempt that were closer to the reward than expected by chance (0.46; warm-up: mean proximity ± sd: 0.63 ± 0.16; t(54) = 7.82, *p* < 0.001; test phase: 0.65 ± 0.07; t(53) = 19.96, *p* < 0.001). The similar performance levels in the warm-up and test phase (t(53) = 1.00, *p* = 0.321) might be explained by the increased task experience in the test trials (compared to the warm-up), which might have compensated for the additional cognitive load introduced by the secondary task. The results suggest that introducing an interference/secondary task in the test phase has differential impact on chimpanzees and preschoolers.

For the WM Updating task, we investigated performance in the one-platform version of the task (children: Scrambled box task with eight boxes on one platform; chimpanzees: training without scrambling and four or five boxes on one platform), which had served as a warm-up task for the WM Updating task. Children completed two trials in the Scrambled box task; for this analysis, we selected trial 2 (as in trial 1, unfamiliarity with the boxes might have facilitated children’s performance). For further comparability, we deselected round 1 (as this was always a successful round) and selected six searches (round 2–7), to match the number of searches in the Updating task. Mean efficiency in rounds 2–7 in trial 2 of the Scrambled box task was 0.88 (± 0.13, range 0.5–1) and thus higher than in the WM Updating task (0.74 ± 0.15, range 0.33–1). The chimpanzees’ performance in the training session with only one platform did not deviate significantly from the hypothetical chance level (chance level determined by Monte-Carlo simulations assuming random sampling with 4 boxes 0.58 and 5 boxes 0.59; 4-boxes: mean efficiency ± sd: 0.59 ± 0.16; t(52) = 0.32, *p* = 0.747; 5-boxes: 0.62 ± 0.15; t(52) = 1.13, *p* = 0.263). In the test with two platforms, the chimpanzees performed significantly worse than chance (mean efficiency: 0.51 ± 0.14; t(52) = 3.67, *p* < 0.001).

### Confirmatory factor analyses

For both species, we mean centered all dependent variables separately by testing location as we found a difference in children’s performance for the WM Boxes and Inhibition Boxes tasks between the urban and rural areas (Fig. S22) and in chimpanzees’ performance for four tasks (WM Boxes, Inhibition Boxes, Inhibition Grid, and Inhibition Cylinder tasks) between the sanctuaries (see Fig. S26). Then, for each species, data from both locations were combined and standardized as we assumed that despite differences in mean performance between locations, standard deviations would likely not differ.

Sample size for the children was 185. For each task, between 68.1 and 99.5% of the maximum data points were available (Table 2). Most of the time, missing data seemed unlikely to be systematically related to EF abilities (e.g., experimenter error), but it is possible that part of the missing data might not be missing at random (MAR). This may introduce a small bias since our CFA models treated missing data as MAR, resulting in a loss of power making us more conservative in our effect estimates. Sample size for the chimpanzees was 55. For each task, between 91 and 98% of the maximum data points were available (Table 3).

All analyses were carried out in R^72^. The CFAs were carried out using the R package blavaan^73^, a Bayesian version of the R package lavaan^74^. A Bayesian model was used to account for uncertainty due to low sample sizes. For both species, we first examined whether the tasks within each EF (Inhibition, Shifting, WM) actually loaded on a common factor. For this, for each EF, we fitted a model in which the three tasks within the function loaded on a common factor and compared it against a baseline model in which the three tasks loaded on three separate factors.

Next, for both species, we fitted several models based on the cognitive and developmental psychology literature (Table 4). Models were fitted using the bcfa command of the package blavaan^73^, with the parameter sample set to 5000, adapt set to 500, burnin set to 2500, n.chains set to 4, and adapt_delta set to 0.95. All other parameters were set to their default value. The number of samples selected was higher than the default numbers in order to increase robustness to sampling errors. The blavCompare command was used to compare models, using the Widely Applicable Information Criterion (WAIC^75^) to assess whether one model fit the data better than another one. A model was determined to fit the data better than the other if the difference between WAIC scores was greater than twice the standard error. *p* values were calculated by using a Wald test. In order to check on the robustness of the model fit and whether a sufficient number of samples were run, we fitted all models twice (run 1, run 2; for the child sample only). For the chimpanzee sample, none of the models resulted in a better fit than the baseline model; therefore, we did not further investigate the robustness of these models.

We used the default Gamma (1, 0.5) prior, which was deemed a good and conservative fit for the data: For the 1-factor-model, we assumed a priori the common factor to explain between 10 and 70% of the total variance of each task. The Gamma (1, 0.5) prior puts most of the weight between 0 and 1, and the prior probability that the variance is 1 is about a sixth the prior probability that the variance is 0. This was deemed a reasonable fit for the data. For the children, we also ran the models with a more even weighting across the 0–1 range by increasing the value of the second parameter to Gamma (1, 1) and Gamma (1, 2) and found that this did not change the conclusions we drew from the analysis (see Supplementary Results).

#### Children

Most models converged with Rhat values close to 1. The Rhat values give an estimate of how similar the posterior distributions are between the four chains that the models ran, with Rhat values close to 1 indicating that all chains converged to the same solution. However, in both runs, we received warnings for large Rhats for the 1-factor model (largest Rhat was 1.3; in run 2: 1.17), and in run 1 also for the 2factor3 model (largest Rhat was 1.42), indicating convergence issues with these models. We found that the Gelman-Rubin psfr (potential scale reduction factor) was larger than 1.2 for the 1-factor (both runs) and 2factor3 (run 1) models, further indicating convergence issues with these models. However, the fact that we found the WAIC values to be very similar between run 1 and 2 (compare Tables S11 and S16) confirmed that we had used a sufficiently large number of samples for the models to produce robust WAIC values and that we were still able to consistently estimate model fits.

We received a number of other warnings when fitting the models. For all but the 9-factor model there was a warning for the presence of divergent transitions, although the percentages of divergent transitions were small: occurring for between 0.20 and 12.28% of the samples (run 2: between 0.12 and 21.90%). In addition, we received warnings that the Bulk and/or Tail Effective sample sizes were low for the 1-factor, MF2012 (“Miyake & Friedman (2012)”), 3-factor, 3 independent factor, 2factor1, and 2factor3 models (both runs). We received warnings about small effective sample sizes for some parameters for the 1-factor (both runs), MF2012 and 2factor3 (run 1) models. The effective sample sizes give an estimate of how well the samples approximate the posterior distribution. Higher effective sample sizes indicate a more robust posterior. Although we found that some of the models had difficulties estimating some of the parameter values, the consistent WAIC values between runs suggested that overall model fit was still consistently estimated.

#### Chimpanzees

As for the children, we fitted several models to explore the structure of performance on the EF tasks. All models converged with Rhat values close to 1 (all values < 1.01). The Gelman-Rubin psfr (potential scale reduction factor) was low (< = 1.025). We received a number of other warnings when fitting the models. For all but the 9-factor model, there was a warning for the presence of divergent transitions, although the percentages of divergent transitions were small: occurring for between 0.8 and 8.1% of the samples. In addition, we received warnings that the Tail Effective sample sizes were low for the 1-factor model.

### Exploratory factor analyses

Following the inconclusive results of the CFA, we further investigated the number of constructs and their structure with an EFA. Before including items in the EFA, we checked the Kaiser–Mayer–Olkin measure of sampling adequacy (MSA)^76^. The MSA provides an assessment of the suitability of the correlation matrix for a factor analysis. For the chimpanzees, we excluded three tasks—Inhibition Grid, WM Grid, and WM Updating—due to low MSA values^50^ and a non-significant Bartlett’s test of sphericity. The remaining tasks had MSAs ranging between 0.45 and 0.70 (overall: 0.60). Bartlett's test of sphericity for the remaining tasks suggested that the observed correlation matrix was marginally significantly different from an identity matrix (χ^2^ = 24.01, df = 15, *p* = 0.065). For the children, the WM Updating task was excluded due to a low MSA of 0.43. When checking the measure again using the remaining tasks, we found that the MSAs ranged between 0.50 and 0.74 (overall: 0.66). Bartlett's test of sphericity was significant (χ^2^ = 54.223, df = 28, *p* = 0.002).

We determined the number of factors to retain for the EFAs by using the parallel analysis^77^ using the function fa.parallel of R package psych^78^ with maximum likelihood factoring and communalities estimated by the Squared Multiple Correlation. We fitted a maximum-likelihood EFA using the function factanal of the package stats^72^. We used a varimax orthogonal rotation for the interpretation of the factors.

## Supplementary Information

Supplementary Information.

## Data Availability

All data and analysis scripts associated with this study can be found on the following OSF page https://osf.io/mcenz/?view_only=868ef8f463c24607b328deb60b92f860.
