# Supplementary material for: The structure of executive functions in preschool children and chimpanzees
Source: Sci Rep. 2022 Apr 19;12:6456. doi: 10.1038/s41598-022-08406-7 (PMC9017736; doi:10.1038/s41598-022-08406-7)
Supplement: Supplementary file 1 — Supplementary Information. [file 41598_2022_8406_MOESM1_ESM.docx]

**Supplementary material for**

The structure of executive functions in preschool children and chimpanzees

Christoph J. Völter, Eva Reindl, Elisa Felsche, Zeynep Civelek, Andrew Whalen, Zsuzsa Lugosi, Lisa Duncan, Esther Herrmann, Josep Call, & Amanda M. Seed

# **Content [press Ctrl + Click on the heading to jump top section of interest]:**

Supplementary Methods:

- Pilot studies (Table S1)
- Participants
  - Children (Table S2)
  - Chimpanzees
- Materials
  - Children (including Figures S1-S12)
  - Chimpanzees (including Figures S13-S21)
- Design
  - Children (including Table S3)
  - Chimpanzees (including Tables S4, S5)
- Procedure
  - Children
  - Chimpanzees
- Scoring and analysis
  - Children
  - Chimpanzees (including Table S6)

Supplementary Results:

- Individual Tasks
  - Children (including Figures S22-S25)
  - [Chimpanzees](#_Chimpanzees) (including Figures S26, S30)
- Confirmatory Factor Analysis
  - Children (Table S7)
    - Domain-specific models (including Tables S8, S9)
    - Run 1 (including Tables S10-S14)
    - Run 2 (including Tables S15-S19)
    - CFA excluding Working Memory Updating (including Tables S20-S29)
    - CFA with adjusted priors
      - Gamma (1,1) prior (including Tables S30-S34)
      - Gamma (1,2) prior (including Tables S35-S39)
  - Chimpanzees (including Table S40)
    - Domain-specific models (including Tables S41, S42)
    - CFA (including Tables S43, S44)
- Exploratory Factor Analysis
  - Children (including Tables S45, S46, Figures S31, S32)
  - Chimpanzees (including Table S47, Figures S33, S34)

# **Supplementary Methods**

Table S1. Data on the content validity of the nine EF tasks, with data from the current study and those from several pilot studies shown side-by-side.

| **Function** | **Signature** | **Task** | | | | | |
| --- | --- | --- | --- | --- | --- | --- | --- |
| **Inhibition** | Initial bias for option with visible, inaccessible reward | **Boxes** | | **Cylinder** | | **Grid** | |
|  |  | **Pilot study** | **Current study** | **Pilot study** | **Current study** | **Pilot study** | **Current study** |
|  |  | Children (*n* = 31) 71%^a^ | Children 88%  Chimpanzees 85% | Children (*n* = 30) 73%^a^  Chimpanzees (n = 7): 100%^a^ | Children 94%  Chimpanzees 81% | Children (*n* = 28) 61%^a^ | Children 82%  Chimpanzees 65% |
| **Shifting** | Error pattern | **Boxes** | | **Shelf** | | **Tray** | |
|  |  | **Pilot study** | **Current study** | **Pilot study** | **Current study** | **Pilot study** | **Current study** |
|  |  | Both species: significantly better performance in SD (in which only one dimension is present) than in CD^1^ | Both species: significantly better performance in SD (in which only one dimension is present) than in CD | Shifting errors (out of all errors): Children – pilot study 1 (*n* = 46) 78%, pilot study 2 (*n* = 178) 76%^b^ | Shifting errors (out of all errors):  Children 76%  Chimpanzees 69% | Children (*n =* 43): bias towards dimension “boxes” if children had previous experience with tasks in which boxes were used as hiding places^a^ | / |
| **Working Memory** | Mean performance in single task compared to dual task | **Boxes** | | **Grid** | | **Updating** | |
|  |  | **Pilot study** | **Current study** | **Pilot study** | **Current study** | **Pilot study** | **Current study** |
|  |  | Children (*n =* 36): 1 task 84%, 2 tasks 65%^1^ | Children: 1 task 85%, 2 tasks 56%  Chimpanzees: 1 task 60%, 2 tasks 38% | No pilot study performed | Children: 1 task 94%, 2 tasks 77%  Chimpanzees: 1 task 63%, 2 tasks 65% | Children: 1 task (*n =* 49) 91%, 2 tasks (*n* = 42) 69% [note that these were two independent samples]^a^  Chimpanzees (n = 8): 1 task: 69%, 2 tasks: 40%^2^ | Children: 1 task 88%, 2 tasks 74%  Chimpanzees: 1 task 59%, 2 tasks 51% |

Notes. ^a^Unpublished study. ^b^Manuscript in preparation.

## Participants

For most Executive Functions (EF) tasks, we carried out pilot studies and these confirmed the signatures for content validity, which are reported in Table 1. A complete table that shows the data from the current study and the data from the pilot studies side-by-side is given in Table S1.

### **Children**

Nurseries/schools were recruited by sending out an invitation letter, followed by a phone call a few days later to confirm interest in participation. Nurseries interested in the project received further information about the study requirements, in written form and via personal communication. If nurseries agreed to participate, they were asked to circulate information letters and consent forms to parents/guardians who were given the opportunity to ask questions (personally or via e-mail) before signing up to the study. If they allowed their child to take part, they filled in a consent form online or on paper. A testing window was arranged with the nursery/school according to their availability and the number of children signed up at that time, in order to allow us to see every participating child on twelve (not necessarily consecutive) testing days.

We contacted 108 nurseries/schools, 37 of which (34%) were interested in taking part. Of these, 2 nurseries dropped out before testing commenced as their circumstances changed and they could not accommodate the study, 2 nurseries dropped out because too few parents signed up (two parents in each nursery), and 15 were happy to take part, but the research did not proceed due to the outbreak of the SARS-CoV-2 pandemic. We tested children in 18 nurseries/schools. Testing was completed in ten locations, and had to be stopped in eight due to the SARS-CoV-2 pandemic. Parents received debriefing letters throughout and at the end of the study. Schools/nurseries received a debriefing letter and a verbal debrief at the end of the study, as well as a book for the nursery library. Children received a small toy and a certificate for their participation (in addition to stickers and toys won in the tasks in each session).

We tested 190 children. We had consent from another ten children, but did not test them because they were too shy (2x), below the age of 3 (2x), did not want to take part (1x), stopped the first game and did not want to play on any of the following days (1x), or could not start participation as testing had to stop due to the SARS-CoV-2 pandemic (4x). Of the 190 children, 185 children had valid data on at least one EF task (the remaining 5 only completed the Scrambled Box task or one of the additional tasks) and were included in the Confirmatory Factor Analysis (CFA). Of these, 95 children had valid data on all nine EF tasks and could be included in the Exploratory Factor Analysis (EFA).

The ethnic background was given for 165 of the 190 tested children (87%). Of these 165 children, most parents (128) self-identified their children as “White British” (77%), “White” or “Caucasian” (6, 4%), or “European White” (5, 3%). A further nine children had a “White British” plus a “European White” or a “non-European White” parent (5%). Three children (2%) each were identified as “Indian”, “British Caribbean” and “Mixed”, and one child was identified as “Black British”. One child each was identified as “British + Indian”, “British + African Black”, “White British + Asian + African”, “Pakistani + African”, “Japanese + Polish”, “Chinese”, and “Caucasian + Chinese”.

We received information about postcodes from 168 parents/caregivers (89%), allowing us to estimate the socioeconomic backgrounds our participants came from using the Scottish Index of Multiple Deprivation 2020 (retrieved from https://www.gov.scot/publications/scottish-index-multiple-deprivation-2020/). This index ranks areas according to their deprivation across multiple domains (income, employment, education, health, access to services, crime and housing) and converts the ranks into deciles and vigintiles. Our sample recruited children from across the deprivation range, but there was a bias towards the lesser deprived areas: 50% of the children were in the 7th decile or lower and about 43% of the children for which we had data were in the upper two deciles.

Of the 190 tested children, 161 parents (85%) provided us with information on the highest educational degree obtained in the household. Most parents (114, 71%) had a university degree (56 had an undergraduate degree, 43 a postgraduate degree, 15 a PhD), 27 had higher qualifications (5 with college degree, 11 with HNC, 11 with HND), 4 parents had A levels or equivalent, 8 a GCSE or equivalent, 1 parent had no qualifications, and for 7 parents the reported information was unclear.

Children’s receptive vocabulary was assessed with the British Picture Vocabulary Scale III (BPVS III). We tested 156 children, from which we had to drop 7 from analysis: for 5 children, no basal set could be established because performance was too low (3x) or because children answered wrongly on purpose (2x). One child stopped the game and for one child no standardized score could be calculated because the raw score was too low. We had 149 valid data on the BPVS III (79 girls, 70 boys). A linear regression with the standardized score as dependent variable and age in months and testing location as independent variables revealed not only a significant effect of age, but that children from the urban area scored on average 5.62 (95% CI [1.72; 9.52]) units higher than children from the rural area (χ2(1) = 1162.1, p = .005).

A subset of children (n = 105) was additionally tested on a further six tasks, measuring Theory of Mind (Sally Ann Task, Smarties Task), Associative Tool use, Causal reasoning (“Foil”, “Bing”) and Creating Overhypotheses (“Worlds”; not reported here). These tasks were administered as a second task following an EF task. Of these children, 13 were also tested on an additional Inhibition task (Stop-box task). However, due to apparatus malfunction we decided to drop this task from further testing and to not analyze the data from these children.

Parents/guardians could indicate on the consent form whether they agreed to fill in the Behaviour Rating Inventory of Executive Function – Preschool Version (BRIEF-P^3^). They could do so in paper format or via phone. We received questionnaires for 115 children.

Children were tested individually but were separated from the others for no longer than 50 minutes. We only administered one (part of an) EF task per day, with the exception of about 12 children for whom some EF tasks had to be administered in pairs within a single day (with a break in between) due to time constraints by the nursery that forced us to complete data collection within a shorter amount of days. Children were rewarded with stickers and small toys regardless of success.

Weekends, holidays, child absences due to sickness, and nursery activities which meant that we could not visit (e.g., inspections, forest walks) caused the days between testing sessions to vary between participants. We calculated the intervals between testing sessions as the number of days between two adjacent sessions, including the day on which the next session took place (we considered 10 intervals, see Table S2). These intervals ranged from 0 days (when we had to administer two EF tasks within a single day, see previous paragraph) to 68 days (this was the case for two children due to summer holidays). The minimum number of days between test sessions ranged from 0 to 27, the maximum number ranged between 1 and 68. For each child, we calculated the mean interval length across all 10 intervals. Mean interval length ranged from 1 to 33.5 days (the child with 33.5 days was only tested twice and then dropped out of the study). The average of this mean interval range *across* children was 5.64 days (mode: 2.9 days, median 4.3 days).

Table S2. Overview of length of intervals between testing sessions for the child sample.

| **Interval (number of children)** | **Mean number of days ± sd, range, mode, median** |
| --- | --- |
| WM Updating – Shifting Shelf 1 (177) | 5.45 ± 8.67, 1-60 days,  mode = 1 day, median = 2 days |
| Shifting Shelf 1 – Shifting Shelf 2 (167) | 7.09 ± 8.09, 1-44 days,  mode = 1 day, median = 4 days |
| Shifting Shelf 2 – Inhibition Grid (166) | 4.41 ± 6.58, 1-47 days,  mode = 1 day, median = 3 days |
| Inhibition Grid – Shifting Tray (151) | 5.87 ± 7.92, 0-60 days,  mode = 1 day, median = 3 days |
| Shifting Tray – WM Boxes (149) | 4.91 ± 6.55, 0-50 days,  mode = 1 day, median = 3 days |
| WM Boxes – Inhibition Cylinder (147) | 5.88 ± 9.19, 0-68 days,  mode = 1 day, median = 3 days |
| Inhibition Cylinder – Shifting Boxes 1 (144) | 4.83 ± 8.78, 0-50 days,  mode = 1 day, median = 2 days |
| Shifting Boxes 1 – Shifting Boxes 2 (139) | 6.16 ± 11.38, 0-68 days,  mode = 1 day, median = 3 days |
| Shifting Boxes 2 – Inhibition Boxes (133) | 4.59 ± 7.38, 0-50 days,  mode = 1 day, median = 3 days |
| Inhibition Boxes – WM Grid (130) | 6.00 ± 10.54, 0-61 days,  mode = 1 day, median = 2 days |

### **Chimpanzees**

We tested 55 chimpanzees in two sanctuaries (Sweetwaters Chimpanzee Sanctuary, Ol Pejeta Conservancy, Kenya: N = 30; Ngamba Island Chimpanzee Sanctuary, Uganda: N = 25). The Sweetwaters Chimpanzee Sanctuary lies within the Ol Pejeta Conservancy (Laikipia, Kenya) and is a refuge for orphaned and seized chimpanzees. At the time of the study, 39 chimpanzees of various ages lived in the sanctuary in two social groups. Group 1 consisted of 24 chimpanzees (13 females, 11 males). Group 2 comprised of 15 chimpanzees (8 females, 7 males). We tested chimpanzees of both groups. Ngamba Island Chimpanzee Sanctuary is located in Lake Victoria (Uganda) and is a refuge for 49 orphaned and confiscated chimpanzees.

Participants were individually tested but were separated from the others for no longer than 30 minutes. There was only one test session per day and individual. The chimpanzees were fed multiple times a day with fresh fruits and vegetables and the research did not interfere with the feeding schedules.

We administered one to two (parts of an) EFs task per day in a fixed order across individuals. We never conducted more than one task aiming at measuring the same executive functions on the same day. We aimed to test on consecutive days, which was not always possible. The interval ranged from 1 to 23 days (the latter case refers to an outlier; a case in which an individual destroyed multiple cylinders in the inhibition cylinders task and we decided to first test the remaining individuals with these materials before further testing this individual). For each chimpanzee, we calculated the mean number of days between test sessions (range of mean individual values: 1.06 – 3.27 days). Across chimpanzees, the average of the mean number of days between testing sessions was 1.34 days (mode: 1.25 day, median 1.29 day). The minimum number of days between tests was 1 day for each individual, the maximum number of days ranged from 2 to 23 between individuals.

Data of all 55 individuals were included in all analyses except for the EFA (age range: 5-35 years; median age: 20 years; 29 females; 24 males). Forty-eight chimpanzees completed all nine tasks and were included in the EFA.

## Materials

### **Children**

Stickers (collected on sticker scenes and in sticker albums) and small toys (animal figures, bubble wands, tattoos, bracelets, gliders, pens, small plush toys) were used as rewards in the tasks (see Table S3 for information on which rewards were used in which task).

For the first task (Scrambled box task) as well as for the longer tasks (Shifting Shelf Session 2, Shifting Tray, Shifting Boxes) children chose a sticker scene for which they could collect stickers. These scenes were purchased from Baker Ross (for an example see Fig. S1, retrieved from https://www.bakerross.co.uk/sealife-sticker-scenes-1?&gclid=Cj0KCQiA7NKBBhDBARIsAHbXCB5DUoHyW80fi1VRN8SR1Ya4MrlGpBy-ir50hJ9IUgqe6_tjvmurIN0aAibkEALw_wcB) and consisted of between 20 to 30 stickers per sheet. The sheets were used as they came with a large amount of stickers and allowed children to engage in a story, which helped to maintain children’s interest and attention for a prolonged time. In shorter tasks, children collected loose stickers which they could stick into a sticker album. For this, children were allowed to choose one of a variety of different themed sticker albums in their second testing session.


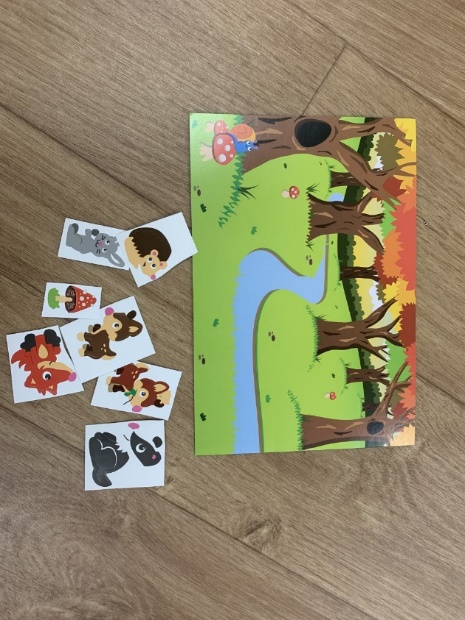


Fig. S1. Example of a sticker scene that children could choose for some of the tasks.

After completion of each testing session and regardless of success, children could choose a sticker from a sticker box (a box containing smaller, cut stickers of a wide variety) and place it on a “reward sheet” (either a forest scene or an underwater scene, one of which they were invited to choose on day 1; Fig. S2) to help them track their progress through the study. Children were informed that the games would end when they had collected stickers for each space on the reward sheet, and that they could then take the reward sheet and their sticker album home. The experimenter kept the sticker album and the reward sheet until the child completed the test battery (otherwise children would take them home and forget to bring them back for the next session). The sticker scenes and small toys that children received in the tasks could be taken home on the same day. For this, the experimenter or a research assistant helped the child place their prizes into their nursery/school bag or tray. Children were discouraged from taking the prizes to the play area in order to avoid jealousy from other children.


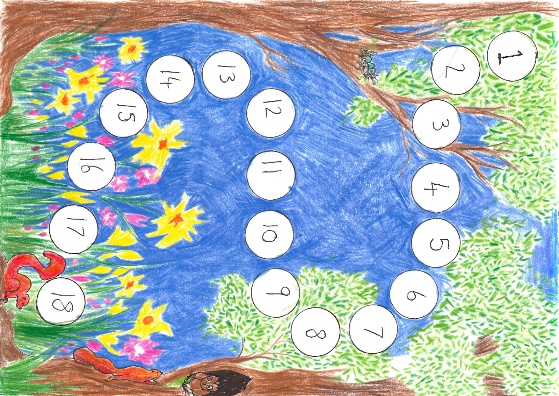

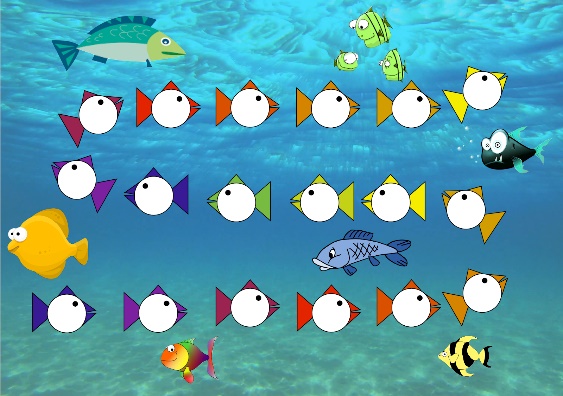


Fig. S2. The two different reward sheets used for the test battery. Children could choose one on their first testing day.

Inhibition Boxes

We used two acrylic boxes (w x d x h: 16 cm x 9 cm x 22 cm), one transparent and one opaque (Fig. S3). Each box contained two compartments. In the lower compartment, a filling material (blue cotton wool) served as a potential hiding place for a plastic egg with a sticker inside. The filling material could be extracted through an opening in the front side of the boxes. The upper compartment was divided from the lower compartment by a plastic ceiling. On top of the ceiling (i.e., in the upper compartment), there was a thin layer of cotton wool and a sticker which were inaccessible to the child. The upper compartment was only visible in the transparent box. The opaque box always contained a plastic egg in the filling material that could be obtained by removing the filling material. We used 12 pre-filled plastic surprise eggs to reduce inter-trial-time as the experimenter did not have time to rebait the eggs. A cardboard occluder was used to occlude the baiting of the boxes.


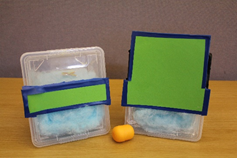


Fig. S3. Material for the Inhibition Boxes task (child version).

Inhibition Cylinder

The setup was on the floor so that children could move around the array more easily. We used a large green Correx plastic fluted board (w x d: 85 cm x 60 cm), a small green Correx plastic fluted board (w x d: 42.5 cm x 21 cm) for the warm-up trial, and 24 cylinders (9.5 cm diameter), with each cylinder consisting of two stacked plastic transparent dessert cups put upside down on the board (so that the bottoms were facing up; Fig. S4). The bottom cup was opaque, so that children could not see inside the cylinder. The top cup - sitting on the first cup - was transparent, allowing children to see what was inside the small gap between the two cups (but inaccessible). This was either an attractive sticker (for 12 of the cylinders) or an unattractive small dot (for the other half of the cylinders). The two cups were glued to each other so that it was impossible for children to reach the sticker inside.

The 24 cylinders were distributed on the large board on the floor. The setup looked random, but the location of the cups was fixed and the same for each child and indicated by small signs on the board. The distance between adjacent cylinders on the board was ca 4-8 cm. There were two types of cylinders: 12 cylinders showed attractive, but inaccessible stickers of a certain theme which the children could choose before the task began to ensure that the stickers were highly desirable (e.g., Spiderman, Dinosaurs, Disney Princesses). However, when these cups were lifted, there would be a small, unattractive dot sticker lying underneath the cup on the board. The other 12 cylinders showed unattractive, and also inaccessible, blue dot stickers. However, when these cups were lifted, there was one of the attractive stickers from the sticker theme underneath. For the warm-up, we used a small green board with a sticker cup and a dot cup on top.


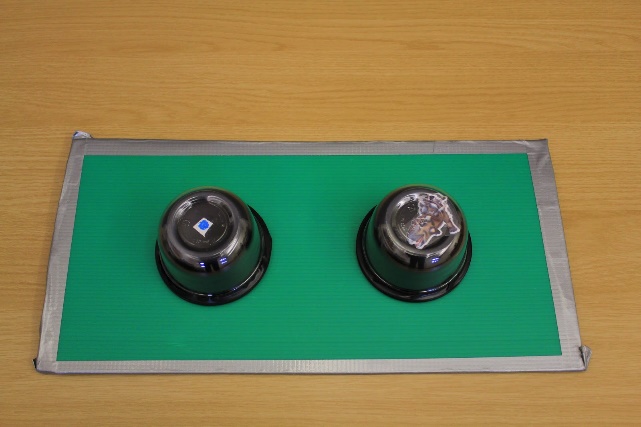

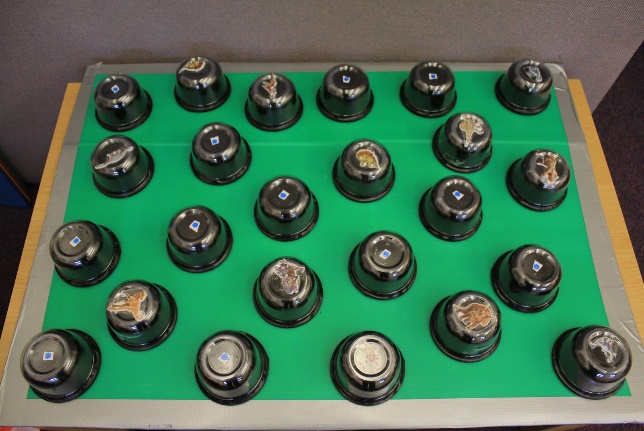


Fig. S4. Material for the Inhibition Cylinder task (child version). Left: Warmup; right: Test.

Inhibition Grid

We used a transparent acrylic flap-door grid with 16 (4 x 4) compartments (w x d x h: 30 cm x 11 cm x 31 cm) into which boxes with opaque or transparent flap doors could be inserted (Fig. S5). Boxes could be inserted to any compartment of the apparatus via the back of the apparatus. Before each trial, outside of the child’s view (using a cardboard occluder), seven transparent and six opaque boxes were arranged on the grid in a prefixed, pseudorandom order: In each quadrant of the grid there was always one transparent and one opaque box. In addition, before each trial, all of the boxes were baited outside the child’s view with one reward (an animal toy) per compartment. Only the opaque doors could be opened, while the transparent doors were blocked (by means of a screw). A cardboard occluder was used to hide the box at the beginning of the trial as well as the rebaiting for trial 2.


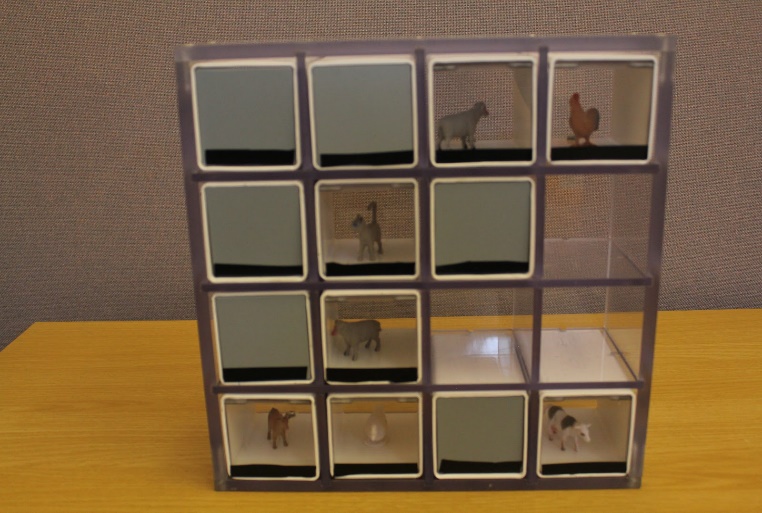


Fig. S5. The Inhibition Grid task (child version), with opaque and transparent boxes inserted and baited with animal toys.

Shifting Boxes

We used ten cardboard boxes (w x d x h: 8.5 cm x 4.5 cm x ~ 11 cm) with a small opening at the front. The upper part of the boxes varied in colour, material and shape (Fig. S6). The boxes were filled with varying materials (see below) that could be retrieved through the opening in the front. Stickers were used as rewards which were hidden inside the filling material. A cardboard occluder was used to hide the baiting process.

For the Simple Discrimination learning and Reversal learning phases, we used two visually distinct boxes (one with golden circles, one with blue waves on the top) and the same filling material (red tissue paper). For the Compound Discrimination learning, we used two identical sets of two visually distinct boxes (one with silver stripes, one with brown paper pieces on top) and two filling materials (green bast fibre ribbon, natural bast fibre). For the Extradimensional Shift phase, we used two identical sets of two visually distinct boxes (one with pieces of lace, one with cork circles on top) and two filling materials (lightgreen felt, striped table napkin).


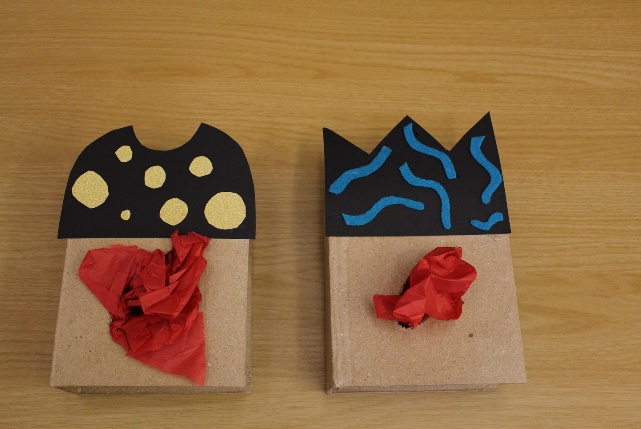

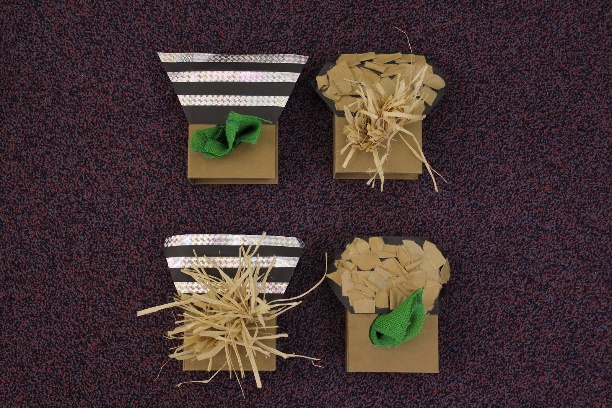


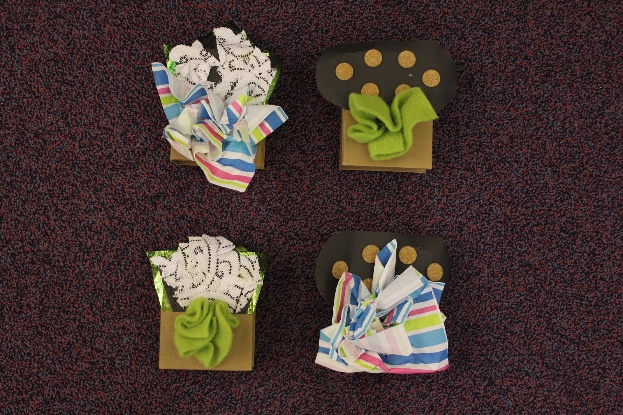


Fig. S6. Material for the Shifting Boxes task (child version). Top left: Simple Discrimination and Reversal; top right: Compound Discrimination; bottom: Extradimensional Shift.

Shifting Shelf

We used two visually distinct platforms (“shelves”; blue and green) made from felt (w x d x h: 33 cm x 24cm x15 cm), a cardboard occluder, stickers as rewards, and 12 cups (8-11 cm diameter) which were placed upside down on the shelves (Fig. S7):

- For the green platform:
  - two target cups: green, orange (green baited)
  - two distractor cups for training 2a and 2b: blue, red
  - two distractor target cups for the test phase: pink, yellow
- For the blue platform:
  - two target cups: pink, yellow (pink baited)
  - two distractor cups for training 2a and 2b: blue, red
  - two distractor target cups for the test phase: green, orange

Cups that served as distractors (i.e., cups that were not baited) were closed with a lid and could not be opened.


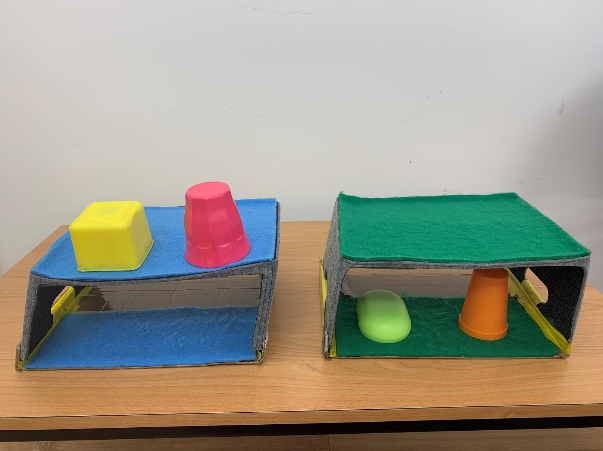

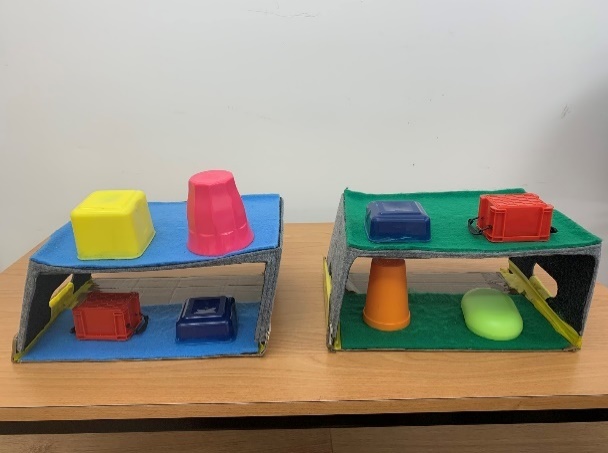


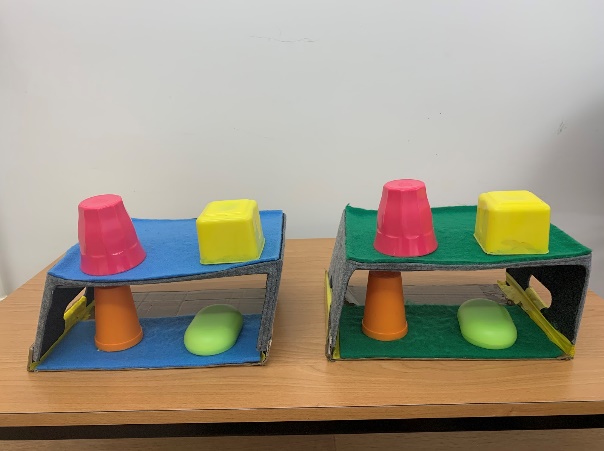


Fig. S7. Material for the Shifting Shelf task (child version), as seen in the test phase from the child’s view. Top left: Training 1; top right: Training 2; bottom: Test.

Shifting Tray

We used two clear acrylic trays (w x d x h: 20.3 cm x 20.3 cm x 5.1 cm), each filled with one of two substrates (green play sand, red shredded paper; Fig. S8). On top of the substrate in each tray there was one of two boxes (yellow, purple; the same boxes as used in the Scrambled box and WM Updating tasks). The lids were removed from the boxes for this task and the boxes were placed upside down (with the opening facing downwards) onto the substrates. The trays were placed on a large transparent board to facilitate moving the trays towards the child and to avoid spillage of the sand. Stickers were used as a reward. A cardboard occluder was used to hide the baiting process. The red shredded paper was the stimulus predictive of the reward.


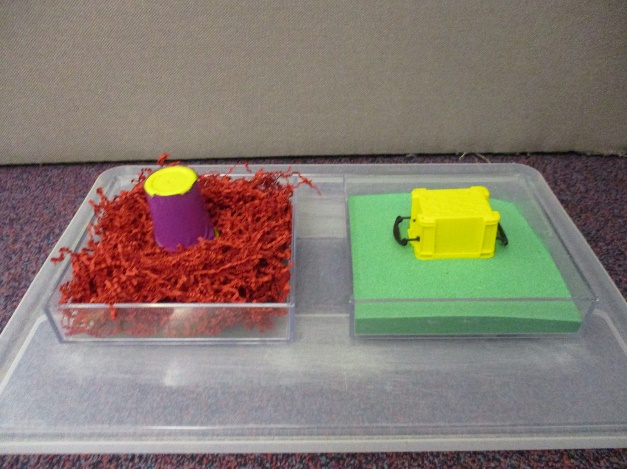


Fig. S8. Material for the Shifting Tray task (child version).

Working Memory Boxes

We used eight identical brown cardboard boxes (w x d x h: 9 cm x 4 cm x 12 cm) which were placed – in two groups of four boxes each – onto two white and blue polystyrene platforms (w x d: 60 cm x 9.5 cm; Fig. S9). Each box had a hole in the front and was filled with white tissue paper. Stickers were used as a reward, which could be inserted into the boxes through a hole in the back. A cardboard occluder was used to hide the boxes during the retention intervals.


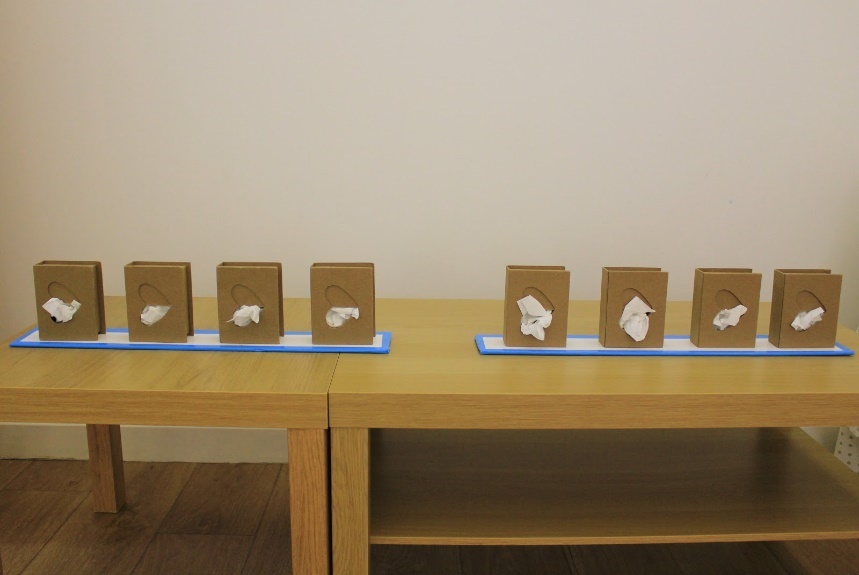


Fig. S9. Materials used in the WM Boxes task (child version).

Working Memory Grid

We used a transparent acrylic grid with 16 (4 x 4) compartments (w x d x h: 30 cm x 11 cm x 31 cm) and 16 identical, brown cardboard boxes that fit exactly into the compartments, animal toys as rewards and an occluder. The cardboard boxes could be inserted into the grid via the back of the apparatus. We also used a cardboard platform (w x d: 60 cm x 23 cm) with three identical, green paper maché boxes (w x d x h: 9 cm x 7 cm x 4 cm) and star stickers as rewards for the distractor task (Fig. S10).


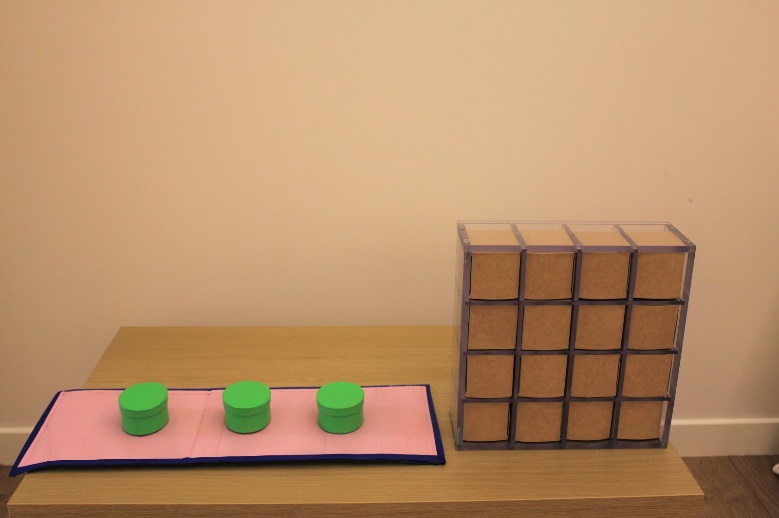


Fig. S10. Materials used in the WM Grid task (child version), from the child’s view.

Working Memory Updating

We used two adjacent cardboard platforms (w x d: 82 cm x 20 cm), two identical sets of four visually distinct, opaque boxes, one cardboard occlude, and stickers as rewards (Fig. S11).


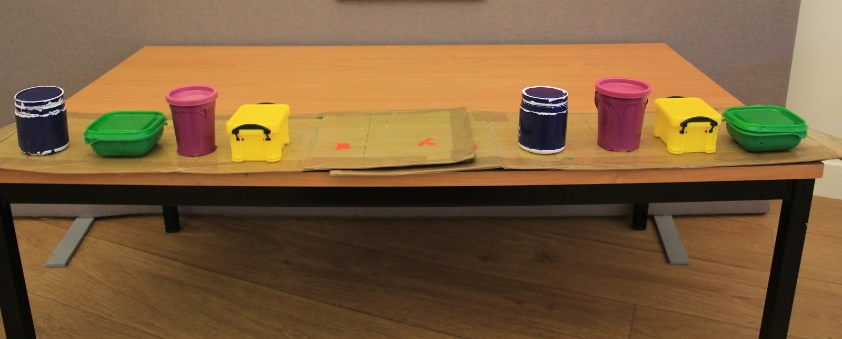


Fig. S11. Materials used in the WM Updating task (child version).

Scrambled Box task (warm-up for WM Updating)

We used two adjacent cardboard platforms (w x d: 82 cm x 20 cm), eight plastic boxes of different shapes and colours (blue, red, green, yellow, orange, white, light blue), stickers as rewards, and a cardboard occluder to hide the scrambling events (Fig. S12).


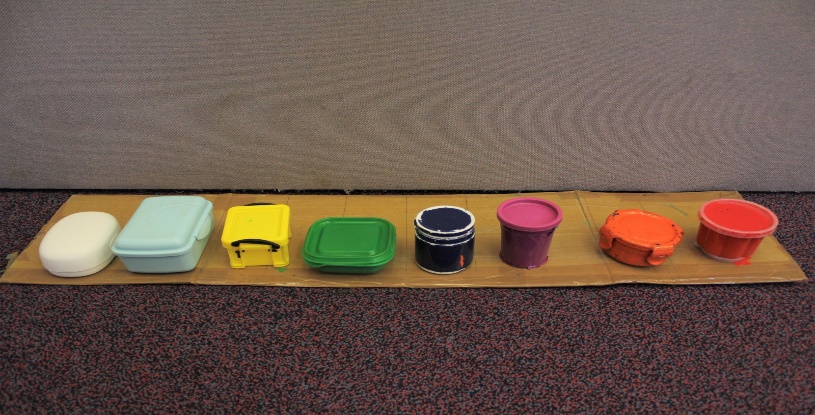


Fig. S12. Material for the Scrambled Box task.

### **Chimpanzees**

We used high value food rewards for all tasks including pieces of apple, banana, and peanuts (see Table S4).

Inhibition Boxes

We used a sliding platform on top of a table outside the enclosure and two boxes made of Perspex (h x w x d: 21 cm x 16 cm x 10 cm), one transparent and one opaque (Fig. S13). Each box was fixated on top of a small plastic tray (d x w: 35 cm x 27 cm). Besides, we used an occluder for the baiting of the boxes. The boxes contained two compartments. In the lower compartment (h x w x d: 11 cm x 16 cm x 10 cm) filling material (toilet paper) served as potential hiding place for the food reward (1/16 apple piece). The filling material could be extracted though an opening in the front side of the boxes (3 x 3 cm). The upper compartment (h x w x d: 10 cm x 10 cm x 6.5 cm) was only visible in the transparent box. There was a piece of food (1/2 apple) on the intermediate ceiling in the upper compartment inaccessible to the subject. The opaque box always contained a reward hidden in the filling material that could be obtained by removing the filling material.


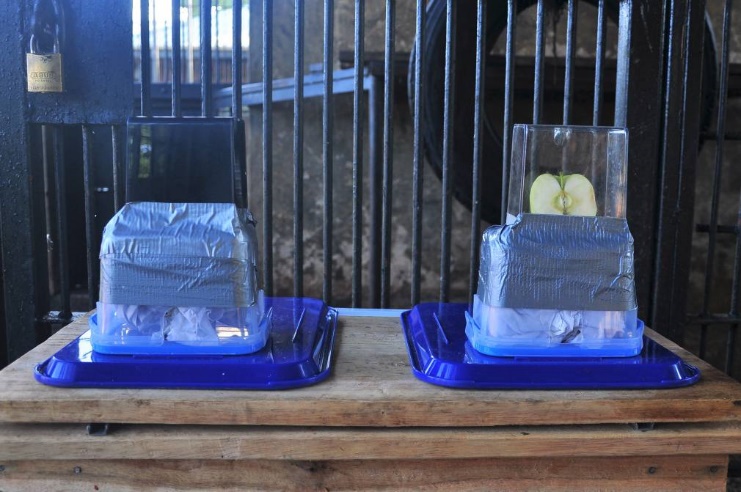


Fig. S13. Materials for the Inhibition Boxes task (chimpanzee version) shown from the subject’s perspective.

Inhibition Cylinder

We distributed 24 polycarbonate cylinders (h x d: 4 cm x 6 cm) on the floor of the enclosure on a 6 x 8 grid which covers an area of 120 x 150 cm (Fig. S14). The distance between adjacent cylinders on the grid was ca 20 cm. There were two types of cylinders: 12 transparent and 12 opaque (green) cylinders. The distribution of the cylinders on the grid was pseudo-random with the restriction that there were two cylinders of each type in any of the eight columns of the grid. The transparent cylinders were sealed and contained visible but inaccessible food rewards (a peanut inside its shell). The opaque cylinders were open (they did not have a bottom) and baited with the same type of food. Two cameras were used to videotape chimpanzees’ search behaviour.


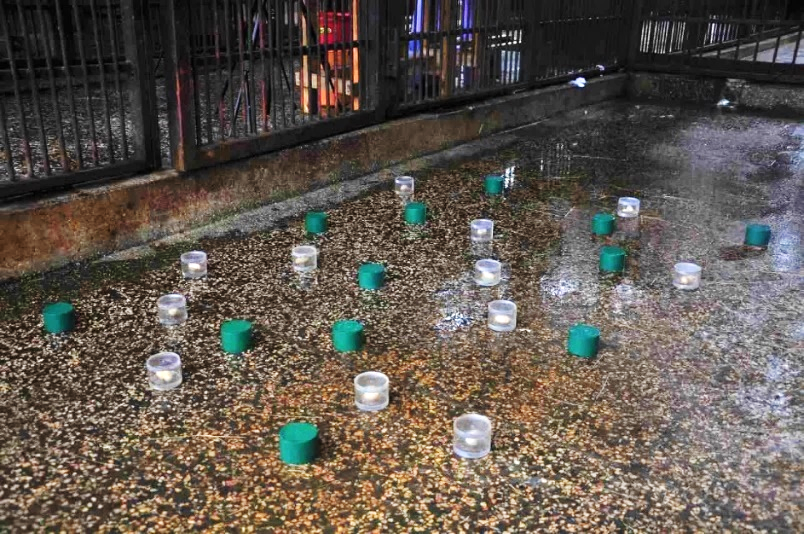


Fig. S14. Materials for the Inhibition Cylinder task (chimpanzee version).

Inhibition Grid

We used a flap-door apparatus (h x w x d: 48 cm x 45 cm x 16 cm) with 16 compartments (4 x 4) with opaque and transparent flap doors (10 cm x 7 cm) on top of a table outside the enclosure (Fig. S15). All compartments of the apparatus could be baited via the back of the apparatus. Six transparent and seven opaque doors were arranged on the grid. All of these 13 compartments were baited outside the subject’s view at the beginning of a trial with a quarter piece of banana. The transparent doors (but not the opaque doors) were blocked (by means of a screw) and could not be opened. Subjects could obtain rewards behind opaque flap-doors.


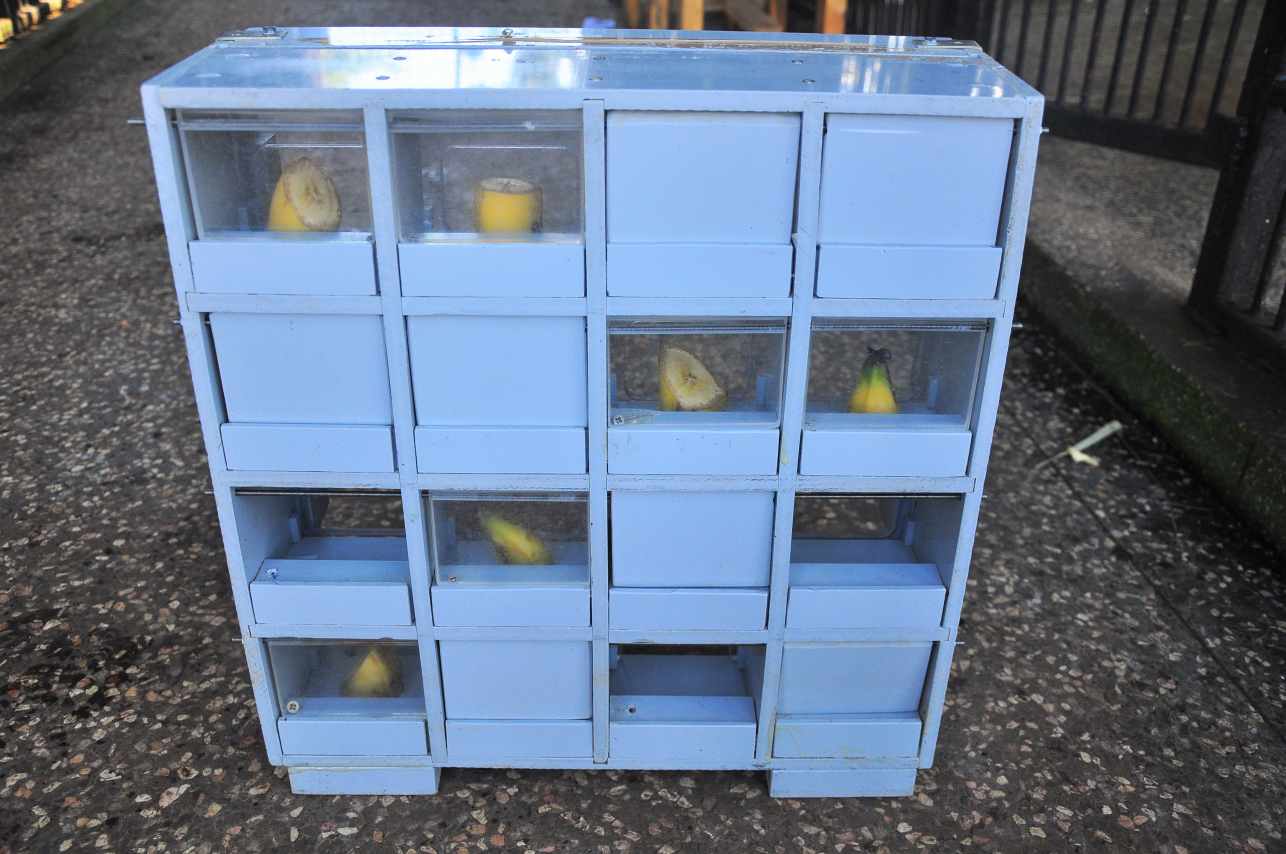


Fig. S15. Materials for the Inhibition Grid task (chimpanzee version) shown from the subject’s perspective.

Shifting Boxes

We used a sliding platform attached to the wall of the enclosure, two identical training boxes (boxes h x w x d: 16.5 cm x 9.5 cm x 6.5 cm; hole in back: 3 cm) plus four visually distinct test boxes (h: 18 - 20 cm, w: 15 - 17 cm, d: 7.5 cm, back opening: 6 x 4 cm; foot with 1 cm wider baseplate on each side to fix them to the panel; Fig. S16). The boxes were opaque except for a transparent lower front panel made of Perspex. We filled the boxes with varying natural materials (SD/SR: brown paper vs toilet paper; CD: saw dust/wood shavings vs cotton; ID: mud/soil vs leaves; ED: palm fibre vs straw) that could be retrieved through small openings in the front (3.5 x 3.5 cm). The food reward (1/16 apple piece) was hidden inside the filling material of one of the boxes. Additionally, the upper part of the boxes varied in colour, material and shape. The boxes were presented on a polycarbonate panel (w x d: 64 cm x 40 cm) including a centrally located add-on that could hold the chosen box in place (due to the wider baseplate) for the subject to extract its content.


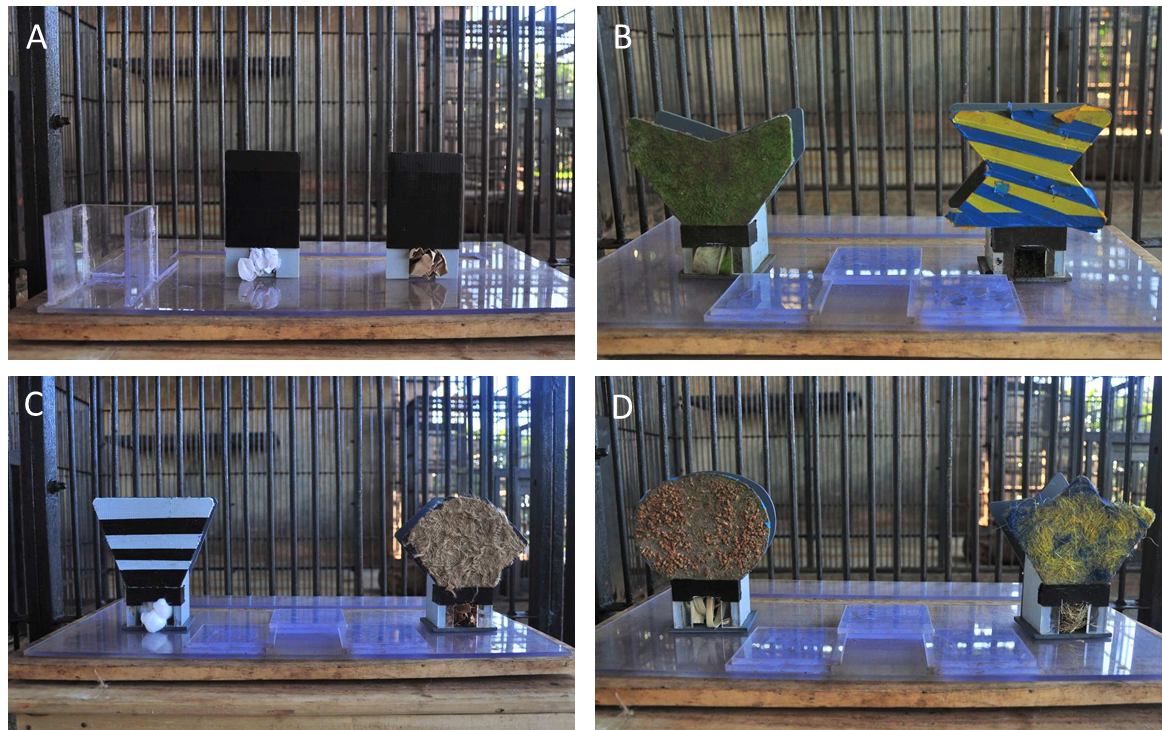


Fig. S16. Materials for the Shifting Boxes task (chimpanzee version) shown from the subject’s perspective. A) SD / SR version; B) ID version; C) CD version; D) ED version.

Shifting Shelf

We used two visually distinct shelves (a black and a white monitor stand; h x w x d: 18 cm x 50 cm x 33 cm) on top of two adjacent sliding platforms attached to the wall of the enclosure (Fig. S17). We used in total six distinct cups as hiding locations for a food reward (1/12 banana piece). On every shelf, there were four cups, two cups on the top (brown box, w x d x h: 8 cm x 8 cm x 7 cm; vs blue cylinder, h: 7 cm, diameter: 8 cm) and lower shelf (green mug, w x d x h: 11 cm x 7 cm x h 8 cm; vs chrome cup, h: 7 cm, diameter: 9 cm), respectively. The chrome cup was baited on platform 1 (lower shelf) and the brown box (upper shelf) was baited on platform 2. Cups that served as distractors (i.e. cups that were not baited) were closed with a lid (and never opened in front of the subjects). During the second training phase, we used two additional distractor boxes, a yellow cylinder (diameter: 10 cm; h: 6 cm) and a grey box (w x d x h: 11.5 cm x 8 cm x 7 cm).


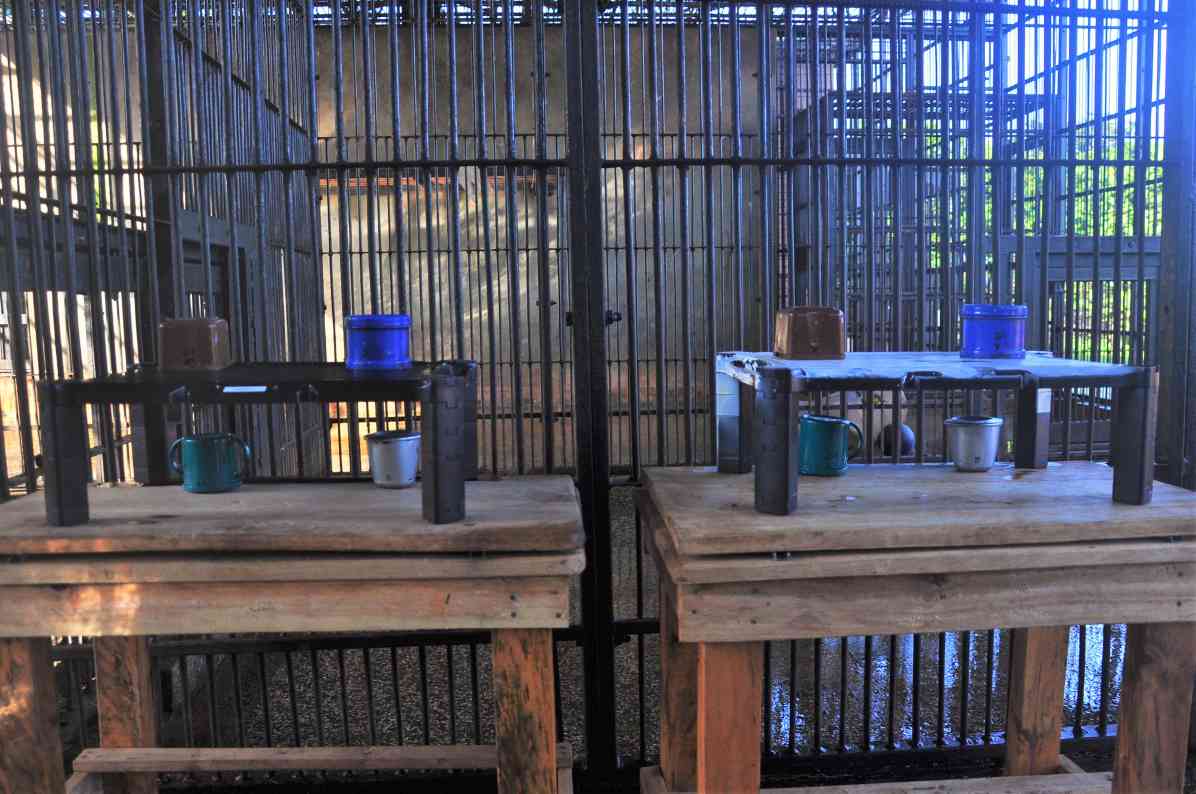


Fig. S17. Materials for the Shifting Shelf task (chimpanzee version) shown from the experimenter’s perspective.

Shifting Tray

We used a sliding platform attached to the wall of the enclosure, two trays (w x d x h: 5 cm x 30 cm x 20 cm) filled with two different filling materials (toilet paper vs brown paper), and two distinct boxes (green box: w x d x h: 5.5 cm x 5.5 cm x 4.5 cm; orange heart: w x d x h: 7.5 cm x 6 cm x 3.5 cm; Fig. S18). On top of each tray was a different box from the working memory updating task (i.e., these boxes had been associated with food before). The toilet paper was the baited stimulus. We used apple pieces (1/16) as rewards.


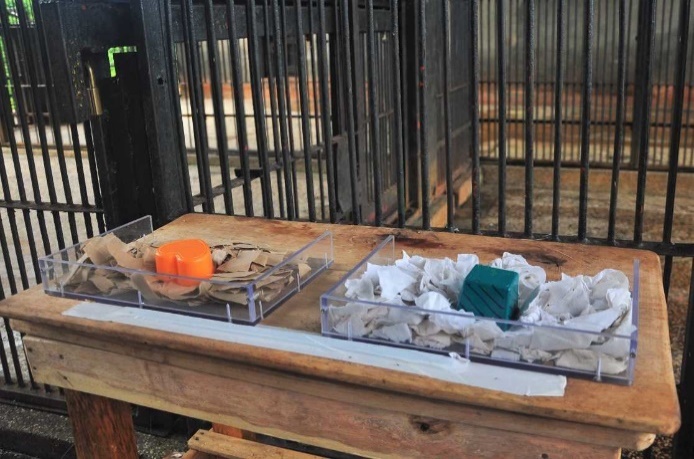


Fig. S18. Materials for the Shifting Tray task (chimpanzee version) shown from the experimenter’s perspective.

Working Memory Boxes

We used two adjacent sliding platforms on top of tables outside the enclosure, eight identical opaque boxes (w x d x h: 16.5 cm x 7.5 cm x 7.5 cm) with an opening in the front side (w x d: 3.5 cm x 3.5 cm) filled with toilet paper, and one occluder (free-standing occluder: h x d x w: 38 cm x 22 cm x 64 cm; Fig. S19). All boxes could be baited through a hole (diameter: 3cm) in the back. The boxes were presented on a polycarbonate panel (w x d: 64 cm x 40 cm) including a u-shaped add-on (h x d x w: 10 cm x 10 cm x 8.5 cm) located at the right (left platform) or left corner (right platform) of the panel (at the side closest to the subject) that could hold the chosen box in place for the subject to extract its content.


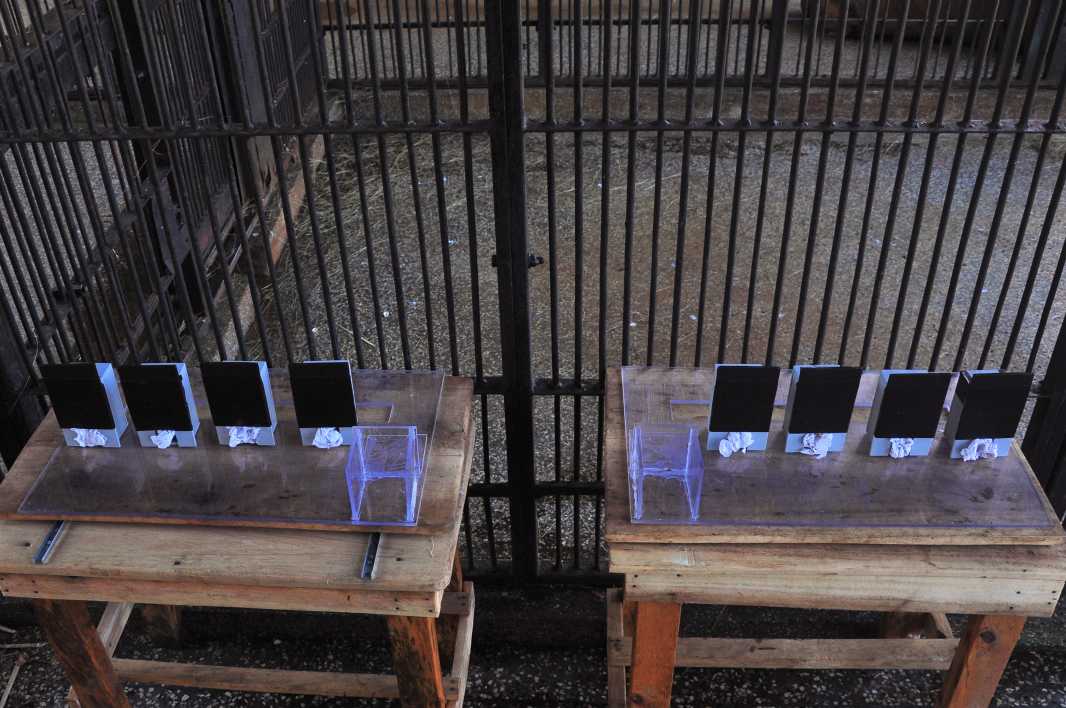


Fig. S19. Materials for the WM Boxes task (chimpanzee version) shown from the subject’s perspective.

Working Memory Grid

We used two adjacent sliding platforms on top of two tables outside the enclosure and a flap-door apparatus (h x w x d: 48 cm x 45 cm x 16 cm) with 16 compartments (4 x 4) with opaque flap doors (10 cm x 7 cm; Fig. S20). All compartments could be baited via the back of the apparatus. For the secondary task, we used three identical blue, opaque cups (h: 10.5 cm, diameter: 7 cm).


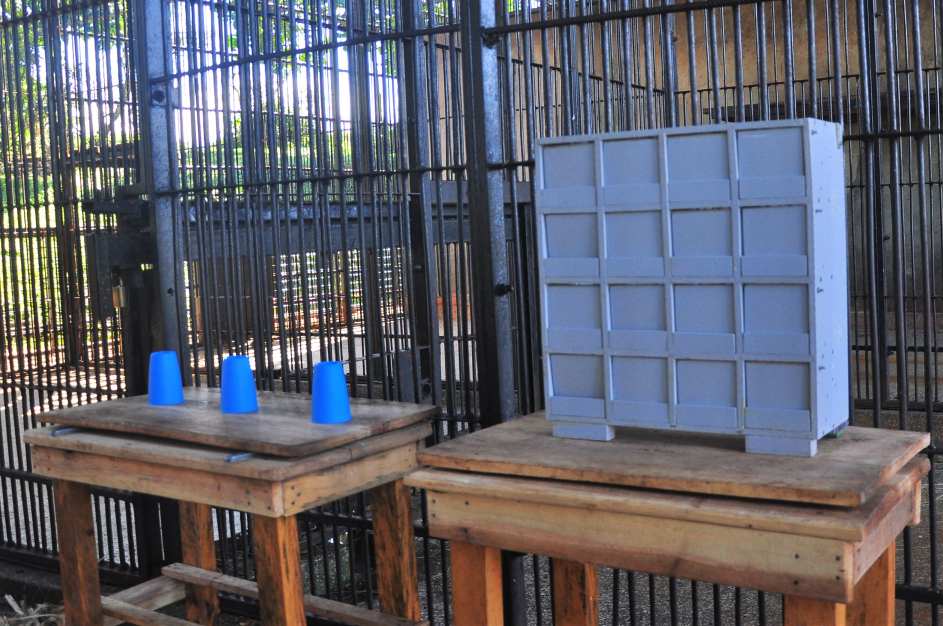


Fig. S20. Materials for the WM Grid task (chimpanzee version) shown from the subject’s perspective.

Working Memory Updating

We used two adjacent sliding platforms on top of tables (w x d x h: 76 cm x 40 cm x 62 cm) outside the enclosure, opaque boxes (2 sets of 4 visually distinct boxes plus one additional box: brown bottle: w x d x h: 5 cm x 5 cm x 7.5 cm; green box: w x d x h: 5.5 cm x 5.5 cm x 4.5 cm; orange heart: w x d x h: 7.5 cm x 6 cm x 3.5 cm; yellow box: w x d x h: 6 cm x 6 cm x 4 cm; additional box: w x d x h: blue box: 5 cm x 5 cm x 5 cm), and one occluder (warm-up occluder: h x w: 43 cm x 66 cm; free-standing occlude for test trials: h x d x w: 38 cm x 22 cm x 64 cm; Fig. S21).


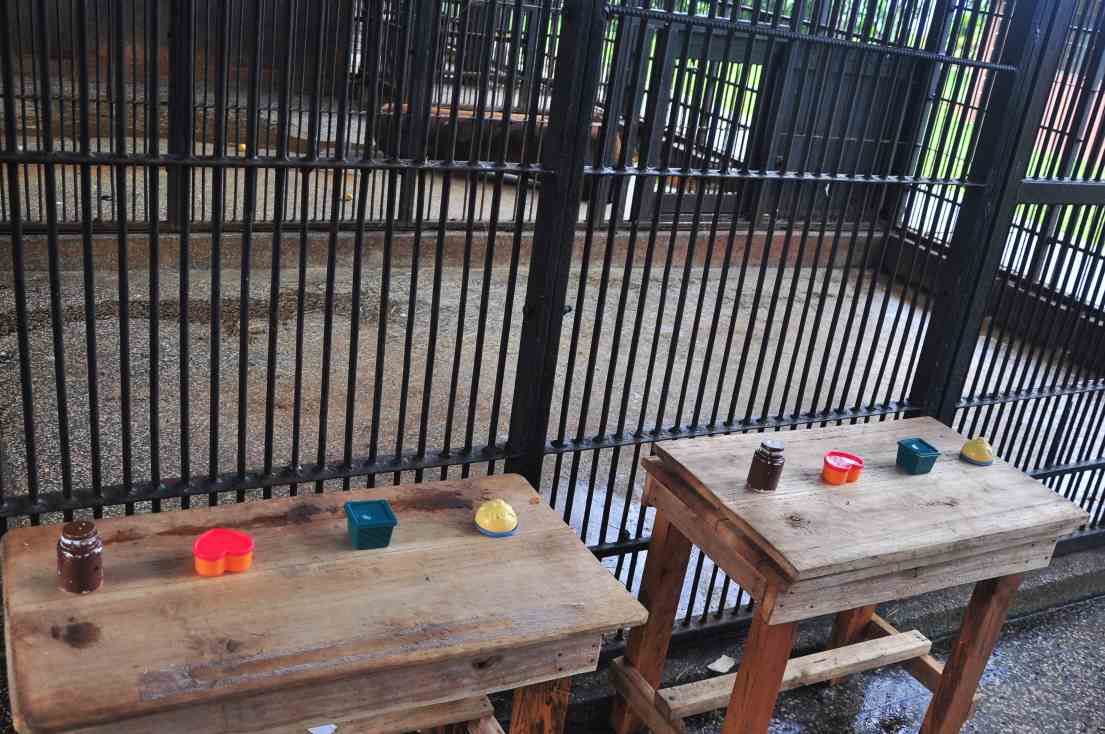


Fig. S21. Materials for the WM Updating task (chimpanzee version) shown from the subject’s perspective.

## Design

### **Children**

The intended order and combination of EF and additional tasks is presented in Table S3. The order of the EF tasks was the same for each participant and determined in a way that no two tasks of the same domain were administered in consecutive sessions. The tasks were distributed over twelve (non-consecutive) testing days. In addition, all children were administered the British Picture Vocabulary Scale III (BPVS III) to assess their verbal ability (usually on day 2, 3 or 4). A subset of participants was tested on additional tasks (not reported here): 105 children (rural areas) were tested on an additional six tasks, assessing Theory of Mind, Associative Tool use, Causal reasoning, and Creating Overhypotheses. More flexibility was required to administer these additional tasks: Occasionally, contrary to the planned pairing of tasks the experimenter had to alter the testing schedule if a task took a relatively long time to complete (e.g., because children needed more trials to reach a learning criterion, reacted very slowly, were very chatty, etc.). On these occasions additional tasks were postponed to a later time in the day or another day to ensure that children would not get fatigued or unmotivated. If time and the nursery environment allowed and children were still motivated, the additional task was administered on the same day, after a break (i.e., the child did the EF task, then went back to nursery, the researcher tested other children, and then invited the child for a second game later in the day). Time restrictions often hindered researchers from administering two tasks a day, resulting in them seeing the children on more than 12 testing days (i.e., up to 16 days). Therefore, the order of the additional tasks was not (and was never intended to be) fixed across children.

Table S3. Overview of the administration of the nine EF and the additional tasks for children in their chronological order over the 12 testing days.

| **Day** | **Task order** | **Session description** | **Max duration** | **Additional tasks** |
| --- | --- | --- | --- | --- |
| 1 | WM Boxes (part 1)^1^ | Warm-up: Scrambled box task (2 trials) | 15 min | Sally Ann |
| 2 | WM Boxes (part 2)^1^ | Test (2 trials) | 15 min | Smarties |
| 3 | Shifting Shelf (part 1)^1^ | Training 1 and 2a | 20 min | BPVS^4^ |
| 4 | Shifting Shelf (part 1)^1^ | Training 2b and Test | 30 min |  |
| 5 | Inhibition Grid^2^ | Test (2 trials) | 15 min | Foil^1^ |
| 6 | Shifting Tray^1^ | Test | 15 min | Associative Tool use^3^ |
| 7 | WM Updating^1^ | Warm-up and Test | 10 min | Worlds |
| 8 | Inhibition Cylinder^1^ | Warm-up and Test | 12 min | Bing^1^ |
| 9 | Shifting Boxes (part 1)^1^ | Simple discrimination and Reversal | 20 min | (Stop box part 1^5^) |
| 10 | Shifting Boxes (part 1)^1^ | Compound discrimination and Extradimensional shift | 40 min |  |
| 11 | Inhibition Boxes^1^ | Test | 15 min | (Stop box part 2^5^) |
| 12 | WM Grid^2^ | Warm-up and Test | 25 min |  |

Notes: ^1^Reward: stickers (amount depended on number of correct trials). ^2^Reward: animal toys (amount depended on number of correct trials). ^3^Reward: Small toy (regardless of success). ^4^The BPVS was administered to all children, not just to the subsample of children who received the rest of the additional tasks. ^5^This task was abandoned after we tested the first 13 children due to apparatus malfunction.

### **Chimpanzees**

The order and combination of EF and additional tasks is presented in Table S4. The tasks were distributed over 18 consecutive testing days and always administered in the same order. A subset of 28 chimpanzees (Sweetwaters sanctuary) was tested on tasks assessing Overimitation, Associative Tool use, and Response Inhibition. Some of the additional tasks were only administered with the chimpanzees at Sweetwaters Chimpanzee Sanctuary due to time constraints. These tasks were administered as a second task following an EF task. Chimpanzees were individually tested but were separated from the others for no longer than 30 minutes. There was only one test session per task, day, and individual. The chimpanzees were fed multiple times a day with fresh fruits and vegetables and the research did not interfere with the feeding schedules.

Table S4. Overview of the nine EF and additional tasks for chimpanzees in their chronological order over the 18 testing days.

| **Day** | **Task order** | **Session description** | **Max duration** | **Additional tasks** |
| --- | --- | --- | --- | --- |
| 1 | WM Updating (part 1)^1^ | Warm-up: single platform, 4 / 5 boxes (3 trials each) | 30 min |  |
| 2 | WM Updating (part 2)^1^ | Test (4 trials) | 30 min |  |
| 3 | Shifting Tray (part 1)^2^  Inhibition Cylinder (part 1)^3^ | Test (24 trials)  Test (1 session) | 20 min  10 min |  |
| 4 | Shifting Tray (part 2)^2^  Inhibition Cylinder (part 2)^3^ | Test (24 trials)  Test (1 session) | 20 min  10 min |  |
| 5 | Inhibition Grid (part 1)^4^ | Test (6 trials) | 10 min | Overimitation^5^ |
| 6 | Inhibition Grid (part 2)^4^  WM Boxes^1^ | Test (6 trials)  Warm-up (2 trials) and test (8 trials) | 10 min  20 min | Associative Tool Use^5^ |
| 7 | WM Grid^4^ | Warm-up (2 trials) and test (24 trials) | 30 min |  |
| 8 | Shifting Boxes (part 1)^1^ | SD (max 72 trials) | 30 min |  |
| 9 | Shifting Boxes (part 2)^1^ | SR (max 24 trials) | 30 min |  |
| 10 | Shifting Boxes (part 3)^1^ | CD (24 trials) | 30 min |  |
| 11 | Shifting Boxes (part 4)^1^ | ID (24 trials) | 30 min |  |
| 12 | Shifting Boxes (part 5)^1^ | ED 1 (20 trials) | 30 min |  |
| 13 | Shifting Boxes (part 6)^1^ | ED 2 (20 trials) | 30 min | Overimitation^5^ |
| 14 | Inhibition Boxes (part 1)^1^ | Test (12 trials) | 10 min | Stop signal task |
| 15 | Inhibition Boxes (part 2)^1^ | Test (12 trials) | 10 min | Stop signal task |
| 16 | Shifting Shelf (part 1)^4^ | Training 1 (max. 72 trials) | 30 min |  |
| 17 | Shifting Shelf (part 2)^4^ | Training 2 (max. 72 trials) | 30 min |  |
| 18 | Shifting Shelf (part 3)^4^ | Test (36 trials) | 30 min |  |

Notes: ^1^Reward: 1/16 piece of apple (amount depended on number of correct trials), ^2^Reward: 1/16 piece of apple (amount depended on number of correct trials), ^3^Reward: peanuts (12 per session). ^4^Reward: banana pieces (amount depended on number of correct trials). ^5^Sweetwaters (Kenya) sample only.

Table S5. Overview of the nine EF tasks, task goals, dependent variables (DVs), procedure, and inclusion criteria for both species.

| **Task** | **Goal** | **Signature limit** | **DV** | | **Reason for selection of DV** | **Procedure** | | **Inclusion criteria** | |
| --- | --- | --- | --- | --- | --- | --- | --- | --- | --- |
|  |  |  | **Chimpanzees** | **Children** |  | **Chimpanzees** | **Children** | **Chimpanzees** | **Children** |
| Inhibition Boxes | Inhibit reaching towards box showing attractive but inaccessible reward, instead reach towards opaque box containing hidden reward | Prepotent bias to reach towards box with attractive reward in trial 1 | Proportion of correct searches out of all administered searches in session 1 (max 12 searches) | As in chimpanzees | Performance in session 1 (chimpanzees) and in trial 1 (children) shows signature of prepotent response | 2 sessions with 12 trials each | 1 session with 12 trials | 50%: 6 or more choices (per session) | 75%: 9 or more searches |
| Inhibition Cylinder | Inhibit reaching towards cylinders showing attractive reward but reveal unattractive item, instead reach towards cylinders showing unattractive item but reveal reward | Prepotent bias to reach towards cylinder displaying attractive reward in trial 1 (chimpanzees) or warm-up (children) | Proportion of correct searches out of first 12 unique searches (i.e., not counting repeated searches) in session 1 | As in chimpanzees | Performance in session 1 (chimpanzees) and warm-up trials (children) shows signature of prepotent response | 2 sessions: no warm-up, unlimited number of searches (max 10 min) | 1 session: 1 warm-up trial, 1 test trial (unlimited number of searches; max 10 min) | >50%: 7 or more choices (per session) | 75%: 9 or more searches |
| Inhibition Grid | Inhibit reaching towards transparent doors showing inaccessible reward, instead reach towards opaque doors that open | Prepotent bias to reach towards transparent door in trial 1 | Proportion of correct searches out of the first 6 unique (i.e., not repeated) searches in session 1 | As in chimpanzees, trial 1 | Performance in first session (chimpanzees) and in trial 1 (children) shows signature of prepotent response | 2 sessions with 6 trials per session: Unlimited number of searches, max 10 min | 1 session with 2 trials: Unlimited number of searches, max 10 min | 100%: 6 unique searches | 100%: 6 unique searches |
| Shifting Boxes | Shift attention away from irrelevant dimension of the complex stimulus | Children: better performance in Simple Discrimination task, in which only one dimension is present | Proportion of correct searches out of all administered searches in the CD phase (**max 72 trials**) | As in chimpanzees, but **max 24 trials** | Large individual variation and above chance performance in contrast to ED phase for chimpanzees | **6-7 sessions.** Criterion in SD/CD/ID (max 3 sessions or 72 trials to reach the criterion): criterion 6 in a row correct. SR: max. 24 trials or 6 consecutive trials correct | **2 sessions:** Session 1: SD and Reversal. Session 2: CD and ED.  Max 24 trials (in 2 blocks of 12) in SD/Reversal/CD. **Relevant dimension shape**.  Criterion in SD/Reversal/CD: 6 in a row correct or any 9 out of 12 correct in block 2. Stopping rule: criterion reached OR max number of trials reached.  ED: 36 trials. |  | 75%: 18 or more trials |
| Shifting Shelf | Shift attention to box predictive of reward based on location of the shelf presented | Majority of mistakes in test are switching mistakes | Proportion of switches out of all possible switches in the administered test trials (max 12 switches) | As in chimpanzees | Proportion of possible switches more robust than proportion of correct trials in cases in which participants had an incomplete number of test trials. Number of switching mistakes discarded as for some trials the type of mistake was not recorded | **3 sessions**: 2 training sessions, 1 test session. Max 72 trials per training step and max 108 trials to complete training. Training 1 criterion: 3 in a row correct on both platforms. Training 2 criterion: any 6 out of 7 correct (rolling window), with at least 3 trials on each shelf. 36 test trials | **2 sessions**: 1 training session (training 1 and 2a), 1 training (training 2b) + test session. Max 36 trials per training step and max 108 trials to complete training.  Criteria as in chimpanzees, with the exception that in training 2a and 2b  children were not required to have at least 3 trials on each shelf |  | 75%: 27 or more trials |
| Shifting Tray | Shift attention away from dimension boxes, which has been predictive in previous tasks, to less salient dimension filling material | Pilot study with children showed bias towards dimension “boxes” if children had previous experience with tasks in which boxes were used as hiding places | Proportion of correct searches out of all administered searches in **session 2 (max 24 trials)** | As in chimpanzees, but **session1 1, max 36 trials** | Large individual variation and above chance performance in contrast to session 1 performance for chimpanzees | 2 sessions with 24 trials per session | **1 session: max 36 trials** (in 3 blocks of 12). Learning criterion: 6 in a row correct or any 9 out of 12 correct in blocks 2 and 3. Stopping rule: criterion reached OR max number of trials reached. |  | Reached criterion or max number of trials |
| WM Boxes | Remember location of reward on platform 1 while resisting interference from information about location of second reward on platform 2 | Better performance in warm-up without second platform compared to test | Proportion of correct searches out of all administered test searches (**max 24 searches**) | As in chimpanzees, but **max 8 searches** | Captures test performance best | 1 session: 2 warm-up trials (each containing 2 searches: platform 1 and 2), 12 test trials (each containing 2 searches) 🡪 **24 test searches** | As in chimpanzees, but only 4 test trials 🡪 **8 test searches** |  | 75%: 3 or more test trials |
| WM Grid | Remember location of reward in a grid while resisting interference from visual tracking distractor task | Better performance in warm-up without distractor task compared to test | Proximity of first search to the baited compartment (range 0-1), averaged across all administered test trials (max 12 searches) | As in chimpanzees | Captures test performance best | 1 session: 2 warm-up trials, 12 test trials | As in chimpanzees |  | 75%: 9 or more test trials |
| WM Updating | Remember and update which boxes have been emptied on platform 1 while resisting interference by information on which boxes have been emptied on platform 2 | Better performance in warm-up without second platform compared to test | Proportion of correct choices across both platforms (searches 3-8, i.e., 6 searches), **averaged across 4 trials (24 searches total)** | Proportion of correct choices across both platforms (searches 3-8, i.e., 6 searches), **in trial 1 (6 searches total)** | Captures test performance best (first 2 searches of the trial excluded as those cannot be wrong, to ensure a possible performance range of 0-100%) | **2 sessions: 1 training session, 1 test session.**  Test session: **4 trials (each containing 8 searches** across platforms) 🡪 **32 searches**  Stopping rule: if one platform empty, only 1 more search on other platform OR all rewards found OR **max 6 mistakes** | **1 session: 1 test session.**  Test session: **2 trials (each containing max 18 searches** across platforms) 🡪 **max 32 searches**  Stopping rule: if one platform empty, only 1 more search on other platform OR all rewards found OR **max number of searches reached** |  | 100%: 8 searches in trial 1 |
|  |  |  |  |  |  |  |  |  |  |
|  |  |  |  |  |  |  |  |  |  |
|  |  |  |  |  |  |  |  |  |  |
|  |  |  |  |  |  |  |  |  |  |
|  |  |  |  |  |  |  |  |  |  |

Note. Differences in procedure between species are highlighted in bold.

## Procedure

### **Children**

Children were tested individually in their nursery/school by a member of our child research team. All members happened to be female. In many, but not all, sessions a second researcher or a (male or female) research assistant was present to assist during data collection. Depending on the facilities, testing took place either at a child-sized table, an adult-sized table, or on the floor (except from the Cylinder task, which was always administered on the floor). In total, seven researchers conducted testing. A child was tested either by a single researcher throughout the test battery or by up to three different researchers, depending on availabilities and testing schedules. Adherence to the testing protocols was ensured through training sessions. Children received stickers, sticker scenes, and small toys throughout the study as rewards. Each testing session was videotaped.

On their first testing day, children who were signed up for the study were approached by a member of nursery staff who introduced the researcher(s) to the child. The child was then invited to play a game. Children who were shy or hesitant were invited to have a look at the game first and could then decide if they wanted to play. If needed, a member of staff accompanied the child for the first (few) testing session(s) until the child felt comfortable to go with the researcher on their own. Children who did not want to play the game or interact with the researcher were approached again in a friendly manner on each of the next few testing days. Some children then decided to take part (after they had seen other children win stickers and enjoy themselves), but some children never joined the experimenter for games. Once the testing in the nursery had advanced to about halfway through the test battery for most children, the experimenter stopped asking any children who had not started the test battery by then.

Children who decided to participate were greeted by the researcher(s) and invited to the research area to play a fun game in which they could win stickers. To raise children’s interest, colourful sticker scenes were laid out on the table or floor and children were invited to pick the one they liked best. They could then collect stickers for the scene in the first game. If children were happy to have a look at the game, the experimenter invited them to sit down on the chair or on a cushion on the floor. The experimenter then explained to the child that they could stop the game at any time:

“Before we start, I would like you to know that you can stop the game at any time! If you don’t want to play with me anymore, or you don’t like the game, you just tell me and you can go back to nursery. Should we practise that? So what could you tell me if you want to stop the game?”

If children did not answer or replied “I don’t know”, the experimenter suggested a few things that they could say, e.g., “I want to stop” or a thumbs-down sign. These instructions were repeated on the following few testing sessions if deemed necessary, and reminders were given throughout the course of the study. Then the experimenter explained the game (i.e., the Scrambled box task) and asked the child whether they wanted to play that game. This procedure (explaining the game, then explicitly asking whether the child was happy to play the game) was done on each testing day. That way, we could obtain children’s oral assent for each session. In each session, the experimenter was attentive towards signs of distress by the child. If the experimenter got the impression that the child got distressed, sad or fatigued, she asked them whether they wanted to go back to playroom.

Each testing session was recorded on video. Children who expressed concern about the presence of the camera (e.g., some asked whether we would upload the videos to the internet) were explained that the video helped the researcher to remember what they had played together on that day and that no videos would be uploaded to the internet.

Inhibition Boxes

The task was a forced-choice task consisting of twelve trials administered in one session. In each trial, children could choose between an opaque and a transparent box. The transparent box contained a visible, but inaccessible sticker on top of the transparent intermediate ceiling. Children needed to learn to inhibit reaching to the box with the visible (but inaccessible) sticker and instead choose the opaque box in order to retrieve the accessible sticker that was hidden inside the filling material of the opaque box. Before each session, twelve plastic surprise eggs were pre-baited with one sticker each. At the beginning of the test, the experimenter explained the game:

“In this game, you can win some stickers for your sticker album. We are going to play several rounds and in each round, I am going to hide one sticker and you can try to find it. Would you like to have a go at the game? [...] Great!”

The experimenter baited the opaque box behind an occluder by placing one of the surprise eggs into the opaque box, ensuring that the cotton wool fully covered the egg so that the egg was not visible. Then, she removed the occluder and placed the boxes forward (while looking to the floor to avoid any inadvertent cuing) and said:

“Ok, there’s a sticker somewhere here. Can you find it?”

The child was then allowed to make a choice by touching one box. As soon as the child touched a box, the experimenter withdrew the non-chosen box and encouraged the child to remove the filling material from their chosen box. If children chose the correct (opaque box), they were praised and assisted in opening the egg, after which they could stick the sticker into their album. If they chose the wrong (transparent box), the experimenter said something like “Oh no!”/”Nothing there!”/”No sticker this time!” “We’ll try in the next round!”. The experimenter did not reveal the correct location of the sticker. Then the experimenter and/or the child put the cotton wool back into the box, the experimenter took the box back and placed the occluder between them and the child to hide the baiting for the next trial. The location of the transparent and opaque boxes was counterbalanced across trials and they stayed on the same side for a maximum of two consecutive trials. Children received different orders of trials (with respect to the location of the sticker in each trial). Testing ceased once twelve trials were over or the child asked to stop the game.

Inhibition Cylinder

The task consisted of a warm-up and a test trial administered in a single session. In the warm-up, children experienced that when they lifted the cylinders showing an attractive sticker there was a boring dot sticker, and when they lifted the cylinders showing a boring dot sticker there was a nice sticker underneath. Additionally, the experimenter explicitly told the children about the mismatch between the visible and hidden sticker underneath. In the test trial, they needed to inhibit reaching to the cylinders showing the nice stickers and instead choose the cylinders with the boring dot stickers.

At the beginning of the session the child (sitting on the floor) was presented with a small green board on top of which there were two cylinders (a sticker cup and a dot cup). The experimenter explained:

“So in this game you can win some [e.g. dinosaur, Disney Princesses, Paw Patrol] stickers. Before we go to the big board over there to look for some stickers, we’ll start with this small board here. Here you can get your first sticker! Do you want to have a look?”

Children were encouraged to lift a cylinder. If they chose the sticker cup (and found the dot sticker), the experimenter said: “Oh! A boring dot! This is not what we wanted. Shall we try the other cup also?” If the child chose the dot cup (and found the sticker), the experimenter said: “Wow! You found your first sticker! Well done! Shall we try the other cup also?” Thus, children were always encouraged to lift both cylinders. Once both cylinders were lifted, the experimenter said:

“Hm, how funny… underneath the cup with the nice sticker there was this boring dot, but underneath the cup with the boring dot there was your nice sticker!”

The experimenter then removed the small board and the test trial began. Then she placed the large board in front of the child and said:

“Now you can try to find even more stickers for your sticker album on this board. You can put all the stickers that you find here and later you can stick them into your book.”

Then children were encouraged to find as many stickers as possible. Testing ceased once children got all 12 stickers, when 10 min were over or when children announced that they had found enough stickers and they had lifted at least “unique” 12 cylinders (i.e., without counting repeated lifts). Choices were coded from video and recorded as soon as children lifted a cup. Note that this is different to the criterion used in most other tasks (apart from Shifting Shelf) in which a choice is coded as soon as a child touches a box/cup. We scored the responses in this way because children showed frequent hovering and/or brief touching of the cylinders before making an “actual” choice (by lifting the cylinder and checking what is underneath).

Inhibition Grid

The task consisted of two trials administered in a single session. Children needed to learn to inhibit reaching towards transparent doors (showing an animal toy) which turned out to be closed and instead reach to the opaque doors which were able to open to reveal an animal toy.

Before a child arrived at the research area, the experimenter inserted the seven opaque and six transparent boxes into the apparatus in a pseudo-random and prefixed order and baited each box with an animal toy. The exact location of the opaque and transparent boxes was randomized across both trials and all children received the same configurations and the same order of configurations. At the beginning of the testing session, the apparatus was occluded and the experimenter explained to the child:

“In this game, we are going to play two rounds, and in each round you can look for animal toys in a box that is just behind here. I will show it to you in a second. All the animals that you can get out of the box you can keep and take home with you. How does that sound? Would you like to have a go at this game? … Great!”

She removed the occluder and, if needed, pushed the apparatus towards the child, and said:

“Ok, this is the box. Can you get some animals out of there?”

Children were allowed to interact with the box freely and to search for animal toys unrestrictedly (max 10 min). The experimenter gave general encouragement (e.g., “There is a way to get some toys out of there!” / “Keep playing with the box!”). If children had been struggling to figure out how to open the opaque doors for a long time (> 5 min) and looked distressed or unmotivated, the experimenter gave one of the opaque doors a slight push (“Look”). This was the case for a minority of the children (see results). Once the child found all toys, the experimenter praised the child, pulled the apparatus back and occluded it to rearrange the boxes for trial 2. Each child received two trials.

Shifting Boxes

The task consisted of two sessions that were administered on two consecutive testing days. Session 1 consisted of two phases: A Simple Discrimination learning (SD) phase and a Reversal learning (Reversal) phase. Children only moved on to the Reversal if they reached the learning criterion in SD (see below). Session 2 consisted of two phases: A Compound Discrimination learning (CD) phase and an Extradimensional shift (EDS) phase. Children only moved on to the EDS if they reached the learning criterion in CD.

Each phase consisted of a forced-choice task in which children were presented with two boxes. The game was to find a sticker which was in one of the boxes. In the SD phase, the boxes differed only in shape, while their filling material was the same. Children needed to learn that the sticker was always in the box with the golden circles. In Reversal, the rule was reversed (but this was unannounced) and the stimulus predictive of the reward was the box with the blue waves. In the CD phase, the boxes presented different in both shape and filling material. Children needed to learn that the sticker was always hidden in the box with the silver stripes. In SD, Reversal, and CD children’s attention had to focus on the shape of the box while the filling material had to be ignored. In EDS, children were again presented with boxes that differed in shape and filling material. This time, the stimulus predictive of the reward was the green felt filling material. In order to find as many stickers as possible, children needed to shift their attention across dimensions from box shape to filling material.

A previous, unpublished, study showed that when the filling material was the predictive dimension in SD, reversal, and CD, children found the switch to the shape of the box in EDS extremely difficult. This might have been exacerbated by the fact that children might have a bias in assuming that one of the materials is predictive as the materials were in direct contact with the reward, had to be removed in order to find the reward and as children had direct haptic experience with the material. Therefore, for the current study, it was decided that the shape of the box should be the relevant dimension in SD, Reversal, and CD, with a shift to filling material in EDS as we were hoping that this would prevent us from getting a floor effect for the EDS stage.

Session 1 started with the SD in which children needed to discriminate boxes based on their shape. They were presented with two boxes (one with golden circles, one with blue waves as decorations), both containing the same filling material. At the beginning of the session, children were presented with the boxes and the experimenter explained the game:

“So in this game you can win some stickers for your sticker sheet! We are going to play several rounds and in each round, I am going to hide one sticker and you can try to find it! You can only choose once in each round. Would you like to play this game? […] Great!”

The experimenter occluded the boxes and placed a sticker inside the box with the golden circles. Then she removed the occluder, placed the boxes closer towards the child and said (while looking to the floor to avoid any inadvertent cuing):

“Ok, so there is a sticker hidden somewhere here. Can you find it?”

The child was allowed to choose by touching one of the boxes. The experimenter then encouraged the child to extract the filling material from the box. If the child chose the correct box, they were praised and allowed to stick their sticker onto their sheet. If they chose the wrong box, the experimenter said “Nevermind, we can try again in the next round!”. She put the filling material back into the box and occluded the boxes for the next trial. One box was baited per trial. The feature predictive of the sticker was the golden circles. The left/right position of the baited box was pseudo-randomized across trials with the constraint that it appeared an equal number of times on the left and right within a block of 12 trials. All children were presented with the same order of trials. The maximum number of trials was 24, arranged in 2 blocks of 12 trials. The SD stopped when children reached the learning criterion (6 trials in a row correct or any 9 out of 12 trials in block 2 correct), when the maximum number of trials was reached or if children asked to stop the game.

If, and only if, children met the learning criterion, they immediately progressed to Reversal. Here, the same boxes were used, but this time the feature predictive of the reward were the blue waves. The change in rules was not announced to the children. Again, the maximum number of trials was 24, arranged in 2 blocks of 12 trials. The Reversal stopped when children reached the learning criterion (6 trials in a row correct or any 9 out of 12 trials in block 2 correct), when the maximum number of trials was reached or if children asked to stop the game.

Children were administered Session 2 (on the next day) regardless of whether they reached criterion in Reversal and received the same instruction as Session 1. Again, children were presented with two boxes in each trial, but this time the boxes differed in both shape and material. Boxes had to be discriminated based on their shape, the silver stripes were the feature predictive of the reward. Again, the maximum number of trials was 24, arranged in 2 blocks of 12 trials. The CD stopped when children reached the learning criterion (6 trials in a row correct or any 9 out of 12 trials in block 2 correct), when the maximum number of trials was reached or if children asked to stop the game.

If, and only if, children met the learning criterion, they immediately progressed to EDS in which they needed to discriminate novel boxes that differed in shape and filling material. The predictive stimulus was the green felt filling material and the box shape was now irrelevant. Children were administered a fixed number of 36 trials. The repeated exposure to shape as a reward predictor in the three pre-switch stages (SD, reversal, CD) was aimed to fixate children’s attention to this stimulus dimension.

Shifting Shelf

In this task, children needed to switch attention between the relevant objects for predicting the location of a reward depending on the location of the shelf they were presented with. There were two different shelves with a bottom and a top level that served as contextual cues. On platform 1, children learned one discrimination task (that the sticker was always underneath the green cup, on the bottom), on platform 2 they learned another discrimination (that the sticker was always underneath the pink cup, on the top). In the test, children needed to combine the contextual cue (which platform they were currently presented with) with the object cue (which cup was predictive of the reward) to find the sticker.

The task consisted of three training phases and a test phase, administered in two sessions on two consecutive testing days: Session 1 consisted of Training 1 and 2a. In Training 1 children were familiarised with the shelves and the general nature of the game (that they were alternating between shelves) and learned that on each shelf there was one cup that predicted the location of a sticker (green cup on green shelf, pink cup on blue shelf). In Training 2a children were familiarised with the fact that each shelf could hold four cups. The cups predictive of the reward were the same as in Training 1. Session 2 (on the next day) consisted of Training 2b (the same as Training 2a, serving as a warm-up) and the test.

For Training 1, there were only two cups on each platform (green platform, lower level: orange and green cups; blue platform, upper level: pink and yellow cups). At the beginning of the session, the experimenter explained the game:

“So in this game, you can win some stickers for your sticker sheet, ok? We are going to play several rounds, and in each round you have the chance to win a sticker. How does that sound? Would you like to have a go at the game?”

The experimenter occluded the green shelf and hid a sticker underneath the green cup. Then, she removed the occluder, pushed the green shelf toward the child and said (while looking to the floor to avoid any inadvertent cuing):

“Ok, so there is a sticker hidden somewhere here. Can you find it?”

Children were allowed to choose only one cup in each trial. A choice was scored as soon as children lifted a cup. Note that this criterion is different to most other tasks (apart from Inhibition Cylinder) in which touching a box/cup was sufficient to be counted as a choice. This was because in the Shifting Shelf task children regularly touched one or more cups briefly (or hovered over them) before making their “actual” choice (by lifting a cup). Importantly, however, as soon as children lifted a cup, that cup was recorded as their choice, even if children attempted to pick another cup afterwards. If they chose correctly, they received the sticker, otherwise the experimenter showed the child the location of the sticker but kept the sticker for the next trial. The experimenter occluded the shelf again, rearranged the cups according to a predetermined order and hid a sticker. Then the second trial started. Children searched on the green shelf until they chose correctly in three consecutive trials. Then the experimenter switched to the blue shelf, occluded it, hid a sticker underneath the pink cup, removed the occluder, pushed the shelf towards the child and allowed the child to choose a cup. Children searched on the blue shelf until they chose correctly in three consecutive trials. Training 1 stopped once children reached the learning criterion (three consecutive correct trials on shelf 1 and three consecutive correct trials on shelf 2), or the maximum number of trials (36) or if children asked to stop the game.

If, and only if, children reached the learning criterion, they immediately moved on to Training 2a. At the beginning of this phase, the experimenter added two distractor cups on each shelf: On the green shelf, a blue and a red cup were placed on the upper level; on the blue shelf, a red and a blue cup were placed on the lower level. The distractor cups never served as hiding places for the sticker and were closed at the bottom. Children first searched on the green shelf. When they chose correctly in three consecutive trials, they switched to the other platform. Again, when children chose correctly for three consecutive trials on platform 2, they switched back to platform 1. If children chose correctly, they received the sticker, otherwise the experimenter showed the child the location of the sticker but kept the sticker for the next trial. Children reached the learning criterion in Training 2a if they scored 6 out of any 7 trials correct. Training 2a stopped when children reached the learning criterion or the maximum number of trials (36) or if they asked to stop the game. If children scored the first 6 trials correct, we stopped immediately and did not run a 7th trial. If, and only if, children reached criterion in Training 2a, they qualified for Training 2b in the next session (otherwise testing in this task was abandoned).

Session 2 started with Training 2b, which was the same as Training 2a. Children started on the platform that they did not end with in Training 2a. If, and only if, children reached the learning criterion in Training 2b, they were immediately administered the test.

In the test phase, the blue and red distractor cups on each shelf were replaced by distractor target cups, so that the same four cups were used on both shelves: a green and an orange cup on the bottom, a pink and a yellow cup on the top (see Fig. S6). The combination of location and identity of the cups predicted the presence of the sticker in the same manner as in the training: the green cup on the lower level indicated the location of the sticker on shelf 1 (green) and the pink cup on the upper level indicated the location of the sticker on shelf 2 (blue). Children started the test on the platform that they did not end with in Training 2b. Children were tested on one shelf until they chose correctly for three consecutive trials. Then they continued on the other platform. In the test phase, children did not receive feedback about the location of the sticker if they chose incorrectly. Children completed 36 test trials. The test stopped once children completed 36 trials or if children asked to stop the game. The location of the cups on the platforms was predetermined, in a way that the same cup did not appear on the same location for more than two trials.

Shifting Tray

This game was a forced-choice task administered in a single session. In each session, children were presented with two trays, one filled with red shredded paper, the other one with green sand. Each tray had a box on top of the substrate, with its opening facing downwards, thus serving as a potential hiding place for a sticker. The combination of substrates and boxes as well as the location of the trays was predetermined, so that each substrate/cup appeared the same number of times on each side within a block of 12 trials, but that no substrate/box appeared on the same side more than three times in a row. Children needed to learn that the sticker was always hidden in the red shredded paper and that the dimension box could be ignored. By positioning this task in the test battery after a series of tasks in which children had to pay attention to boxes (Scrambled box task, Working Memory Updating, Shifting Shelf, Inhibition Grid), we were hoping that children’s attention was sufficiently biased towards the dimension box. Thus, in the Shifting Tray task, children should have a tendency to consider one of the boxes as predictive of the reward. In order to find as many stickers as possible, children needed to overcome this tendency and shift their attention to the unusual dimension substrate.

At the beginning of the session, children were presented with the two trays and the experimenter explained the game:

“So in this game you can win lots of stickers for your sticker sheet. We are going to play several rounds. In each round, I’m going to hide one sticker somewhere here [point to trays]. Your task is to find the sticker. In each round, you can only choose once. Would you like to have a go at the game? … Great!”

The experimenter occluded the trays, hid a sticker underneath the cup sitting on top of the shredded paper, removed the occluder and pushed the large tray towards the child. She said (while looking to the floor to avoid any inadvertent cuing):

“There is a sticker somewhere here. Can you find the sticker?”

The child was encouraged to search in one tray and to pick one of the boxes. A choice was recorded as soon as children touched a cup. If the child chose the correct box, they were praised and allowed to place the sticker onto their sheet. If the child chose the wrong tray, the experimenter said “Oh no, not there! Let’s try again in the next round!” The location of the sticker was not revealed to the child. The experimenter then pulled the tray back, occluded it, rearranged the location of the trays and boxes according to a predetermined order and hid a sticker for the next round.

Children received one session consisting of three blocks of 12 trials each. Every block included all combinations of filling materials and boxes. Each substrate/cup appeared the same number of times on each side within a block of 12 trials, but no substrate/box appeared on the same side more than three times in a row. We used the same order of trials across children. The task stopped when children reached the learning criterion (6 trials in a row correct or any 9 out of 12 trials in blocks 2 or 3 correct), when the maximum number of trials (36) was reached or if the child asked to stop the game.

Working Memory Boxes

The task consisted of two warm-up trials (with two searches in total) and four test trials (with 8 searches in total) administered in a single session. In the warm-up, children were presented with one platform with four identical boxes and the task was for children to remember one sticker location over a short retention interval (15 sec). In the test, there were two identical platforms and children needed to remember two sticker locations (one location on each platform) over a short retention interval (15 sec) in which the platforms were occluded.

At the beginning of the warm-up, children were presented with one platform with four identical cardboard boxes on top. Each box was filled with white tissue paper that prevented children from seeing the inside of the box. The experimenter explained the game:

“In this game, you can win even more stickers for your sticker album! We will start this game very easy. I’m going to put a sticker somewhere here, but you need to wait until you are allowed to take it, ok?”

The experimenter showed the child a sticker, put it into one of the boxes via the hole in the back and said: “Now we need to wait.” Following a retention interval (15 seconds), the experimenter pushed the platform forward (while looking to the floor to avoid any inadvertent cuing) and allowed the child to choose one of the boxes by touching a box. Then the child was encouraged to remove the tissue paper from the chosen box. If the child chose the correct box, they were praised. If the child chose an incorrect box, the experimenter said: “Oh no, the sticker was not there!” and removed the sticker from its actual location. Children were only allowed to search once. Children received two warm-up trials.

For the test, the experimenter added a second platform with another four identical boxes to the setup and said:

“This time you can get two stickers. I’m going to put one sticker here [touch platform 1] and the other one is going in here [touch platform 2] but again you need to wait until you are allowed to take the stickers!”

The experimenter showed the child a sticker and hid it in a box on platform 1. Following a short delay (5 sec), she occluded platform 1. The experimenter then showed a second sticker and hid it in a box on platform 2 (in second 7). Following a short delay (5 sec), she moved the occluder from platform 1 to platform 2. In second 15, the experimenter pushed platform 1 towards the child (while looking to the floor to avoid any inadvertent cuing) and said “Now you can look for the sticker on this side.” The experimenter allowed the child to choose one of the boxes by picking up a box. The child removed the tissue paper from the chosen box. If the child chose the correct box, they were praised. If the child chose an incorrect box, the experimenter said: “Oh no, the sticker was not there!” Then the experimenter removed the occluder from platform 2 and children were allowed to choose a box from platform 2. After the end of a trial, children were allowed to stick on any stickers they won. Children received only one choice per platform in each trial and four test trials in total. Within this session, every box on the platform was baited once. We never baited the same box on both platforms (in terms of their relative spatial position on each platform). All children received the same order of trials.

Working Memory Grid

The task consisted of two warm-up trials and twelve test trials administered in a single session. In the warm-up trials, children watched an animal toy being hidden in one of the boxes in the grid and needed to remember its location over a short retention interval (15 sec). In the test trials, the task was the same, but during the retention trial the box was occluded and children had to solve a distractor task. In order to find as many toys as possible, children needed to remember their locations and resist interference from information encoded during the distractor task.

In the warm-up, children were presented with the grid containing 16 identical cardboard boxes and a platform with three identical, green boxes on top. At the beginning of the session, the experimenter explained the game:

“In this game, you can win some animal toys as well as stickers. We are going to play several rounds and in each round, I am going to hide one animal somewhere here. After I hide an animal, you will need to wait for a little bit, and when I say “go” then you look for it. How does that sound? Would you like to have a go at this game? [...] Ok, so let’s do a practice round first! I will put an animal into this box.”

The experimenter held up an animal toy, removed one of the boxes from the grid by pulling it out from the back of the apparatus, placed the toy inside the box in full view of the child and placed the box back into the grid. She said:

“Then we will need to wait just a little bit [15 sec retention interval; the experimenter was writing some notes on the coding sheet and did not react to the child]. Ok, can you find the animal?”

The child was then allowed to choose one box by touching/pushing it. Once a box was touched/pushed, the experimenter removed it from the grid and handed it over to the child so that they could check whether they found the toy. If the child found the toy, the child was praised and allowed to put the toy into a small bag. If the child did not find the toy, the experimenter put the box back into the grid and allowed the child to search again. Children were allowed to search for the toy up to three times. If the child could not find the toy within three searches, the experimenter removed the toy from the grid. After the first warm-up trial, the experimenter said:

“Well done! Let’s do another practice round! I will put another animal into this box.”, and the second warm-up trial began. After the end of the second warm-up trial, the experimenter said:

“Ok, let’s do the real game now! I will put an animal into this box.”

The experimenter held up an animal, removed a box from the grid, put the toy inside the box and placed the box back into the grid. She said:

“While we wait, why don’t we play another game [put occluder in front of grid] in which you can win a sticker over here? Look!”

The experimenter moved to the platform, showed the child a sticker, opened one of the boxes, placed the sticker inside that box, and closed the box. Then she swapped the location of the box with the location of one of the other boxes in a predetermined order. Then she encouraged the child to find the sticker (while looking to the floor to avoid any inadvertent cuing). After 15 sec, the experimenter moved back to the grid, removed the occluder and encouraged the child to look for the toy. Children received 12 test trials. In each test trial, children could search for the toy up to three times. Testing ceased once the 12 test trials were completed or if the child asked to stop the game. All compartments except for the four edge compartments served as hiding places. The order of baiting of the remaining 12 compartments was randomized. All children received the same order of baiting locations across trials.

Working Memory Updating

In order to prevent children solving the WM Updating task using familiarity with the boxes (i.e., it is easier to remember which boxes one has not emptied yet if one has never seen these boxes before), we used the Scrambled Box task (Diamond, 1997; 8 boxes, 2 trials) as a warm-up task. This task was administered on the previous testing day as the very first task of the test battery.

In the Scrambled Box task, children were presented with one cardboard platform that had eight opaque boxes on top (green, blue, purple, yellow (the same boxes as those used in the WM Updating task) as well as red, orange, light blue, and white). After the general introduction to the games, the experimenter explained the task:

“So in this game I’m going to hide one sticker in each of these boxes. We are going to play several rounds and in each round, you can pick one box. The game for you is to try to win all of the stickers. Would you like to have a go at the game? … Great! Let’s see how many stickers you can find!”

Children watched the experimenter bait the boxes with one sticker per box. After the experimenter closed all the boxes, trial 1 started. The experimenter pushed the platform towards the child, and encouraged them to choose their first box (while looking at the floor to avoid any inadvertent cuing). A choice was coded as soon as children touched a box. The child picked one box, retrieved the sticker and placed it on their sticker sheet. The experimenter closed the box and placed the now empty box back on the platform in its previous location. She occluded the platform to hide the following scrambling event. Boxes were scrambled according to a pre-defined order. After a retention interval of approximately 10 sec, the experimenter removed the occluder and the child could choose again.

In case the child revisited a box, the experimenter opened the indicated empty box, shows its content and said “Oh no!”, “Empty!” or “No sticker this time”, closed the box, and placed it back in its previous position. If the child chose correctly, the experimenter praised the child and the child could place the sticker. Then the experimenter occluded the platform, scrambled the boxes, and the next round started.

This procedure was repeated until the child had retrieved all eight stickers or when the maximum number of rounds (11) was reached. After the last choice in trial 1, the experimenter praised the child, opened and re-arranged the boxes, and discarded any stickers that had not been obtained. Then the child was invited to play a second round and trial 2 started (identical to trial 1). All children received two trials.

On the next day, children continued with the WM Updating task. For this, children were presented with two platforms which each had a set of four different opaque boxes on top (blue, purple, yellow, green). The boxes on platform 1 and 2 were the same. Children watched all the boxes being baited with a reward (one sticker per box). The goal was to collect as many stickers as possible. For this, children were presented alternatingly with platforms 1 and 2 and each time they could choose one box. After children had chosen a box from one platform, the boxes were scrambled behind an occluder. In order to find as many stickers as possible, children had to update the information about which boxes they had already emptied on which platform and resist interference from competing information from both platforms.

At the beginning of the session, the experimenter explained the game:

“So in this game I’m going to hide one sticker in each of these boxes. We are going to play several rounds and in each round, you can pick one box. The game for you is to try to win all of the stickers. Would you like to have a go at the game? … Great! Let’s see how many stickers you can find!”

The experimenter put one sticker in each of the boxes and closed them. Then, she pushed platform 1 (the right platform from the view of the child) towards the child (while looking to the floor to avoid any inadvertent cuing) and encouraged them to pick a box. Once the child touched a box, the experimenter withdrew the platform to discourage the child from picking another box. The child retrieved the sticker and placed it on a sticker sheet. The experimenter closed the box, put it back on the platform and occluded the platform. Then, she pushed platform 2 towards the child. While the child was choosing a box, the experimenter scrambled the boxes on platform 1 in a pre-determined, random order. Once the child had retrieved a sticker, the experimenter closed the box and put in back on platform 2 and then moved the occluder from platform 1 to platform 2. Then the child was allowed to choose another box from platform 1, while the experimenter scrambled the boxes on platform 2. To retrieve all of the stickers as efficiently as possible children need to remember their previous choices (and avoid them). Testing ceased once the child had found all 8 stickers, once they found all 4 stickers on one platform and had been given one last try on the other platform (this was to avoid visits to a platform where all boxes had already been emptied) or after a maximum of 18 searches (i.e., 9 trials). At the end of round 1, the experimenter praised the child, opened and re-arranged all boxes on the platforms, and discarded any remaining stickers into the sticker pile in full view of the child. Then children were invited to play a second round.

### **Chimpanzees**

Inhibition Boxes

Subjects could choose between an opaque and a transparent box. The transparent box contained a visible piece of food (half an apple) on top of the transparent intermediate ceiling (i.e., this food item was inaccessible). Subjects needed to learn to choose the opaque box in order to retrieve the accessible piece of food inside the filling material of the opaque box.

At the beginning of each trial, the experimenter baited both boxes behind an occluder. The lower compartment of both boxes was filled with toilet paper. Subsequently, the experimenter placed a food item inside the paper in the opaque box. After the baiting, the experimenter removed the occluder and pushed the sliding platform forward and the subject was allowed to make a choice. The subject could now remove the paper and the food from the chosen box (if the correct box was chosen). Subjects completed 24 trials within two sessions (12 trials per session). If subjects chose incorrectly the experimenter would not reveal the location of the food reward in the opaque box. The location of the transparent and opaque boxes was counterbalanced across trials and they stayed on the same side for a maximum of three consecutive trials. All subjects received the same order of trials (with respect to the location of the food in each trial).

Inhibition Cylinder

At the beginning of a session, the subject entered the room with the array of 24 cylinders. Subjects could then interact with the cylinders and retrieve the food for a maximum of 10 minutes. If subjects lifted at least 12 cylinders the session was stopped after 5 minutes or when the subject did not lift a cylinder for 30 sec (after they had initiated the search) or earlier if a subject threw the cylinders around without looking for the food (e.g. during dominance display). At the end of a session, subjects were moved to the adjacent compartment.

Subjects completed two sessions in total and one session per day. If an individual threw the cylinders around the session was repeated on the next day. The cylinders were distributed pseudo-randomly with the restriction that two opaque and two transparent cylinders were in each row of the search array. Every individual received the same distribution pattern of cylinders.

Inhibition Grid

At the beginning of each session, the experimenter allocated seven opaque and six transparent flap-doors behind an occluder onto the apparatus (the position of the flap-doors varied in a pseudo-random and prefixed order). The compartments behind the flap-doors were baited with a piece of banana (approximately a quarter banana). After the baiting, the experimenter pushed the sliding platform forward and subjects were allowed to search for food unrestrictedly (max 10 minutes) by pushing against the flap doors. Once an individual stopped interacting with the apparatus for 1 min the experimenter pulled the apparatus back and terminated the session.

Subjects completed two sessions with one trial each. In each quadrant of the grid, there was at least one transparent and one opaque flap door. The exact location of the opaque and transparent flap doors was be randomized across trials. All subjects received the same configurations and the same order of configurations.

Shifting Boxes

At the beginning of each trial, the experimenter baited the boxes behind an occluder and placed them at pre-determined positions on the sliding platform. Then the experimenter pushed the platform forward and the subject was allowed to choose by pointing or touching one of the boxes. the experimenter then pulled the platform back and allowed the subject to extract the filling material from the indicated box.

One box was baited per trial with either the identity of the material or the identity of the box indicating the presence of a reward. A pilot study including 8 chimpanzees (6 females, 2 males) at Budongo Trail, Edinburgh Zoo, revealed that subjects required a similar amount of trials to discriminate between both the identity of the boxes and the identity of the materials. For the current study, filling material was the first relevant dimension and predicted the reward in all test phases except for the last extradimensional shift phase. The left/right position of the baited box was pseudo-randomized across trials with the constraint that it appeared an equal number of times on the left and right within a block of four trials. All subjects were presented with the same order of trials.

Subjects were presented with a series of discrimination tasks (max 30 minutes per session). In all discriminations, we used a criterion of 6 consecutive trials correct for subjects to pass this discrimination phase except for the final extradimensional shift in which we use a fixed number of trials. Subjects needed to discriminate boxes based on their filling material, first in a simple discrimination (SD; test to criterion: 6 consecutive trials correct or 9 out of 12 in trial 13 to 24 of the first session; toilet paper vs brown paper; toilet paper is the baited stimulus; max 3 sessions or 72 trials to reach the criterion) and a reversal of the SD (SR, max. 24 trials or 6 consecutive trials correct; brown paper is baited) followed by a compound discrimination (CD; 1 session; sawdust vs cotton with sawdust as baited stimulus) with novel stimuli. In the CD stage and all subsequent stages, we used distinct boxes; however, the filling material continued to be the relevant dimension (except for the ED stage). After the CD phase, subjects received an intradimensional shift, a novel compound discrimination of new materials (ID; leaves vs mud; leaves baited). Finally, they received the extradimensional compound discrimination (ED, 2 session with 40 trials in total; cork box baited; distractor: palm fibre vs straw) in which they needed to discriminate novel boxes (the filling material was now irrelevant).

The repeated exposure to material as a reward predictor in the four pre-switch stages (SD, SR, CD, ID) aimed at fixating subjects’ attention to this stimulus dimension. Subjects who passed the criterion of 6 consecutive trials correct continued with the next stage right away.

Shifting Shelf

In this experiment, chimpanzees needed to switch flexibly between two object discrimination tasks. There were two different shelves that served as different contextual cues. On platform 1, chimpanzees learned a discrimination between two visually distinct boxes, on platform 2 they learned another discrimination between two different boxes. In the test, chimpanzees needed to combine the contextual cue (which platform) with the object cue (which box) to find the food reward.

There were two training steps: in the first training step, there were only two cups on each platform (Platform 1, lower level: A and B; Platform 2, upper level: X and Y). In this training step, subjects were trained that one of the cups (B; chrome cup; see Fig. S17) on the lower shelf of sliding platform 1 and another cup (X; brown box) on the upper shelf of sliding platform 2 predicted the presence of food rewards. Subjects first searched on platform 1 until they chose correctly in three consecutive trials. Then they searched on platform 2 until they chose correctly in three consecutive trials.

In the second training step, we added two distractor boxes on each platform. We added two distractor boxes on the upper shelf of platform 1 and the lower shelf of platform 2. These distractor cups (D1 / D2) never served as a hiding place for the food. Subject searched first on the platform they did not end on in training phase 1. They searched on this platform until they chose correctly in three consecutive trials and then on the other platform until they chose correctly for three consecutive trials. Subjects passed the second training step if they scored 6 out of 7 trials in a row correct (with at least three correct trials on both platforms).

In each trial, the food was hidden out of view and then subjects could search for the food by choosing one of the four cups on each shelf. If they chose correctly, they received the food item; otherwise the location of the food reward would be revealed but the food item would be discarded. Subjects received a maximum of 72 trials per training step and a maximum of 108 trials to complete both training steps.

In the test phase, the same four cups were used on both platforms. We used the training cups from the lower shelf of platform 1 (A and B) and the cups from the upper shelf of platform 2 (X and Y). The combination of location and identity of the cups predicted the presence of the food reward in the same manner as in training: cup B on the lower shelf indicated the location of the food on platform 1 and cup X on the upper shelf indicated the location of food on platform 2. After three consecutive correct choices on one platform subjects switched to the other platform. Subjects started on platform 1. In the test phase, subjects did not receive feedback about the location of the food reward if they chose incorrectly.

Subjects completed one test session with 36 trials. A session consisted of blocks of 4 trials each. Every block included all 4 combinations of how the cups on the upper and lower shelf could be distributed. The order of trials in each block was randomized. We used the same order of trials for all subjects.

Shifting Tray

In the beginning of a trial, the experimenter baited one of the boxes on the platform behind an occluder outside the subject’s view. Then the experimenter removed the occluder and pushed the platform forward. The subject could now make a choice. If they chose the baited cup /tray they received the food reward. If they chose incorrectly the experimenter showed them the content of the chosen (empty) box and then opened the baited box and discarded the food.

Subjects received two sessions of 24 trials each. A session consisted of 6 blocks of 4 trials each. Every block included all 4 combinations of filling materials, boxes, and their location. The order of trials in each block was randomized. We used the same order of trials across subjects.

Working Memory Boxes

The experiment started with a warm-up phase. In the warm-up, there was one platform with four identical, opaque boxes and subjects needed to remember one food location over a short retention interval (15 seconds). At the beginning of each warm-up trial, the experimenter showed the subject a reward (1/16 piece of apple) while calling the subject’s name and tapped the reward two times on top of the baited box. The experimenter then baited one of the boxes via the hole in the backside. Following a retention interval (15 seconds), the experimenter pushed the sliding platform forward and allowed the subject to choose one of the boxes by reaching toward or touching a box. The experimenter allowed the subject to remove the toilet paper from the indicated box. Subjects received only one choice per trial. Subjects received 2 warm-up trials.

In the test phase, there were two platforms with four identical, opaque boxes per platform and subjects needed to remember two food locations over a short retention interval (15 seconds). We never baited the same box on both platforms with respect to their relative spatial position on each platform. At the beginning of each test trial, the experimenter showed the subject a reward while calling the subject’s name. the experimenter then baited a box on platform 1. Following a short delay (5 seconds), the experimenter occluded the box array on platform 1, showed a second reward, again called the subject’s name and moved to platform 2. Another 2 seconds later (in second 7), the experimenter baited a box on platform 2 and occluded the array on platform 2. After another 8 seconds (second 15), the experimenter pushed the sliding platform forward (while looking to the floor to avoid any inadvertent cuing) and allowed the subject to choose one of the boxes by reaching toward and touching a box. The experimenter allowed the subject to remove the toilet paper from the indicated box. Then the subjects were allowed to choose one box from platform 2. Subjects received only one choice per platform in each trial. Subjects received 8 test trials in one session. Within this session, subjects completed 2 blocks of 4 trials. In every block, every box on the platform was baited once. All subjects received the same order of trials.

Working Memory Grid

At the beginning of each warm-up trial, the experimenter called the subject to draw its attention on the flap-door box on top of the sliding platform. The experimenter showed the reward (a quarter banana) to the subject, held up a flap-door and positioned the reward in the respective compartment in full view of the subject. Then, the experimenter releases the flap-door. After the retention interval of 15 sec, the experimenter pushed the apparatus to the subject and the subject could search for the food reward. Subjects received two warm-up trials.

Test trials were identical to warm-up trials with the exception that in the retention interval the experimenter called the subject to the adjacent sliding platform. On platform 2, the experimenter positioned three cups, showed the subject another reward (a peanut) and placed the reward under one of the cups. Then, the experimenter changed the cup position according to a predetermined order (a single transposition) and the subject was allowed to choose one of the cups. After the 15 sec interval, the experimenter pushed the flap-door apparatus to the subject and the subject could search for the food reward.

Subjects completed 12 test trials. All compartments except for the four edge compartments served as a hiding place. The order of baiting of the remaining 12 compartments was randomized. All individuals received the same order of baiting locations across trials.

Working Memory Updating

Subjects watched how a number of opaque boxes were baited (reward: 1/16 piece of apple). Subjects could choose one box after the other with a 10-s retention interval in between each choice (boxes were occluded during the retention interval). To retrieve all of the food items as efficiently as possible subjects needed to remember their previous choices (and avoid them).

In the training, four or five opaque boxes with lids served as hiding places of food rewards. The boxes differed in colour and shape but were similar in size. The location of each box on the platform remained constant across subjects and trials. At the beginning of each trial, the experimenter baited all the opaque boxes on the table in full view of the subject (from left to right). After the baiting, the experimenter showed the content of each box to the subject by tilting it toward the subject right before they placed the lid on top of the box. After the baiting of the boxes, the experimenter pushed the sliding platform forward and allowed the subject to choose one of the boxes by reaching toward and touching a box. The experimenter opened the indicated box and passed the reward to the subject. The experimenter closed the box and placed the now empty box back on the platform in its previous location. The experimenter occluded the platform for 10 seconds by holding an opaque PVC board in between the boxes and the subject, to avoid visual tracking of the containers. the experimenter did not change the locations or the baiting status of the boxes during the delay. the experimenter’s hands were visible to the subject during the entire delay to emphasize that the status of boxes remained unchanged. After the delay, the experimenter removed the occluder and the subject was allowed to choose again. In case the subject revisited a box, the experimenter opened the indicated empty box, showed its content to the subject, closed the box again, and places it back in its previous position. Then the experimenter occluded the boxes again, and the next 10-sec delay period started.

This procedure was repeated until subjects had retrieved all food items or until they made five mistakes within the same trial. After the last choice in a trial, the experimenter opened all boxes on the platform and discarded the remaining food items in a food bucket underneath the platform in full view of the subject. In the training session, all individuals first received three trials with four boxes and then three trials with five boxes.

In the test (second session), we used two adjacent platforms (platform 1 and 2). At the beginning of each trial, the experimenter placed four boxes each on platform 1 and 2. the experimenter then baited and closes the boxes on platform 1 and 2 and placed a free-standing occluder on platform 2. Subjects could now choose a box on platform 1. the experimenter opened the indicated box, passed the food reward from inside the box to the subject, and closed the box again in the same manner as in the training. After the first choice, the experimenter moved the occluder from platform 2 to platform 1 and occluded the boxes on platform 1. Subjects could now choose from platform 2. the experimenter then transferred the occluder from platform 1 to platform 2 and subjects could choose again from platform 1. This procedure was repeated until subjects had retrieved all food items on one platform or until they had made 6 mistakes. In between each choice there was a 10 seconds retention interval. All individuals receive four test trials.

## Scoring and analysis

### **Children**

Inhibition Boxes

A choice was coded as soon as the child touched one of the boxes. We scored:

- Whether the child stopped the game (yes/no)
- For each trial: Box chosen (transparent/opaque)
- For each trial: Side of opaque box (left/right)
- For each trial: Side chosen (left/right)
- Box chosen in trial 1 (transparent/opaque)
  - If box chosen in trial 1 was transparent, how long the run of selecting the transparent box was (including trial 1; 1-12)
- Total number of correct trials (possible range: 0-12)
- Number of errors after the first opaque box was chosen (if an opaque box was ever chosen; 0-11)

The main DV was the proportion of correct trials out of all administered trials. Children had to complete 75% of the trials (i.e., nine or more trials) in order to be included, otherwise they would be considered a dropout.

Inhibition Cylinder

A choice was coded as soon as the child picked up one of the cylinders. We scored:

- Whether the child stopped the game (yes/no)
- For each trial: Cylinder chosen (sticker/dot)
- For each trial: whether the cylinder had been chosen before (yes/no)
- Cylinder chosen first in warm-up (sticker/dot)
- Whether children found all twelve stickers (yes/no)
  - If children did not find all stickers, how many stickers they found (1-11)
- Duration of trial (in sec)
- Total number of searches until all stickers found or test was stopped
- Number of unique (i.e., not repeated) searches (max 12)
- Number of correct searches within the first twelve (or less) unique searches

The main DV was the proportion of correct searches out of the first twelve (or less, but more than 75%, i.e., nine, ten or eleven) unique searches. If children only completed less than nine unique searches, they were considered a dropout.

Inhibition Grid

A choice was coded as soon as the child touched one of the doors. We scored:

- Whether the child stopped the trial (yes/no)
- For each search: which door was touched (transparent/opaque)
- For each search: the position of the touched boxes (by numbering the locations in the grid from 1 to 16, starting from the top left (experimenter’s view))
- For each search: whether the door had been touched before (within the same trial; yes/no))
- If the child touched an opaque door from which they had not yet retrieved the reward: whether they obtained the toy (yes/did not open door/did not fully open door/opened door but did not take animal)
- Door touched in first search (transparent/opaque)
- Number of opaque (correct) boxes touched within the first six unique (i.e., not repeated) searches
- Duration of the trial from first touch of a door (in sec)
- Latency to first reward from first touch of a door (in sec)
- Latency to complete six unique (i.e., non-redundant) searches (in sec)
- Whether all rewards were retrieved (yes/no)
- Total number of searches until all rewards retrieved (in sec)
- Total number of times a transparent door was touched (including repeated touches of the same door)
- Total number of times a transparent door was touched after the first reward was retrieved (including repeated touches of the same door)
- For the first 2 min after the first touch of a door:
  - Number of unique (i.e., not repeated) touches
  - Number of opaque (correct) doors touched within the first six (or fewer if fewer touches occurred within the first 2 min) unique searches

The main DV was the proportion of correct searches within the first six unique searches (i.e., not counting any repeated searches of transparent or opaque doors). If children did not complete six unique searches, they were considered a dropout.

Shifting Boxes

A choice was coded as soon as the child touched a box. In each phase, we scored:

- Whether the child stopped the game (yes/no)
- Total number of administered trials (max 24 in SD, Reversal, CD; max 36 in EDS)
- Number of correct trials
- Learning criterion reached (for SD, Reversal, CD only; yes/no)
  - If criterion reached: Number of trials until criterion reached

The main DV was the proportion of correct trials out of all administered trials in the CD phase. Children had to complete at least 75% of the trials (i.e., 18 trials) in order to be included, otherwise they would be considered a dropout.

Shifting Shelf

A choice was coded as soon as the child picked up one of the boxes. We scored:

- Training 1:
  - Whether the child stopped the game (yes/no)
  - Whether the criterion was reached (yes/no)
  - The number of administered trials (possible range 6-36)
  - The number of errors (0-36)
  - Training 2a and 2b:
  - Whether the child stopped the game (yes/no)
  - The number of shifts
  - Whether the criterion was reached (yes/no)
  - The number of administered trials (possible range 6-36)
  - The number of errors (0-36)
  - Whether children proceeded to session 2 (yes/no)
- Test:
  - Whether the child stopped the game (yes/no)
  - The number of switches (possible range 0-11)
  - The total number of mistakes (0-36)
  - The number of mistakes made directly after a switch (includes trial 1, as this is a switch from training 2b)
  - The number of mistakes in which the correct shelf was chosen, but the wrong cup
  - The number of mistakes in which a cup on the wrong shelf was chosen
  - The number of mistakes in which the wrong shelf was chosen and the cup that was rewarded on the other platform
  - The number of mistakes in which the wrong shelf was chosen and the cup that was never rewarded on the other platform

The DV was the proportion of achieved switches between platforms out of the possible number of switches (11 or 12). Children had to complete at least 75% of the test trials (i.e., 27 trials) in order to be included, otherwise they would be considered a dropout.

Shifting Tray

A choice was coded as soon as the child touched a box. We scored:

- Whether the child stopped the game (yes/no)
- For each trial: Choice (correct/incorrect)
- For each trial: Which distractor box was picked (purple/yellow)
- Total number of trials (max 36)
- Learning criterion reached (yes/no)
  - If the criterion was reached: the number of trials until the criterion was reached
- Number of correct trials

The DV was the proportion of correct trials out of all administered trials. Children had to complete at least 75% of the trials (i.e., 18 trials) in order to be included, otherwise they would be considered a dropout.

Working Memory Boxes

A choice was coded as soon as the child touched a box. We scored:

- Whether the child stopped the game (yes/no)
- For each search: choice (correct/incorrect)
- For each search: location of box chosen on the platform (1, 2, 3, 4)
- For each trial: number of correct searches (0-2)
- Number of correct searches in warm-up (0-2)
- Number of correct searches in the test (0-8)

The DV was the proportion of correct searches out of the eight administered test searches. Children had to complete all eight test searches in order to be included.

Working Memory Grid

A choice was coded as soon as the child touched a box. We scored:

- Whether the child stopped the game (yes/no)
- For each trial: Number of searches until the reward was found (1-3, with 4 indicating “not found)
- For each trial: location of box choices (positions on grid numbered 1-16, from top left to bottom right)
- For the first search in each trial: location of box choice on the grid as x and y coordinates
- For each test trial: reward in distractor task obtained in first search (yes/no)

The main DV was the average distance of the first choice from the hiding location of the reward across all administered test trials. The distance was calculated as follows: $\sqrt{{(Chosen_{X}-Reward_{X})}^{2} x {(Chosen_{y}-Reward_{y})}^{2}}$. The distance could vary between 0 (reward always located in first attempt) to 3.6. We inverted the distance score and transformed it to a range of 0 and 1, so that a number closer to 1 meant a smaller distance to the reward (i.e., better performance). Children had to complete 75% of the trials (i.e., nine or more trials) in order to be included, otherwise they would be considered a dropout.

Working Memory Updating

A choice was coded as soon as the child touched a box. We scored for each trial:

- Whether the child stopped the trial (yes/no)
- Number of searches across both platforms (max 18)
- Whether all boxes were emptied (yes/no)
- Number of boxes emptied (max 8)
- Efficiency ($\frac{Number of boxes emptied}{Number of searches}$)
- Whether the child used a “by colour” search strategy (e.g. yellow-yellow, purple-purple,green-green, blue-blue; yes/no)

For the main DV, we only considered the first eight searches across both platforms in order to establish comparability with the chimpanzee testing procedure. We deselected the first two searches as those are by definition always correct – by deselecting those searches, we were able to calculate a proportion score that could theoretically have a value of 0. The main DV was the proportion of correct searches in trial 1. Children had to complete the first eight searches in trial 1 in order to be included.

### **Chimpanzees**

Inhibition Boxes

A choice was coded as soon as the subject touched or reached toward one of the boxes. We scored whether subjects selected (i.e., point toward or touch) the correct (opaque) or incorrect (transparent) box. The main DV was the proportion of correct trials out of all administered trials.

Inhibition Cylinder

We scored the order of the cylinders lifted by the subject (we used lifting instead of touching of the cylinders here because pilot work had shown that the chimpanzees sometimes touched the cylinders when walking through the array of cylinders without further interacting with them), whether the cylinders were baited and whether the subject had lifted the same cylinder before within the same session. Cylinders that were thrown around and not lifted or turned over one by one were not considered.

The main DV was the proportion of correct searches out of the first twelve unique searches (or less if they stopped their search early; minimum 5 unique choices). One subject (Edvard) was excluded because he did not eat the peanuts. Another individual (Jane) was excluded because she found a transparent closed cylinder with a peanut inside before the first session.

Inhibition Grid

We scored the order of the touched flap doors, whether the flap door was opaque or transparent, and whether the subject had touched the same door before within the same session. The main DV was the proportion of correct searches within the first six unique searches (i.e., not counting any repeated searches of transparent or opaque doors).

Shifting Boxes

A choice was coded as soon as the subject touched or pointed toward one of the boxes. We scored whether subjects selected the correct (baited) or the incorrect (unbaited) box. The main DV was the proportion of correct trials out of all administered trials in the CD phase.

Shifting Shelf

A choice was coded as soon as the subject touched one of the boxes. We scored whether subjects selected the correct (baited) or an incorrect (unbaited) cup. Moreover, we scored switching mistakes, i.e. whether they chose the box that would have been the correct choice on the other platform. The DV was the proportion of achieved switches between platforms out of the possible number of switches (11).

Shifting Tray

A choice was scored as soon as the subject touched or pointed toward one of the boxes or trays. We scored whether subjects select the correct (baited) or the incorrect (unbaited) tray. The DV was the proportion of correct trials out of all administered trials.

Working Memory Boxes

We scored the number of mistakes per trial (0 – 2). The DV was the mean proportion of correct searches out of the eight administered test trials.

Working Memory Grid

We scored the distance of the first chosen cell from the actual hiding location of the food reward. The distance was calculated as follows: $\sqrt{{(Chosen_{X}-Reward_{X})}^{2} x {(Chosen_{y}-Reward_{y})}^{2}}$. The distance could vary between 0 (reward always located in first attempt) to 3.6. We inverted the distance score and transformed it to a range of 0 and 1, so that a number closer to 1 meant a smaller distance to the reward (i.e., better performance).

Working Memory Updating

We only scored the first eight searches per trial across both platforms either as correct (baited) or incorrect (empty). We discounted the first two searches (i.e., the first choices on each platform) as those were by definition always correct. The main DV was the mean proportion of correct searches in choices three to eight.

Table S6. Interobserver reliability of the chimpanzee data for all nine tasks.

| **Task** | **Kappa** | **N** | **p** |
| --- | --- | --- | --- |
| **WM Updating** | 0.976 | 263 | <0.001 |
| **WM Boxes** | 1 | 216 | <0.001 |
| **WM Grid** | 0.919 | 168 | <0.001 |
| **Shifting Tray** | 0.989 | 528 | <0.001 |
| **Shifting Shelf** | 0.989 | 396 | <0.001 |
| **Shifting Boxes** | 0.975 | 1373 | <0.001 |
| **Inhibition Boxes** | 0.946 | 264 | <0.001 |
|  | r_s_ | N | p |
| **Inhibition Grid** | 0.72 | 22 | <0.001 |
| **Inhibition Cylinders** | 0.88 | 22 | <0.001 |

Note: The interobserver reliability was analyzed at the trial level for all tasks in which there was a clear break in between trials. In two tasks (Inhibition Grid and Inhibition Cylinders) there was no clear break or reset of the task situation in between subsequent choices. For these tasks, we analyzed the interobserver reliability at the session level using Spearman correlations.

# **Supplementary Results**

## Individual Tasks

### **Children**


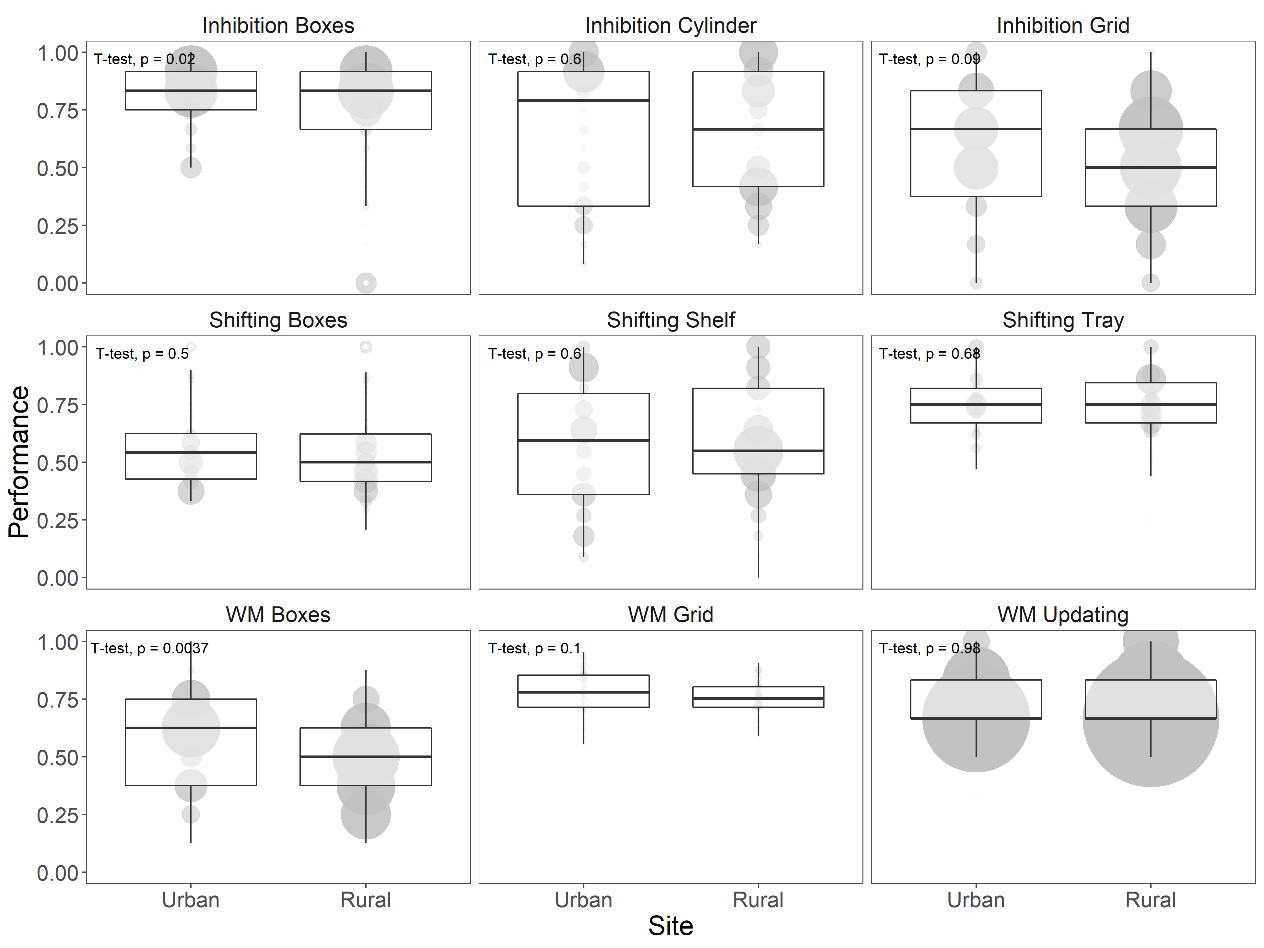


Fig. S22. Boxplots showing the task performance measures (ranging between 0 and 1 with higher values indicating better performance) across the urban and rural areas and all nine tasks for the child sample. The grey dots represent the mean individual performances; the size of the dots is proportional to the number of represented individuals. Independent-samples t-tests are shown for the comparison across sites for each task. Performance in the WM Boxes and Inhibition Boxes tasks differed significantly between sites.


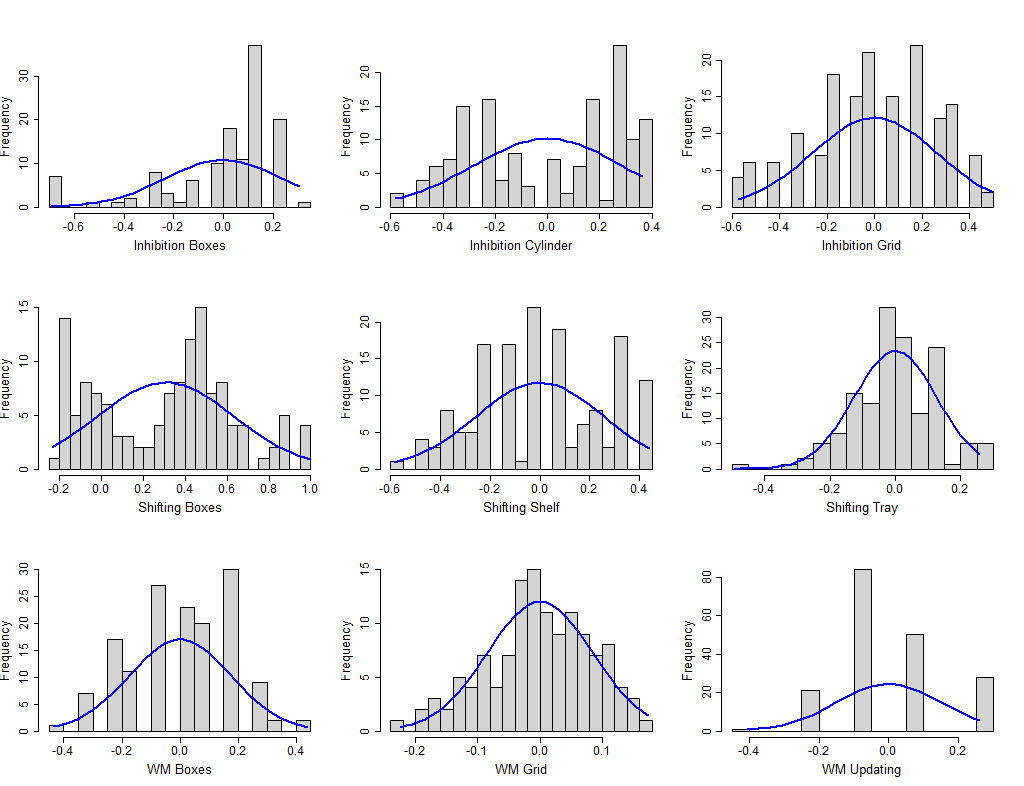


Fig. S23. Histograms for the nine EF tasks for the child sample (centered by site). The blue line represents a normal curve fitted to the data. For sample sizes, see paragraphs below.

Inhibition Boxes

We had valid data for 126 children (67 girls, 59 boys) on this task. Mean age was 49.95 months (sd = 6.94, range 36-72 months). There were 55 3-year-olds, 60 4-year-olds, 10 5-year-olds, and 1 6-year-old. We tested 58 children in the urban and 69 children in the rural area. We tested an additional eight children who were dropped from analysis: five children were excluded due to experimenter error (four children could not see the sticker in the transparent box, one child saw the reward in the bottom part of the opaque box) and three children stopped the game before they reached 75% of the trials (one child after 2 trials, one after 3 trials, one after 7 trials). All children included in the analysis completed twelve trials.

Out of the 126 children, 111 (88%) chose the transparent box in trial 1, demonstrating an initial bias to choose the box with the visible reward (Fig. S24). Out of the 111 children who chose the attractive box in trial 1, the majority (68 children; 61%) chose the opaque box in trial 2. A further 26 children (23%) inhibited reaching towards the transparent box in trials 3 or 4. There were only few children who continued reaching towards the transparent box for more than three trials after the start of the test. Yet, there were seven children who always chose the transparent box. Thus, most children inhibited reaching towards the transparent box relatively early in the test. Disregarding the seven children who never chose the opaque box, we found that about half of the children (n = 59; 49%) never picked the transparent box again after they chose the opaque box for the first time. Another 41 children (34%) only picked the transparent box once after they chose the opaque box for the first time. In total, the mean proportion of correct trials was 0.74 (sd = 0.24, range 0-1). Children performed significantly better than chance level (0.5), two-sided, t-test, t(125) = 11.327, p < .001. Performance of children from the urban area (0.79 ± 0.14, range 0.5-1) was significantly better than performance of children from the smaller towns (0.70 ± 0.29, range 0-1), two-sided t-test: t(101.73) = 2.359, p = .020. We then investigated whether the effect of testing location was due to the greater verbal ability in children from the urban area. For this, we conducted a Generalized Linear Mixed Model on the trial-by-trial data with Success as the dependent variable, and age (z-transformed to a mean of 0 and a sd of 1), the BPVS score and testing location as predictors. While the full model explained the data significantly better than a null model only containing the intercept, χ^2^(4) = 110.75, p < .001, there were no significant effects of testing location (χ^2^(1) = 1.60, p = .206) nor verbal ability (χ^2^(1) = 0.38, p = .535) on children’s success.


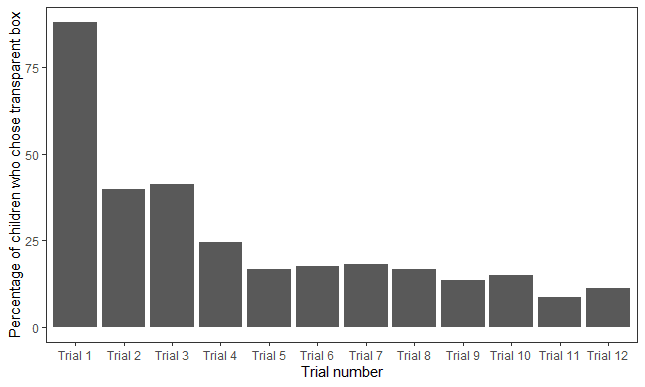


Fig. S24. Percentage of children who chose the transparent box (wrong choice) in each trial in the Inhibition Boxes task.

Inhibition Cylinder

We had valid data for 144 children (78 girls, 66 boys) on this task. Mean age was 49.94 months (sd = 6.81, range 36-72). There were 62 3-year-olds, 70 4-year-olds, 11 5-year-olds, and 1 6-year-old. We tested 60 children in the medium-sized city and 84 children in the smaller towns. We tested an additional three children who were dropped from analysis: two children were excluded due to experimenter error (the experimenter forgot to provide parts of the instruction during the warm-up trial) and one child stopped the game after three searches. All but four children had twelve unique (i.e., not repeated) searches. Two children only had eleven unique searches and two children had only ten unique searches before their test was stopped.

Out of the 144 children, the choice of the first cylinder in the warm-up was known for 130 children (90%). For the rest of the children, the experimenter missed to take a note of the cup chosen and the video also did not show it. Of the 130 children, 122 (94%) chose the sticker cup (i.e., the wrong cup) first in the warm-up, demonstrating that there was a strong initial pull to reach to the cup with the attractive sticker on display. In the first search of the test, 69 of the 144 children (48%) chose the cylinder with the attractive sticker (Fig. S25).

Of the 144 children, 101 (70%) found all twelve stickers. The remaining 43 children stopped the game before finding all the stickers (either they announced they had found all the stickers and did not want to search anymore or the experimenter stopped the game as the child had already searched for a significant amount of time (~ 10 min) and had been unable to find the remaining stickers).

The mean proportion of correct searches within the first 10, 11 or 12 unique searches was 0.65 (sd = 0.28, range 0.08-1). Children performed significantly better than chance level (0.5), two-sided t-test: t(143) = 6.284, p < .001. There was no difference between children from the urban area (0.66 ± 0.30, range 0.08-1) and the smaller towns (0.64 ± 0.27, range 0.17-1), two-sided t-test: t(118.44) = 0.528, p = .598.


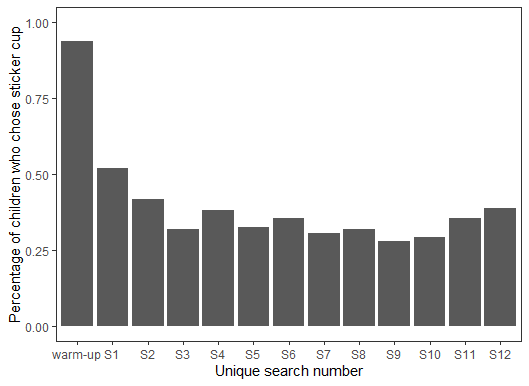


Fig. S25. Percentage of children who chose the sticker cup (wrong choice) in each trial in the Inhibition Cylinder task.

Inhibition Grid

We had valid data for 155 children (79 girls, 76 boys) on this task. Mean age was 49.67 months (sd = 6.94, range 36-72). There were 69 3-year-olds, 75 4-year-olds, 10 5-year-olds, and 1 6-year-old. We tested 65 children in the medium-sized city and 90 children in the smaller towns. We tested an additional eleven children who were dropped from analysis: eight children were excluded due to experimenter error (heavy intervention and prompting (2x), one of the opaque doors was open from the start (2x), the experimenter only put six transparent boxes into the apparatus (2x), camera was positioned badly so that later offline coding was impossible (1x), the experimenter forgot to bait the box (1x), one child stopped reacting early on in the task, one child did not touch the box at all, and one child picked the apparatus up and flipped it towards her so that the rewards fell out).

Out of the 155 children, 127 (82%) touched one of the transparent doors first, demonstrating an initial bias to try one of the doors which showed a reward. The mean proportion of correct searches within the first six unique searches (i.e., not punishing repeated visits to opaque or transparent boxes) was 0.54 (sd = 0.26, range 0-1). Children did not perform significantly better than what would be expected by chance (0.5), two-sided, t-test: t(154) = 1.852, p = .066. There was no difference between children from the urban area (0.57 ± 0.28, range 0-1) and the smaller towns (0.51, sd = 0.24, range 0-1), two-sided t-test: t(128.69) = 1.638, p = .104.

Shifting Boxes

We had valid data for 132 children (70 girls, 62 boys) on the CD phase of the Shifting Boxes task. Mean age was 50.04 months (sd = 6.95, range 36-72). There were 56 3-year-olds, 64 4-year-olds, 11 5-year-olds, and 1 6-year-old. We tested 58 children in the medium-sized city and 74 children in the smaller towns. We tested an additional seven children who were dropped from analysis: one child stopped after trial 1, two children stopped after trial 7, one child after trial 9, one child after trial 14, and one child after 16 trials. For one child, there was an experimenter error in trial 13. The final sample included two children who stopped the test after having completed 75% of the maximum number of trials: One child stopped after 18, and one child after 19 trials. These children were included in the analysis, but note that we could not establish whether these children would have reached the learning criterion.

The mean proportion of correct trials was 0.56 (sd = 0.18, range 0.21-1). Children performed significantly better than what would be expected if they chose randomly (0.5), t(131) = 3.58, p < .001. There was no difference between children from the urban area (0.57 ± 0.18, range 0.33-1) and the smaller towns (0.55 ± = 0.19, range 0.21-1), two-sided t-test: t(125.26) = 0.672, p = .503.

The EDS phase was started by 36 children. One child had to be removed from analysis as they stopped the game after 24 trials (i.e., after less than 75% of the trials). The proportion of correct trials was 0.83 (sd = 0.13, range 0.51-1) and significantly larger than chance level (0.5), t(34) = 15.029, p < .001.

Shifting Shelf

We had valid data for 152 children (84 girls, 68 boys) on the test phase of the Shifting Shelf task. Mean age was 49.91 months (sd = 6.95, range 36-72). There were 68 3-year-olds, 77 4-year-olds, 11 5-year-olds, and 1 6-year-old. We tested 66 children in the medium-sized city and 86 children in the smaller towns. We tested an additional 15 children in the test phase who were later dropped from analysis: for 10 children there was an experimenter error (experimenter showed the correct location of the sticker in every unsuccessful trial (7x), experimenter placed sticker under wrong cup (2x), experimenter made several errors regarding the switch rules (1x)), one child stopped after trial 7, one child stopped after trial 9, one child after trial 13, one child after trial 19, and one child after trial 22. We included ten children who stopped the test before reaching 36 trials but after having completed 75%: 7 children completed 35 valid trials because of an experimenter error in the last trial, 1 child completed 33 trials (experimenter error), 1 child completed 27 trials (experimenter error), and one child stopped after 32 trials.

Training 1 was completed by 176 children and all but one child reached criterion. The mean number of trials to reach criterion was 12.28 (sd = 3.87, range 7-31). Of the 175 children who started Training 2a, 172 reached criterion and needed on average 11.06 trials (sd = 5.27, range 6-34) to do so. Training 2b was started and completed by 166 children who needed on average 8.79 trials (sd = 4.12, range 6-30) to reach criterion. The mean proportion of achieved switches in the test phase was 0.57 (sd = 0.26, range 0-1). Children performed significantly better than chance level (0.225; based on Monte-Carlo 100000 simulations assuming random sampling of two cups only, two-sided t-test: t(151) = 16.755, p < .001). There was no difference between children from the urban area (0.56 ± 0.27, range 0.09-1) and the smaller towns (0.58 ±0.25, range 0-1, two-sided t-test, t(131.50) = -0.526, p = .600).

Shifting Tray

We had valid data for 148 children (77 girls, 71 boys) on this task. Mean age was 49.68 months (sd = 6.56, range 36-65). There were 65 3-year-olds, 72 4-year-olds, and 11 5-year-olds. We tested 61 children in the medium-sized city and 87 children in the smaller towns. We tested an additional three children who were dropped from analysis due to experimenter error: in two cases, the sticker was accidentally placed into the wrong tray in one trial of the test, and in one case the sticker was missing from the correct tray and the experimenter did not reward the child.

Of the 148 children, 139 (94%) reached the learning criterion. The mean proportion of correct trials was 0.74 (sd = 0.13, range 0.25-1). Children performed significantly better than what would be expected by chance (0.5), t(147) = 23.53, p < .001. There was no difference between children from the urban area (0.75 ± 0.12, range 0.47-1) and the smaller towns (0.74 ± 0.13, range 0.25-1).

Working Memory Boxes

We had valid data from 148 children (79 girls, 69 boys) on this task. Mean age was 49.92 months (sd = 6.81, range 36-72). There were 64 3-year-olds, 72 4-year-olds, 11 5-year-olds, and 1 6-year-old. We tested 60 children in the medium-sized city and 88 children in the smaller towns. We tested one additional child who stopped after two test trials (i.e., four searches) and was thus dropped from analysis. All included children completed four test trials (i.e., eight searches).

In the warm-up, 105 children (71%) had both searches correct, 42 (28%) had one search correct and one child had no warm-up trial correct. Regardless of warm-up performance, children proceeded to the test phase. In the test phase, the mean proportion of correct searches was 0.51 (sd = 0.18, range 0.12-1). Children performed significantly better than chance level (0.25), two-sided t-test, t(147) = 17.667, p < .001. There was an effect of testing location in that children from the urban area (mean ± sd: 0.57 ± 0.18, range 0.12-1) outperformed children from the smaller towns (0.47 ± 0.17, range 0.12-0.87), two-sided t-test, t(118.05) = 3.148, p = .002. We then investigated whether the effect of testing location was due to the greater verbal ability in children from the urban area. For this, we conducted a linear regression with the proportion of correct searches in the test trials as the dependent variable, and age (z-transformed to a mean of 0 and a sd of 1), the BPVS score and testing location as predictors. Together, the three predictors explained the data better than a null model only containing the intercept, χ^2^(3) = 0.665, p < .001. There was a significant effect of age (χ^2^(1) = 0.293, p = .001) and testing location (χ^2^(1) = 0.300, p = .001), but no effect of the BPVS score (χ^2^(1) = 0.011, p = .522), suggesting that the better performance of children in the urban area in the Working Memory Boxes task could not be explained with their better verbal ability.

Working Memory Grid

We had valid data from 127 children (70 girls, 57 boys) on this task. Mean age was 49.90 months (sd = 6.86, range 36-72). There were 55 3-year-olds, 61 4-year-olds, 10 5-year-olds, and 1 6-year-old. We tested 58 children in the medium-sized city and 69 children in the smaller towns. We tested an additional three children who were later dropped from analysis: two children stopped after five test trials, one child stopped in the middle of the 8th test trial. All but two children completed all twelve test trials. One child stopped after nine, and one child after ten test trials. As both completed more than 75% of the test trials (i.e., more than nine trials), these children were included in the analysis.

Averaged across the two warm-up trials, mean proximity to the reward was 0.94 (sd = 0.09, range 0.56-1). The Monte-Carlo simulation to determine the chance level was based on 1000000 iterations of 12 random choices of cells on the 4x4 grid. The distance between these random choices and the hiding location of the reward was then calculated. The mean distance from the reward of the random choices was calculated (mean distance 1.95) and rescaled to values between 1 and 0 (with 1 indicating a direct hit of the baited door and 0 indicating the maximum distance from the baited door; rescaled mean proximity: 0.46). Children’s performance in the warm-up trials was significantly better than chance (0.46), t(126) = 57.547, p < .001.

In 83% of the test trials, children found the sticker in the distractor task in their first search, indicating that we were successful in directing children’s attention away from the main task. The mean proximity to the reward averaged across test trials was 0.77 (sd = 0.08, range 0.55-0.95) and also significantly better than chance level, t(126) = 40.391, p < .001. There was no difference in performance in the test trials between children from the urban area (mean ± sd: 0.78 ± 0.09, range 0.55-0.95) and from the smaller towns (0.75 ± 0.08, range 0.59-0.91), two-sided t-test, t(109.5) = -1.66, p = .100.

To examine whether children’s performance would also deviate from a random inner-cell sampling strategy we conducted another Monte-Carlo simulation. The simulation was identical to the aforementioned one but only the inner cells were sampled from resulting in a smaller average distance from the food reward (mean distance 1.67; rescaled mean proximity: 0.54). Children’s performance in the test trials was also significantly better than the simulation based on an inner-cell preference, t(126) = 29.834, p < .001.

Working Memory Updating

We had valid data from 184 children (100 girls, 84 boys) on this task. Mean age was 49.54 months (sd = 6.99, range 36-72). There were 84 3-year-olds, 88 4-year-olds, 11 5-year-olds, and 1 6-year-old. We tested 75 children in the medium-sized city and 109 children in the smaller towns.

We only used trial 1 to create our dependent variable. This was for two reasons. First, only 179 children performed both trials, and in order to include as many children as possible, we chose to focus on trial 1, which was completed by all 184 children. Second, we found that the mean efficiency (i.e., number of emptied boxes out of all opened boxes) in trial 1 (mean ± sd: 0.74 ± 0.15, range 0.33-1) was not different from the efficiency in trial 2 (0.72 ± 0.17, range 0-1), two-sided paired t-test, t(178) = 1.384, p = .168. In addition, there was only a low correlation between the efficiencies in trial 1 and 2 (r = .23, p = .001), suggesting that some children got better and some got worse between trials. We tested one additional child who stopped the test before they completed eight searches in either trial. All included children completed eight searches in trial 1.

For both the child and the chimpanzee sample, we disregarded the first two searches when creating the dependent variable. This was because the first search on each platform was by definition successful (as all boxes were baited) and thus if using searches 1-8 it would not be possible to reach a proportion score of 0. In order to create a DV where any “proportion correct” score between 0 and 1 was possible, we based the calculation of the proportion of correct searches on searches 3 to 8, leaving out the first search on each platform.

The mean proportion of correct searches was 0.74 (sd = 0.15, range 0.33-1). Children performed significantly better than chance level (0.58), two-sided t-test, t(183) = 14.602, p < .001. There was no difference between children from the urban area (mean ± sd: 0.74 ± 0.14, range 0.33-1) and from the smaller towns (0.74 ± 0.16, range 0.50-1).

### **Chimpanzees**


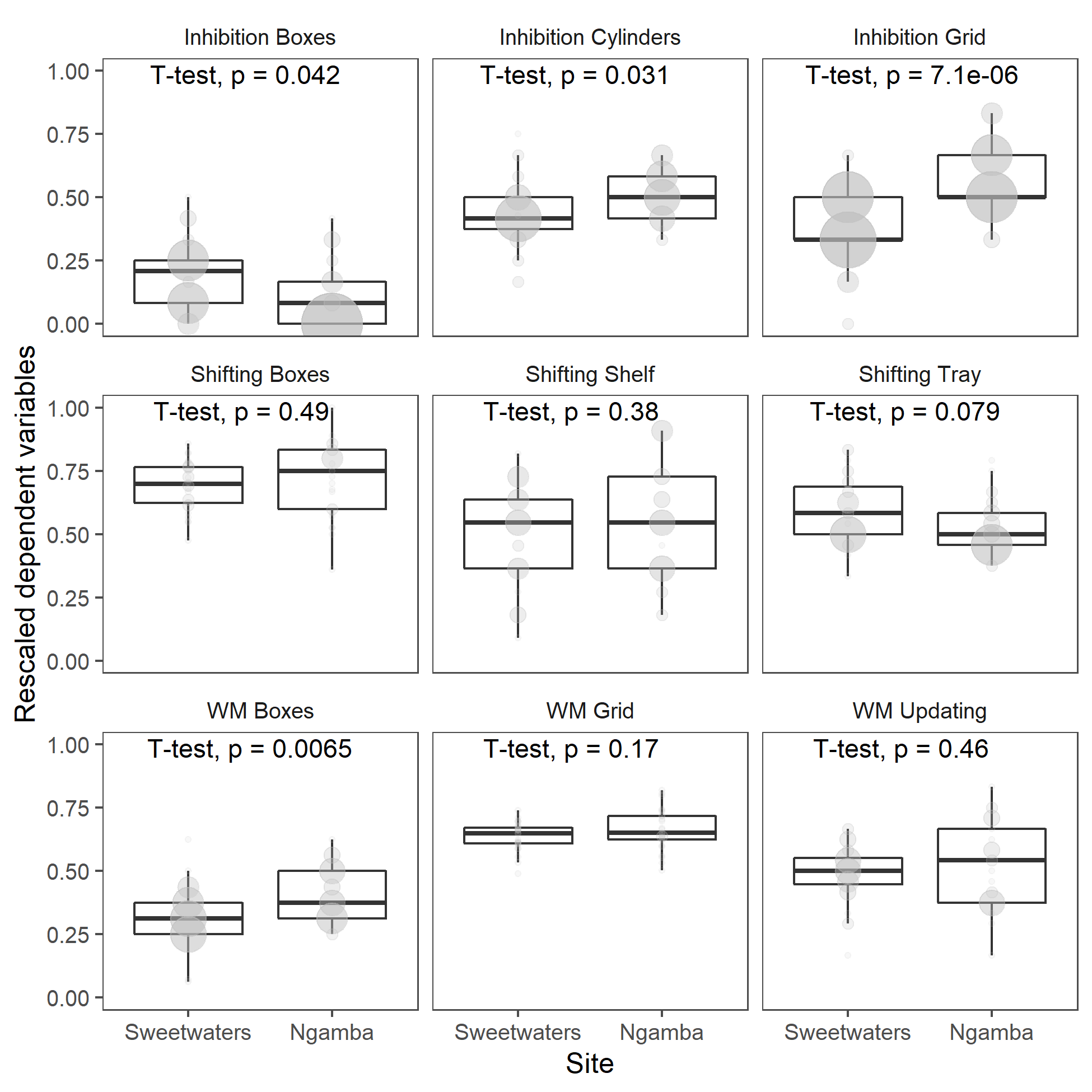


Fig. S26. Boxplots showing the rescaled task performance measures (ranging between 0 and 1 with higher values indicating better performance) across the two sites (Sweetwaters Chimpanzee Sanctuary, Kenya, N = 30; Ngamba Island Chimpanzee Sanctuary, Uganda, N = 25) and all nine tasks. The grey dots represent the mean individual performances; the size of the dots is proportional to the number of represented individuals. Independent-samples t-tests are shown for the comparison across sites for each task.


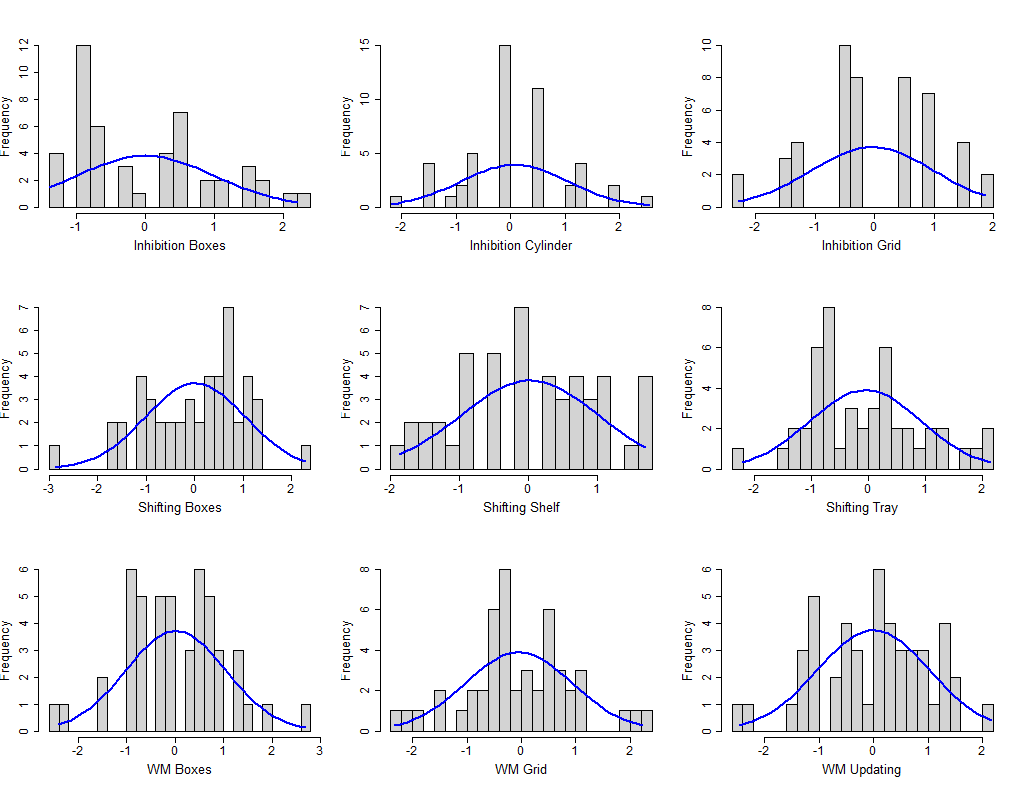


Fig. S27. Histograms for the nine EF tasks for the chimpanzee sample (centered by site; N = 55). The blue line represents a normal curve fitted to the data.

Inhibition Boxes

We had valid data from 53 chimpanzees on this task. The chimpanzees performed significantly below chance in both sessions (session 1: mean proportion correct ± sd: 0.16 ± 0.14; one-sample t-test: t(52)= -17.36, p < .001; session 2: 0.26 ± 0.17; t(52) = -10.43, p < .001). Their performance significantly improved across sessions (t(52) = -4.54, p < .001) and their performance was significantly correlated across sessions (r(51) = 0.43, p = .001).


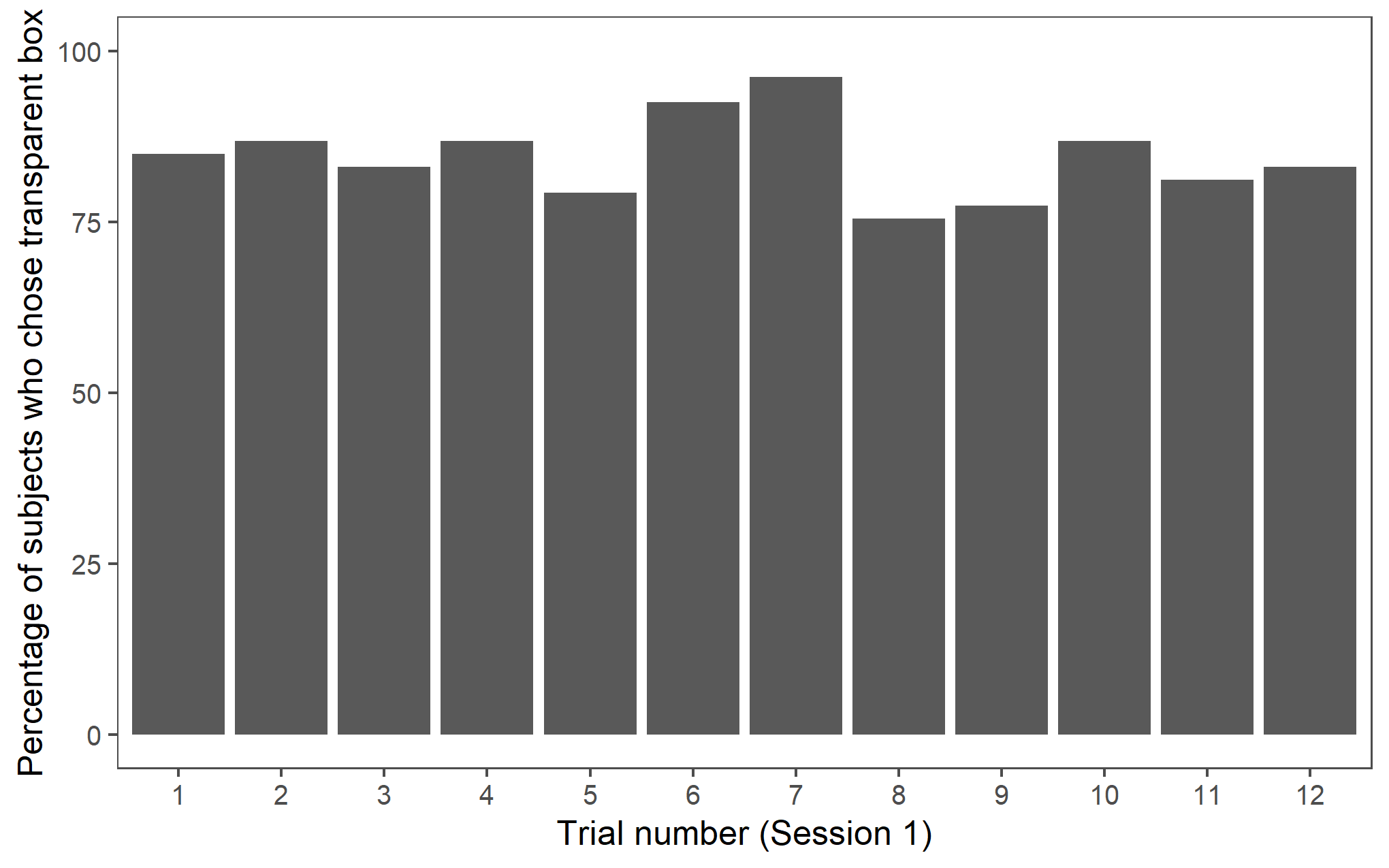


Fig. S28. Percentage of chimpanzees who chose the transparent box (wrong choice) in each trial of session 1 in the Inhibition Boxes task.

Inhibition Cylinder

We had valid data from 52 chimpanzees on this task. The chimpanzees (who did not receive a warm-up) exhibited a significant preference for a transparent cylinder in their first choice (80.8% of the chimpanzees chose a transparent cylinder; binomial test: p < .001) but not in their first 12 unique choices in session 1 (0.47 ± 0.13; t(51) = -1.56, p = .126). In session 2, the chimpanzees had a significant preference for the opaque cylinders (0.67 ± 0.19; t(51) = 6.55, p < .001). Their performance significantly improved across sessions (t(51) = 8.39, p < .001) and it was significantly correlated across sessions (r(50) = 0.47, p < .001).


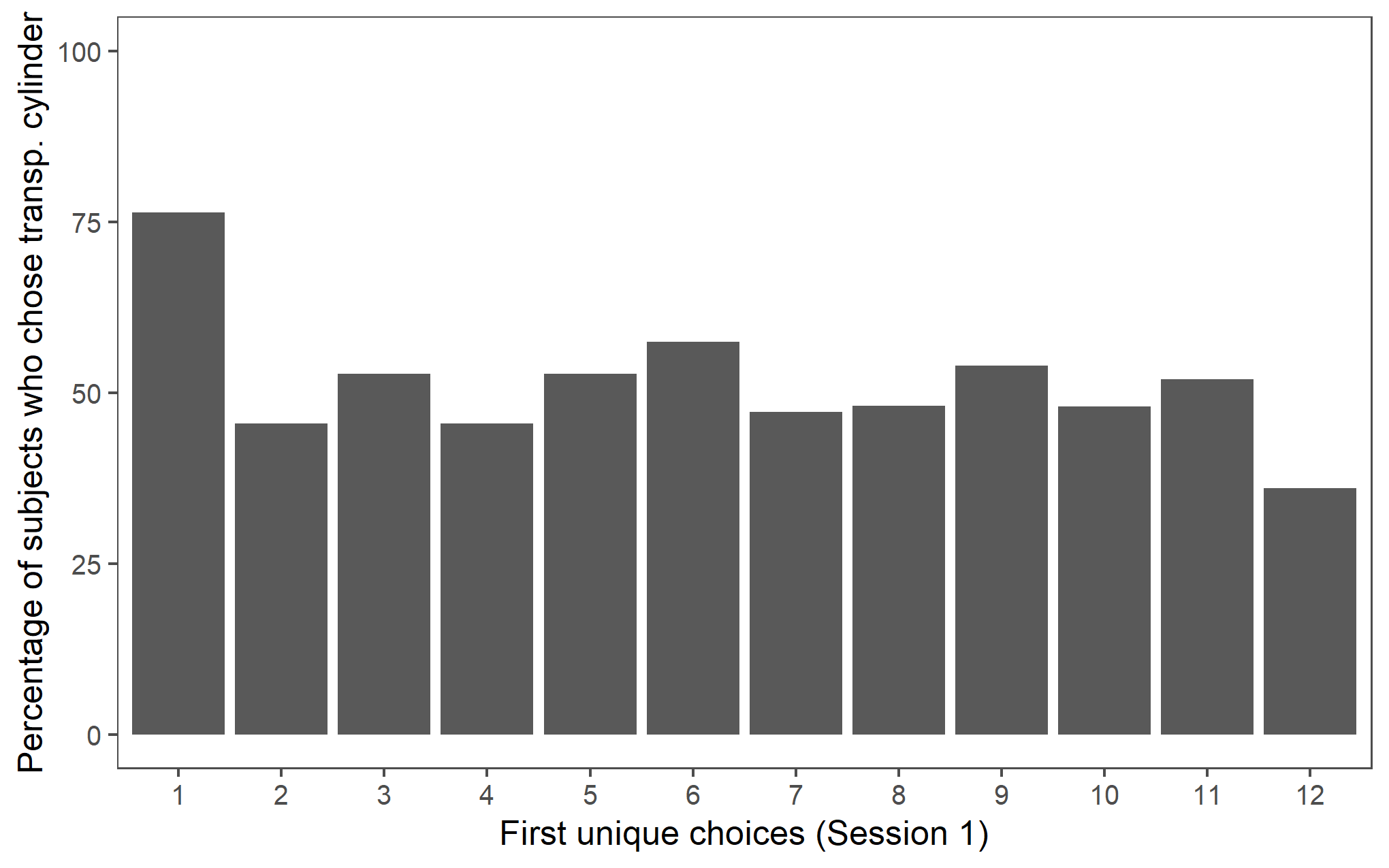


Fig. S29. Percentage of chimpanzees who chose the transparent cylinder (wrong choice) in the first 12 unique choices of session 1 in the Inhibition Cylinder task.

Inhibition Grid

We had valid data from 54 chimpanzees on this task. The chimpanzees exhibited a significant preference for a transparent door in their first choice (64.8% of the chimpanzees chose a transparent door; binomial test: p = .006) and their first six unique choices in session 1 (0.47 ± 0.19; t(53) = -2.62, p = .011) and session 2 (0.47 ± 0.18; t(53) = -2.89, p = .006). Their performance did not significantly change across sessions (t(53) = 0.010, p = .992) and it was significantly correlated across sessions (r(52) = 0.28, p = .040).


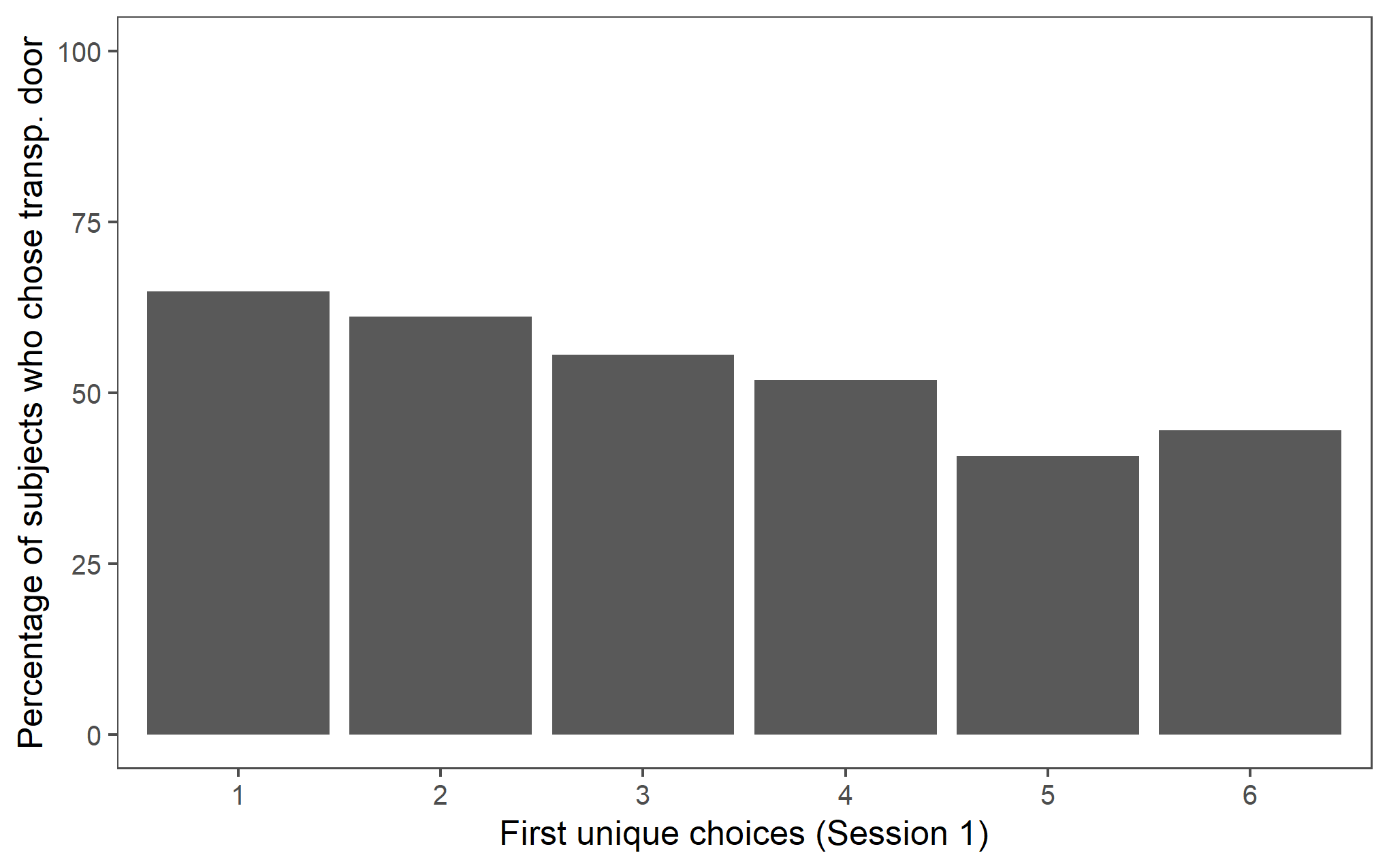


Fig. S30. Percentage of chimpanzees who chose the transparent door (wrong choice) in the first six unique choices of session 1 in the Inhibition Grid task.

Shifting Boxes

We had valid data from 53 chimpanzees on this task. In the SD, CD, and ID phases, the chimpanzees performed significantly above chance (SD: t(52) = 8.31, p < .001; CD: t(52) = 11.87, p < .001; IDS: t(52) = 29.69, p < .001). In the EDS phase, none of the chimpanzees reached the criterion and their performance did not differ significantly from chance level (mean correct ± SE: 0.50 ± 0.05; t(52) = 0.07, p = .948). In the Simple Reversal (SR) phase, chimpanzees performed significantly worse than chance (mean correct ± SE: 0.22 ± 0.21; t(51) = -9.98, p < .001).

Shifting Shelf

We had valid data from 50 chimpanzees on this task. The chimpanzees required on average 24.6 (sd = 12.9; range: 9-57 trials) trials to pass training phase 1 and 17.7 (sd = 9.3; range: 6-53 trials) trials to pass training phase 2 with the added distractor boxes. The chimpanzees scored on average 12.60 (sd = 5.44) trials correct in the first training phase and 11.38 (sd = 4.26) trials correct in the second training phase before reaching the training criterion.

The chimpanzees performed worse in the test phase (mean correct ± sd: 0.62 ± 0.17) compared to the second training phase (0.70 ± 0.13). Nevertheless, the chimpanzees performed significantly above chance level (0.225; based on Monte-Carlo 100000 simulations assuming random sampling of two cups only) with respect to the proportion of platform switches (mean ± sd: 0.52 ± 0.22; t(49) = 9.66, p < .001).

Shifting Tray

We had valid data from 52 chimpanzees on this task. The chimpanzees did not deviate significantly from the hypothetical chance level in the first session (mean ± sd: 0.50 ± 0.09; t(53) = 0.26, p = .793) but they performed above chance in session 2 (0.57 ± 0.12; t(51) = 3.97, p < .001). Their performance was not significantly correlated across the two sessions (r(50) = 0.08, p = .558).

Working Memory Boxes

We had valid data from 53 chimpanzees on this task. The chimpanzees’ mean proportion correct in the warm-up was 0.60 (sd: 0.30, range 0-1), which was significantly better than chance (chance level: 0.25; t(52) = 8.59, p < .001) and thus better than their performance on platform 1 in the test (mean proportion ± sd: 0.38 ± 0.15, range: 0.13-0.75), which was still significantly above chance (t(52) = 6.56, p < .001). Also the chimpanzees overall test performance (considering both platforms) was significantly better than expected by chance (mean number of mistakes per trial: 1.26 ± 0.24; chance level: 1.5; t(52) = -7.32, p < .001).

Working Memory Grid

We had valid data from 53 chimpanzees on this task. The Monte-Carlo simulation to determine the chance level was based on 1000000 iterations of 12 random choices of cells on the 4x4 grid. The distance between these random choices and the hiding location of the food reward was then calculated. The mean distance from the food reward of the random choices was calculated (mean distance 1.95) and rescaled to values between 1 and 0 (with 1 indicating a direct hit of the baited door and 0 indicating the maximum distance from the baited door; rescaled mean proximity: 0.46).

In both phases, warm-up and test phase, the chimpanzees chose doors in their first attempt that were closer to the reward than expected by chance (rescaled chance value: 0.46; warm-up: mean rescaled proximity ± sd: 0.63 ± 0.17; t(52) = 7.55, p < .001; test phase: 0.65 ± 0.07; t(52) = 19.64, p < .001).

To examine whether the chimpanzees’ performance would also deviate from a random inner-cell sampling strategy we conducted another Monte-Carlo simulation. The simulation was identical to the aforementioned one but only the inner cells were sampled from resulting in a smaller average distance from the food reward (mean distance 1.67; rescaled mean proximity: 0.54). The chimpanzees performed also significantly better than the simulation based on an inner-cell preference (0.65 ± 0.07; t(52) = 11.48, p < .001).

Working Memory Updating

We had valid data from 53 chimpanzees on this task. The Monte-Carlo simulation to determine the chance level was based on 100000 iterations of random choices of 4 or 5 cups. We determined the mean number of unique choices per iteration for 4 (2.73) and 5 cups (3.37) and rescaled the value to values between 0 and 1 (with higher values indicating better performance; 4 boxes: 0.58; 5: boxes: 0.59).

The chimpanzees’ performance in the training session with only one platform did not deviate significantly from the hypothetical chance level (4-boxes: mean efficiency ± sd: 0.59 ± 0.16; t(52) = 0.32, p = .747; 5-boxes: 0.62 ± 0.15; t(52)= 1.13, p = .263). However, in the test with two platforms, the chimpanzees performed significantly worse than chance (mean efficiency: 0.51 ± 0.14; t(52) = 3.67, p < .001).

## Confirmatory Factor Analysis

### **Children**

Table S7. Correlations (and sample sizes) of the EF tasks, the BRIEF-P questionnaire measures, and verbal ability (BPVS) for all children (all variables mean centered by site).

|  | Inhibition Boxes | Inhibition Cylinder | Inhibition Grid | Shifting Boxes | Shifting Shelf | Shifting Tray | WM Boxes | WM Grid | WM Updating |
| --- | --- | --- | --- | --- | --- | --- | --- | --- | --- |
| Inhibition Boxes |  |  |  |  |  |  |  |  |  |
| Inhibition Cylinder | 0.11  (124) |  |  |  |  |  |  |  |  |
| Inhibition Grid | 0.07  (120) | 0.10  (137) |  |  |  |  |  |  |  |
| Shifting Boxes | 0.01  (120) | 0.10  (130) | -0.07 (126) |  |  |  |  |  |  |
| Shifting Shelf | 0.18  (108) | 0.25** (125) | -0.20* (139) | 0.08 (117) |  |  |  |  |  |
| Shifting Tray | 0.07  (123) | 0.15  (141) | -0.14 (142) | 0.09 (129) | 0.30*** (128) |  |  |  |  |
| WM Boxes | 0.06  (126) | 0.09  (144) | 0.04  (142) | 0.06 (132) | 0.11 (129) | 0.11 (146) |  |  |  |
| WM Grid | 0.05  (121) | 0.24** (125) | 0.13  (120) | 0.03 (123) | 0.26** (112) | 0.15 (124) | 0.13 (127) |  |  |
| WM Updating | 0.04  (125) | 0.04  (143) | -0.02 (158) | 0.08 (131) | 0.05 (152) | -0.02 (147) | -0.14 (148) | 0.18* (126) |  |
| BPVS | -0.01 (117) | 0.17  (130) | 0.07  (137) | -0.06 (122) | 0.09 (129) | 0.12 (132) | 0.14 (133) | 0.30*** (120) | 0.05  (147) |
| BRIEF-P WM^1^ | 0.05  (92) | -0.08 (106) | -0.08 (106) | -0.01 (97) | -0.01 (96) | -0.10 (105) | -0.17 (107) | -0.07 (94) | -0.07 (114) |
| BRIEF-P Inhibition^1^ | -0.01  (92) | -0.10 (106) | -0.04 (106) | 0.03 (97) | 0.06 (96) | -0.06 (105) | -0.17 (107) | 0.04 (94) | 0.04  (114) |
| BRIEF-P Shifting^1^ | 0.00  (92) | -0.13 (106) | -0.16 (106) | -0.12 (97) | -0.13 (96) | -0.11 (105) | -0.04 (107) | 0.05 (94) | -0.06 (114) |

Notes. The significant correlations are highlighted, * = p < .05; ** = p < .01; *** = p < .001. ^1^Higher scores on the BRIEF-P indicated poorer EF skills.

#### **Domain-specific models**

Table S8. Model estimates of the children data for all 1-factor domain-specific models and their comparison against their respective 3-factor baseline models.

| **Models** | **WAIC** | **SE** | **WAIC difference** | **SE difference** | **p difference** |
| --- | --- | --- | --- | --- | --- |
| Inhibition model | 1227.30 | 36.74 | -0.30 | 0.47 | 0.523 |
| Shifting model | 1222.00 | 42.26 | -6.60 | 4.32 | 0.126 |
| WM model | 1313.84 | 40.13 | -0.01 | 1.01 | 0.992 |

Table S9. Factor loadings of the children data for the one-factor domain-specific models.

| **Model** | **Task** | **Estimate** | **Post.sd** | **95% credible interval** | |
| --- | --- | --- | --- | --- | --- |
| Inhibition | Inhibition Cylinder | 1.00 |  |  |  |
|  | Inhibition Grid | 1.43 | 7.15 | -14.57 | 16.58 |
|  | Inhibition Boxes | 1.89 | 7.51 | -14.98 | 17.48 |
| Shifting | Shifting Boxes | 1.00 |  |  |  |
|  | Shifting Shelf | 4.61 | 3.64 | 0.93 | 14.28 |
|  | Shifting Tray | 3.83 | 3.63 | 0.67 | 13.76 |
| WM | WM Updating | 1.00 |  |  |  |
|  | WM Boxes | -1.03 | 6.71 | -16.19 | 13.38 |
|  | WM Grid | 2.52 | 6.88 | -12.95 | 16.75 |

#### **Run 1**

Table S10. Model estimates for all models and their comparison against the 9-factor baseline model for run 1.

| **Model** | **WAIC** | **SE** | **WAIC difference** | **SE difference** | **p difference** |
| --- | --- | --- | --- | --- | --- |
| 1 factor | 3750.86 | 113.89 | -12.38 | 5.72 | .030 |
| MF2012 | 3754.28 | 113.83 | -10.68 | 5.45 | .050 |
| 3 factors | 3751.39 | 113.50 | -12.12 | 5.66 | .032 |
| 3 independent factors | 3763.63 | 113.67 | -6.00 | 4.37 | .170 |
| 2 factors 1 | 3754.49 | 113.55 | -10.57 | 5.53 | .056 |
| 2 factors 2 | 3759.21 | 113.98 | -8.21 | 4.77 | .085 |
| 2 factors 3 | 3753.10 | 114.82 | -11.26 | 5.25 | .032 |

Table S11. Factor loadings obtained in run 1.

| **Model** | **Latent factor** | **Task** | **Estimate** | **Post.sd** | **95% credible interval** | |
| --- | --- | --- | --- | --- | --- | --- |
| 1 factor | Common | Inhibition Cylinder | 1.00 |  |  |  |
|  |  | Inhibition Boxes | 2.46 | 4.78 | -8.97 | 10.79 |
|  |  | Inhibition Grid | -2.48 | 4.96 | -10.68 | 9.57 |
|  |  | Shifting Shelf | 8.72 | 13.56 | -23.52 | 26.05 |
|  |  | Shifting Boxes | 3.94 | 6.79 | -12.55 | 14.27 |
|  |  | Shifting Tray | 5.50 | 3.38 | -6.32 | 7.86 |
|  |  | WM Updating | 1.33 | 3.38 | -6.32 | 7.86 |
|  |  | WM Boxes | 2.34 | 4.64 | -8.81 | 10.39 |
|  |  | WM Grid | 4.30 | 7.13 | -13.05 | 14.97 |
| MF2012 | WM | WM Updating | 1.00 |  |  |  |
|  |  | WM Boxes | -1.57 | 7.41 | -17.01 | 15.97 |
|  |  | WM Grid | 1.48 | 6.49 | -12.97 | 15.91 |
|  | Shifting | Shifting Boxes | 1.00 |  |  |  |
|  |  | Shifting Shelf | 1.26 | 7.37 | -15.13 | 16.13 |
|  |  | Shifting Tray | 1.14 | 7.37 | -15.58 | 16.22 |
|  | Common | Inhibition Cylinder | 1.00 |  |  |  |
|  |  | Inhibition Grid | -2.80 | 4.98 | -11.74 | 9.32 |
|  |  | Inhibition Boxes | 3.28 | 5.06 | -8.94 | 12.65 |
|  |  | Shifting Shelf | 10.58 | 10.57 | -18.98 | 25.69 |
|  |  | Shifting Boxes | 4.94 | 6.23 | -11.33 | 15.50 |
|  |  | Shifting Tray | 6.75 | 7.66 | -13.70 | 19.31 |
|  |  | WM Updating | 1.95 | 4.51 | -7.95 | 11.39 |
|  |  | WM Boxes | 2.96 | 4.90 | -8.72 | 12.69 |
|  |  | WM Grid | 5.77 | 6.92 | -12.08 | 18.32 |
| 3 factor | WM | WM Updating | 1.00 |  |  |  |
|  |  | WM Boxes | 1.42 | 4.42 | -9.13 | 10.56 |
|  |  | WM Grid | 4.74 | 7.90 | -15.59 | 18.46 |
|  | Shifting | Shifting Boxes | 1.00 |  |  |  |
|  |  | Shifting Shelf | 4.77 | 3.38 | 1.30 | 14.11 |
|  |  | Shifting Tray | 2.80 | 2.28 | 0.72 | 9.12 |
|  | Inhibition | Inhibition Cylinder | 1.00 |  |  |  |
|  |  | Inhibition Grid | -0.50 | 7.94 | -16.62 | 16.94 |
|  |  | Inhibition Boxes | 1.05 | 4.33 | -8.77 | 10.84 |
| 3 independent factors | WM | WM Updating | 1.00 |  |  |  |
|  |  | WM Boxes | -1.40 | 6.83 | -16.37 | 12.91 |
|  |  | WM Grid | 2.31 | 7.00 | -13.23 | 16.55 |
|  | Shifting | Shifting Boxes | 1.00 |  |  |  |
|  |  | Shifting Shelf | 4.07 | 4.77 | -7.92 | 14.57 |
|  |  | Shifting Tray | 3.33 | 4.68 | -8.24 | 13.99 |
|  | Inhibition | Inhibition Cylinder | 1.00 |  |  |  |
|  |  | Inhibition Grid | 1.65 | 7.27 | -15.00 | 16.78 |
|  |  | Inhibition Boxes | 1.70 | 7.50 | -15.24 | 17.17 |
| 2 factors 1 | WM + Shifting | WM Updating | 1.00 |  |  |  |
|  |  | WM Boxes | 3.98 | 2.86 | -0.32 | 10.78 |
|  |  | WM Grid | 7.03 | 3.58 | 1.72 | 15.57 |
|  |  | Shifting Boxes | 5.70 | 3.24 | 0.92 | 13.36 |
|  |  | Shifting Shelf | 11.94 | 5.16 | 3.90 | 23.41 |
|  |  | Shifting Tray | 8.22 | 3.98 | 2.43 | 17.53 |
|  | Inhibition | Inhibition Cylinder | 1.00 |  |  |  |
|  |  | Inhibition Grid | 1.81 | 7.18 | -14.15 | 17.19 |
|  |  | Inhibition Boxes | 1.93 | 7.31 | -14.59 | 16.96 |
| 2 factors 2 | WM + Inhibition | WM Updating | 1.00 |  |  |  |
|  |  | WM Boxes | 0.82 | 6.89 | -13.90 | 13.98 |
|  |  | WM Grid | 3.13 | 11.40 | -19.16 | 21.97 |
|  |  | Inhibition Grid | 1.20 | 7.20 | -14.22 | 14.42 |
|  |  | Inhibition Cylinder | 2.12 | 10.56 | -19.25 | 19.76 |
|  |  | Inhibition Boxes | 0.79 | 6.37 | -12.88 | 13.49 |
|  | Shifting | Shifting Boxes | 1.00 |  |  |  |
|  |  | Shifting Shelf | 4.62 | 3.78 | 0.88 | 14.70 |
|  |  | Shifting Tray | 3.77 | 3.65 | 0.67 | 13.64 |
| 2 factors 3 | Inhibition + Shifting | Shifting Boxes | 1.00 |  |  |  |
|  |  | Shifting Shelf | 11.48 | 5.42 | 2.54 | 22.74 |
|  |  | Shifting Tray | 6.53 | 3.80 | 1.34 | 15.40 |
|  |  | Inhibition Grid | -3.61 | 2.36 | -8.79 | -0.17 |
|  |  | Inhibition Cylinder | 4.53 | 2.65 | 0.79 | 11.12 |
|  |  | Inhibition Boxes | 3.34 | 2.43 | -0.20 | 8.83 |
|  | WM | WM Updating | 1.00 |  |  |  |
|  |  | WM Boxes | -1.03 | 6.03 | -15.13 | 12.21 |
|  |  | WM Grid | 1.87 | 6.11 | -11.20 | 15.59 |

Table S12. Model comparisons between the 1-factor model and all competing models for run 1.

| **Model** | **WAIC difference** | **SE difference** | **p difference** |
| --- | --- | --- | --- |
| MF2012 | -1.71 | 0.92 | .063 |
| 3 factors | -0.26 | 1.90 | .891 |
| 3 independent factors | -6.38 | 3.78 | .091 |
| 2 factors 1 | -1.81 | 2.52 | .473 |
| 2 factors 2 | -4.17 | 4.16 | .316 |
| 2 factors 3 | -1.12 | 3.97 | .778 |

Table S13. Proportion of variance in performance explained by the shared factor on each of the nine EF tasks for run 1 (median and 95% credible interval).

| **Task** | **Proportion of variance explained (median)** | **95% credible interval** | |
| --- | --- | --- | --- |
| Inhibition Boxes | .034 | .000 | .133 |
| Inhibition Cylinder | .002 | .001 | .009 |
| Inhibition Grid | .037 | .001 | .130 |
| Shifting Boxes | .085 | .011 | .224 |
| Shifting Shelf | .416 | .169 | .798 |
| Shifting Tray | .173 | .051 | .362 |
| WM Boxes | .031 | .001 | .124 |
| WM Grid | .099 | .013 | .255 |
| WM Updating | .010 | .000 | .072 |

Table S14. Variance-covariance matrix for the 1-factor model in run 1.

| Task | Inhibition Boxes | Inhibition Cylinder | Inhibition Grid | Shifting Boxes | Shifting Shelf | Shifting Tray | WM Boxes | WM Grid | WM Updating |
| --- | --- | --- | --- | --- | --- | --- | --- | --- | --- |
| Inhibition Boxes | 1.003 |  |  |  |  |  |  |  |  |
| Inhibition Cylinder | 0.007 | 1.005 |  |  |  |  |  |  |  |
| Inhibition Grid | -0.019 | -0.008 | 0.996 |  |  |  |  |  |  |
| Shifting Boxes | 0.030 | 0.012 | -0.030 | 0.971 |  |  |  |  |  |
| Shifting Shelf | 0.066 | 0.027 | -0.067 | 0.106 | 0.794 |  |  |  |  |
| Shifting Tray | 0.042 | 0.017 | -0.042 | 0.067 | 0.148 | 0.921 |  |  |  |
| WM Boxes | 0.018 | 0.007 | -0.019 | 0.028 | 0.063 | 0.040 | 1.000 |  |  |
| WM Grid | 0.033 | 0.013 | -0.033 | 0.052 | 0.116 | 0.073 | 0.031 | 0.968 |  |
| WM Updating | 0.010 | 0.004 | -0.010 | 0.016 | 0.036 | 0.022 | 0.009 | 0.018 | 1.010 |

#### **Run 2**

Table S15. Model estimates for all models and their comparison against the 9-factor baseline model for run 2.

| **Model** | **WAIC** | **SE** | **WAIC difference** | **SE difference** | **p difference** |
| --- | --- | --- | --- | --- | --- |
| 1 factor | 3750.57 | 113.87 | -12.52 | 5.73 | .029 |
| MF2012 | 3755.17 | 113.87 | -10.22 | 5.41 | .057 |
| 3 factors | 3751.37 | 113.52 | -12.12 | 5.67 | .032 |
| 3 independent factors | 3762.91 | 113.62 | -6.35 | 4.39 | .148 |
| 2 factors 1 | 3754.87 | 113.56 | -10.37 | 5.57 | .062 |
| 2 factors 2 | 3759.17 | 113.46 | -8.22 | 4.75 | .083 |
| 2 factors 3 | 3753.73 | 113.72 | -10.94 | 5.17 | .034 |

Table S16. Factor loadings obtained in run 2.

| **Model** | **Latent factor** | **Task** | **Estimate** | **Post.sd** | **95% credible interval** | |
| --- | --- | --- | --- | --- | --- | --- |
| 1 factor | Common | Inhibition Cylinder | 1.00 |  |  |  |
|  |  | Inhibition Boxes | 3.34 | 4.13 | -7.04 | 11.00 |
|  |  | Inhibition Grid | -3.38 | 4.21 | -10.93 | 7.74 |
|  |  | Shifting Shelf | 11.75 | 10.87 | -20.68 | 26.40 |
|  |  | Shifting Boxes | 5.34 | 5.61 | -10.32 | 14.56 |
|  |  | Shifting Tray | 7.56 | 7.60 | -14.57 | 19.15 |
|  |  | WM Updating | 1.80 | 3.09 | -4.78 | 8.01 |
|  |  | WM Boxes | 3.19 | 4.02 | -6.96 | 10.68 |
|  |  | WM Grid | 5.79 | 5.92 | -10.88 | 15.34 |
| MF2012 | WM | WM Updating | 1.00 |  |  |  |
|  |  | WM Boxes | -2.06 | 6.70 | -16.47 | 12.71 |
|  |  | WM Grid | 1.46 | 6.61 | -13.55 | 15.94 |
|  | Shifting | Shifting Boxes | 1.00 |  |  |  |
|  |  | Shifting Shelf | 1.33 | 7.37 | -15.05 | 16.30 |
|  |  | Shifting Tray | 1.28 | 7.61 | -16.02 | 16.78 |
|  | Common | Inhibition Cylinder | 1.00 |  |  |  |
|  |  | Inhibition Grid | -2.39 | 5.47 | -11.79 | 10.74 |
|  |  | Inhibition Boxes | 2.57 | 5.61 | -11.24 | 12.54 |
|  |  | Shifting Shelf | 8.85 | 12.15 | -21.02 | 25.68 |
|  |  | Shifting Boxes | 4.16 | 7.00 | -13.06 | 15.71 |
|  |  | Shifting Tray | 5.51 | 8.58 | -15.45 | 18.71 |
|  |  | WM Updating | 1.66 | 4.99 | -10.00 | 11.73 |
|  |  | WM Boxes | 2.54 | 5.58 | -11.00 | 13.04 |
|  |  | WM Grid | 4.85 | 8.16 | -15.29 | 18.27 |
| 3 factor | WM | WM Updating | 1.00 |  |  |  |
|  |  | WM Boxes | 1.33 | 4.38 | -9.57 | 10.33 |
|  |  | WM Grid | 4.78 | 7.64 | -14.60 | 18.42 |
|  | Shifting | Shifting Boxes | 1.00 |  |  |  |
|  |  | Shifting Shelf | 4.82 | 3.47 | 1.29 | 14.27 |
|  |  | Shifting Tray | 2.73 | 2.17 | 0.71 | 8.80 |
|  | Inhibition | Inhibition Cylinder | 1.00 |  |  |  |
|  |  | Inhibition Grid | -0.73 | 7.59 | -16.75 | 16.59 |
|  |  | Inhibition Boxes | 1.06 | 4.09 | -8.41 | 10.10 |
| 3 independent factors | WM | WM Updating | 1.00 |  |  |  |
|  |  | WM Boxes | -1.58 | 6.79 | -16.29 | 12.61 |
|  |  | WM Grid | 2.25 | 6.95 | -12.88 | 16.90 |
|  | Shifting | Shifting Boxes | 1.00 |  |  |  |
|  |  | Shifting Shelf | 4.23 | 4.24 | 0.12 | 14.16 |
|  |  | Shifting Tray | 3.49 | 4.14 | 0.13 | 13.59 |
|  | Inhibition | Inhibition Cylinder | 1.00 |  |  |  |
|  |  | Inhibition Grid | 1.62 | 7.17 | -14.78 | 16.61 |
|  |  | Inhibition Boxes | 1.96 | 7.27 | -14.34 | 17.08 |
| 2 factors 1 | WM + Shifting | WM Updating | 1.00 |  |  |  |
|  |  | WM Boxes | 3.88 | 2.76 | -0.32 | 10.39 |
|  |  | WM Grid | 6.83 | 3.60 | 1.56 | 15.39 |
|  |  | Shifting Boxes | 5.55 | 3.16 | 0.96 | 13.04 |
|  |  | Shifting Shelf | 11.87 | 5.01 | 3.88 | 23.08 |
|  |  | Shifting Tray | 8.12 | 4.03 | 2.27 | 17.58 |
|  | Inhibition | Inhibition Cylinder | 1.00 |  |  |  |
|  |  | Inhibition Grid | 1.49 | 7.16 | -14.64 | 16.36 |
|  |  | Inhibition Boxes | 1.93 | 7.32 | -14.55 | 17.00 |
| 2 factors 2 | WM + Inhibition | WM Updating | 1.00 |  |  |  |
|  |  | WM Boxes | 0.58 | 6.91 | -14.27 | 13.50 |
|  |  | WM Grid | 2.86 | 11.38 | -19.27 | 21.81 |
|  |  | Inhibition Grid | 1.01 | 7.41 | -14.43 | 14.60 |
|  |  | Inhibition Cylinder | 1.94 | 10.70 | -19.30 | 20.16 |
|  |  | Inhibition Boxes | 0.75 | 6.42 | -12.88 | 13.60 |
|  | Shifting | Shifting Boxes | 1.00 |  |  |  |
|  |  | Shifting Shelf | 4.54 | 4.05 | 0.88 | 14.42 |
|  |  | Shifting Tray | 3.75 | 3.71 | 0.63 | 13.54 |
| 2 factors 3 | Inhibition + Shifting | Shifting Boxes | 1.00 |  |  |  |
|  |  | Shifting Shelf | 10.70 | 5.68 | 2.28 | 23.59 |
|  |  | Shifting Tray | 6.19 | 3.92 | 1.18 | 15.99 |
|  |  | Inhibition Grid | -3.19 | 2.44 | -9.39 | -0.02 |
|  |  | Inhibition Cylinder | 4.42 | 2.98 | 0.64 | 11.82 |
|  |  | Inhibition Boxes | 3.02 | 2.54 | -0.40 | 9.31 |
|  | WM | WM Updating | 1.00 |  |  |  |
|  |  | WM Boxes | -1.49 | 6.76 | -16.41 | 12.65 |
|  |  | WM Grid | 2.39 | 6.88 | -12.19 | 17.05 |

Table S17. Model comparisons between the 1-factor model and all competing models for run 2.

| **Model** | **WAIC difference** | **SE difference** | **p difference** |
| --- | --- | --- | --- |
| MF2012 | -2.30 | 0.98 | .019 |
| 3 factors | -0.34 | 1.87 | .856 |
| 3 independent factors | -6.17 | 3.79 | .103 |
| 2 factors 1 | -2.15 | 2.48 | .386 |
| 2 factors 2 | -4.30 | 4.16 | .301 |
| 2 factors 3 | -1.58 | 3.91 | .686 |

Table S18. Proportion of variance in performance explained by the shared factor on each of the nine EF tasks for run 2 (median and 95% credible interval).

| **Task** | **Proportion of variance explained (median)** | **95% credible interval** | |
| --- | --- | --- | --- |
| Inhibition Boxes | .034 | .001 | .135 |
| Inhibition Cylinder | .002 | .001 | .008 |
| Inhibition Grid | .035 | .001 | .130 |
| Shifting Boxes | .084 | .011 | .225 |
| Shifting Shelf | .419 | .174 | .807 |
| Shifting Tray | .172 | .050 | .368 |
| WM Boxes | .030 | .000 | .127 |
| WM Grid | .101 | .014 | .257 |
| WM Updating | .011 | .000 | .072 |

Table S19. Variance-covariance matrix for the 1-factor model in run 2.

| Task | Inhibition Boxes | Inhibition Cylinder | Inhibition Grid | Shifting Boxes | Shifting Shelf | Shifting Tray | WM Boxes | WM Grid | WM Updating |
| --- | --- | --- | --- | --- | --- | --- | --- | --- | --- |
| Inhibition Boxes | 1.016 |  |  |  |  |  |  |  |  |
| Inhibition Cylinder | 0.009 | 1.002 |  |  |  |  |  |  |  |
| Inhibition Grid | -0.031 | -0.009 | 1.009 |  |  |  |  |  |  |
| Shifting Boxes | 0.050 | 0.015 | -0.050 | 1.004 |  |  |  |  |  |
| Shifting Shelf | 0.109 | 0.033 | -0.110 | 0.175 | 0.941 |  |  |  |  |
| Shifting Tray | 0.070 | 0.021 | -0.071 | 0.112 | 0.247 | 0.989 |  |  |  |
| WM Boxes | 0.030 | 0.009 | -0.030 | 0.047 | 0.104 | 0.067 | 1.011 |  |  |
| WM Grid | 0.054 | 0.016 | -0.054 | 0.086 | 0.190 | 0.122 | 0.051 | 1.000 |  |
| WM Updating | 0.017 | 0.005 | -0.017 | 0.027 | 0.059 | 0.038 | 0.016 | 0.029 | 1.011 |

#### **CFA excluding Working Memory Updating**

We also conducted two runs of the CFA for which we excluded the WM Updating task. For both runs, most models converged. In run 1, some Rhat values for the 2factor3 model were large (the largest Rhat was 1.15), in run 2, this was the case for the 2factor1 model (the largest Rhat was 1.47), indicating convergence issues for these models. In addition, the Gelman-Rubin psfr (potential scale reduction factor) was larger than 1.2 for these models, further indicating convergence issues. Yet, we found WAIC values to be very similar between runs (Tables S20 and S21), indicating that we were able to consistently estimate model fits.

Again, for all but the 9-factor model there was a warning for the presence of divergent transitions, although the percentages of divergent transitions were small: occurring for between 0.68% and 11.56% of the samples (run 2: between 0.06 and 7.82%). Warnings that the Bulk or Tail Effective sample sizes were low occurred for the 3-factor, MF2012, and all 2-factor models (in run 2 we received these warnings for the same models, and in addition for the 3 independent factor model), and we received warnings about small effective sample sizes for some parameters for the 2factor3 model (and in run 2 for the 2factor1 model). Although we found that some of the models had difficulties estimating some of the parameter values, the consistent WAIC values between runs suggested that overall model fit was still consistently estimated. Factor loadings for all models for both runs can be found in Tables S22 and S23.

We then compared each model against the 9-factor baseline model (Table S20; run 2: Table 21). We found that the 1-factor, MF2012, 3-factor, and 2factor3 models fit the data significantly better than the baseline model in both runs. We investigated whether the 1-factor model would fit the data significantly better than the competing models and found that the 1-factor model had a better fit than the MF2012 and the 3 independent factor model in both runs (Tables S24 and S25). The proportion of variance in performance explained by the shared factor on each of the eight EF tasks is given in Tables S26 and S27 and reveals the same hierarchical ordering of tasks as the main analyses.

We also compared the variance-covariance matrices of both models (run 1: Table S28; run 2: Table S29) with the correlations between tasks from Table S7 in order to check whether the 1-factor-model could pick up the correlations between the tasks. We found that in both runs, the model picked up most correlations involving the Shifting Shelf task, but not the correlations involving the Inhibition Cylinder task.

Table S20. Model estimates for all models and their comparison against the 9-factor baseline model for run 1 of the CFA without the WM Updating task.

| **Model** | **WAIC** | **SE** | **WAIC difference** | **SE difference** | **p difference** |
| --- | --- | --- | --- | --- | --- |
| 1-factor | 3223.33 | 93.92 | -13.64 | 5.97 | .022 |
| MF2012 | 3226.22 | 93.96 | -12.20 | 5.83 | .036 |
| 3 factors | 3226.93 | 93.67 | -11.84 | 5.63 | .035 |
| 3 independent factors | 3238.07 | 93.75 | -6.27 | 4.48 | .162 |
| 2factors 1 | 3229.63 | 93.37 | -10.49 | 5.58 | .060 |
| 2factors 2 | 3234.39 | 93.65 | -8.11 | 4.78 | .090 |
| 2factors 3 | 3229.74 | 94.01 | -10.44 | 5.15 | .043 |

Table S21. Model estimates for all models and their comparison against the 9-factor baseline model for run 2 of the CFA without the WM Updating task.

| **Model** | **WAIC** | **SE** | **WAIC difference** | **SE difference** | **p difference** |
| --- | --- | --- | --- | --- | --- |
| 1-factor | 3223.20 | 93.93 | -13.75 | 5.96 | .021 |
| MF2012 | 3226.32 | 93.97 | -12.19 | 5.81 | .036 |
| 3 factors | 3226.36 | 93.64 | -12.17 | 5.64 | .031 |
| 3 independent factors | 3237.97 | 93.75 | -6.37 | 4.45 | .152 |
| 2factors 1 | 3231.67 | 93.34 | -9.51 | 5.40 | .078 |
| 2factors 2 | 3234.61 | 93.68 | -8.05 | 4.72 | .088 |
| 2factors 3 | 3228.68 | 94.02 | -11.01 | 5.19 | .034 |

Table S22. Factor loadings obtained in run 1 for the CFA without the WM Updating task.

| **Model** | **Latent factor** | **Task** | **Estimate** | **Post.sd** | **95% credible interval** | |
| --- | --- | --- | --- | --- | --- | --- |
| 1 factor | Common | Inhibition Cylinder | 1.00 |  |  |  |
|  |  | Inhibition Boxes | 3.66 | 2.73 | -0.74 | 9.96 |
|  |  | Inhibition Grid | -3.85 | 2.73 | -10.24 | 0.39 |
|  |  | Shifting Shelf | 13.54 | 5.51 | 4.38 | 25.74 |
|  |  | Shifting Boxes | 5.63 | 3.17 | 0.99 | 13.14 |
|  |  | Shifting Tray | 8.39 | 4.05 | 2.27 | 17.68 |
|  |  | WM Boxes | 3.73 | 2.73 | -0.52 | 10.16 |
|  |  | WM Grid | 6.15 | 3.31 | 1.23 | 13.93 |
| MF2012 | WM | WM Boxes | 1.00 |  |  |  |
|  |  | WM Grid | 2.06 | 6.82 | -14.02 | 15.65 |
|  | Shifting | Shifting Boxes | 1.00 |  |  |  |
|  |  | Shifting Shelf | 0.54 | 7.27 | -15.48 | 15.35 |
|  |  | Shifting Tray | 0.97 | 7.87 | -15.80 | 17.21 |
|  | Common | Inhibition Cylinder | 1.00 |  |  |  |
|  |  | Inhibition Boxes | 3.29 | 3.89 | -5.41 | 11.12 |
|  |  | Inhibition Grid | -3.20 | 4.09 | -10.51 | 7.06 |
|  |  | Shifting Shelf | 11.60 | 8.69 | -15.40 | 25.26 |
|  |  | Shifting Boxes | 4.70 | 4.82 | -7.97 | 13.28 |
|  |  | Shifting Tray | 7.26 | 6.25 | -10.78 | 18.02 |
|  |  | WM Boxes | 3.02 | 3.86 | -5.94 | 10.69 |
|  |  | WM Grid | 5.31 | 5.05 | -7.79 | 14.78 |
| 3 factor | WM | WM Boxes | 1.00 |  |  |  |
|  |  | WM Grid | 3.66 | 4.80 | -8.87 | 14.33 |
|  | Shifting | Shifting Boxes | 1.00 |  |  |  |
|  |  | Shifting Shelf | 4.84 | 3.47 | 1.30 | 14.48 |
|  |  | Shifting Tray | 2.75 | 2.18 | 0.72 | 8.78 |
|  | Inhibition | Inhibition Cylinder | 1.00 |  |  |  |
|  |  | Inhibition Boxes | 0.97 | 4.19 | -8.95 | 10.47 |
|  |  | Inhibition Grid | -0.24 | 7.75 | -15.94 | 17.36 |
| 3 independent factors | WM | WM Boxes | 1.00 |  |  |  |
|  |  | WM Grid | 3.26 | 5.92 | -11.27 | 15.53 |
|  | Shifting | Shifting Boxes | 1.00 |  |  |  |
|  |  | Shifting Shelf | 4.76 | 3.60 | 0.98 | 14.65 |
|  |  | Shifting Tray | 3.81 | 3.34 | 0.72 | 13.48 |
|  | Inhibition | Inhibition Cylinder | 1.00 |  |  |  |
|  |  | Inhibition Boxes | 1.81 | 7.44 | -14.91 | 17.17 |
|  |  | Inhibition Grid | 1.70 | 7.34 | -15.01 | 17.20 |
| 2 factors 1 | WM + Shifting | WM Boxes | 1.00 |  |  |  |
|  |  | WM Grid | 5.00 | 2.99 | 1.01 | 12.33 |
|  |  | Shifting Boxes | 4.20 | 2.72 | 0.59 | 10.91 |
|  |  | Shifting Shelf | 10.09 | 5.14 | 2.58 | 22.06 |
|  |  | Shifting Tray | 6.26 | 3.58 | 1.52 | 15.03 |
|  | Inhibition | Inhibition Cylinder | 1.00 |  |  |  |
|  |  | Inhibition Boxes | 1.81 | 7.24 | -15.04 | 16.92 |
|  |  | Inhibition Grid | 2.01 | 7.18 | -14.22 | 16.76 |
| 2 factors 2 | WM + Inhibition | WM Boxes | 1.00 |  |  |  |
|  |  | WM Grid | 3.97 | 9.51 | -16.96 | 20.36 |
|  |  | Inhibition Cylinder | 3.66 | 9.60 | -17.75 | 19.92 |
|  |  | Inhibition Boxes | 1.32 | 5.58 | -11.00 | 12.87 |
|  |  | Inhibition Grid | 1.98 | 6.36 | -12.37 | 14.21 |
|  | Shifting | Shifting Boxes | 1.00 |  |  |  |
|  |  | Shifting Shelf | 4.45 | 4.09 | 0.67 | 14.43 |
|  |  | Shifting Tray | 3.51 | 3.86 | 0.48 | 13.11 |
| 2 factors 3 | Inhibition + Shifting | Shifting Boxes | 1.00 |  |  |  |
|  |  | Shifting Shelf | 8.60 | 8.73 | -17.54 | 23.14 |
|  |  | Shifting Tray | 5.05 | 5.72 | -11.18 | 15.87 |
|  |  | Inhibition Cylinder | 3.48 | 4.10 | -7.64 | 11.61 |
|  |  | Inhibition Boxes | 2.40 | 3.13 | -5.62 | 8.60 |
|  |  | Inhibition Grid | -2.56 | 3.27 | -8.78 | 5.80 |
|  | WM | WM Boxes | 1.00 |  |  |  |
|  |  | WM Grid | 3.12 | 5.81 | -10.34 | 15.37 |

Table S23. Factor loadings obtained in run 2 for the CFA without the WM Updating task.

| **Model** | **Latent factor** | **Task** | **Estimate** | **Post.sd** | **95% credible interval** | |
| --- | --- | --- | --- | --- | --- | --- |
| 1 factor | Common | Inhibition Cylinder | 1.00 |  |  |  |
|  |  | Inhibition Boxes | 3.72 | 2.81 | -0.71 | 10.26 |
|  |  | Inhibition Grid | -3.86 | 2.73 | -10.20 | 0.38 |
|  |  | Shifting Shelf | 13.46 | 5.52 | 4.40 | 25.71 |
|  |  | Shifting Boxes | 5.68 | 3.22 | 1.02 | 13.22 |
|  |  | Shifting Tray | 8.48 | 4.15 | 2.32 | 18.11 |
|  |  | WM Boxes | 3.72 | 2.73 | -0.48 | 10.24 |
|  |  | WM Grid | 6.16 | 3.30 | 1.22 | 13.86 |
| MF2012 | WM | WM Boxes | 1.00 |  |  |  |
|  |  | WM Grid | 2.25 | 6.93 | -13.79 | 16.07 |
|  | Shifting | Shifting Boxes | 1.00 |  |  |  |
|  |  | Shifting Shelf | 0.64 | 7.55 | -15.43 | 16.04 |
|  |  | Shifting Tray | 0.78 | 8.03 | -16.78 | 17.05 |
|  | Common | Inhibition Cylinder | 1.00 |  |  |  |
|  |  | Inhibition Boxes | 3.07 | 4.07 | -7.01 | 11.39 |
|  |  | Inhibition Grid | -3.07 | 4.34 | -10.79 | 7.87 |
|  |  | Shifting Shelf | 10.97 | 9.90 | -19.09 | 25.29 |
|  |  | Shifting Boxes | 4.51 | 5.01 | -9.03 | 13.55 |
|  |  | Shifting Tray | 6.92 | 6.94 | -13.00 | 18.37 |
|  |  | WM Boxes | 2.95 | 3.96 | -6.55 | 10.73 |
|  |  | WM Grid | 5.08 | 5.71 | -9.62 | 15.35 |
| 3 factor | WM | WM Boxes | 1.00 |  |  |  |
|  |  | WM Grid | 3.74 | 4.93 | -9.35 | 14.53 |
|  | Shifting | Shifting Boxes | 1.00 |  |  |  |
|  |  | Shifting Shelf | 4.80 | 3.41 | 1.31 | 14.30 |
|  |  | Shifting Tray | 2.71 | 2.10 | 0.72 | 8.76 |
|  | Inhibition | Inhibition Cylinder | 1.00 |  |  |  |
|  |  | Inhibition Boxes | 1.13 | 4.14 | -8.28 | 10.48 |
|  |  | Inhibition Grid | -0.60 | 7.80 | -16.78 | 16.73 |
| 3 independent factors | WM | WM Boxes | 1.00 |  |  |  |
|  |  | WM Grid | 3.28 | 5.88 | -10.57 | 15.44 |
|  | Shifting | Shifting Boxes | 1.00 |  |  |  |
|  |  | Shifting Shelf | 4.40 | 4.10 | 0.71 | 14.39 |
|  |  | Shifting Tray | 3.56 | 3.91 | 0.54 | 13.68 |
|  | Inhibition | Inhibition Cylinder | 1.00 |  |  |  |
|  |  | Inhibition Boxes | 2.05 | 7.39 | -14.57 | 17.42 |
|  |  | Inhibition Grid | 1.77 | 7.39 | -14.81 | 17.21 |
| 2 factors 1 | WM + Shifting | WM Boxes | 1.00 |  |  |  |
|  |  | WM Grid | 1.45 | 6.21 | -11.70 | 11.28 |
|  |  | Shifting Boxes | 1.19 | 5.39 | -10.51 | 10.23 |
|  |  | Shifting Shelf | 2.74 | 12.14 | -22.18 | 20.61 |
|  |  | Shifting Tray | 1.79 | 7.86 | -14.84 | 14.17 |
|  | Inhibition | Inhibition Cylinder | 1.00 |  |  |  |
|  |  | Inhibition Boxes | 1.76 | 7.53 | -15.27 | 17.16 |
|  |  | Inhibition Grid | 1.41 | 7.22 | -15.14 | 16.38 |
| 2 factors 2 | WM + Inhibition | WM Boxes | 1.00 |  |  |  |
|  |  | WM Grid | 3.97 | 9.60 | -17.42 | 20.55 |
|  |  | Inhibition Cylinder | 3.75 | 9.70 | -17.83 | 20.42 |
|  |  | Inhibition Boxes | 1.33 | 5.56 | -11.23 | 12.95 |
|  |  | Inhibition Grid | 1.96 | 6.33 | -12.30 | 14.30 |
|  | Shifting | Shifting Boxes | 1.00 |  |  |  |
|  |  | Shifting Shelf | 4.21 | 4.61 | -6.29 | 14.62 |
|  |  | Shifting Tray | 3.41 | 4.47 | -6.43 | 13.98 |
| 2 factors 3 | Inhibition + Shifting | Shifting Boxes | 1.00 |  |  |  |
|  |  | Shifting Shelf | 10.74 | 5.61 | 2.37 | 23.43 |
|  |  | Shifting Tray | 6.18 | 3.92 | 1.24 | 16.00 |
|  |  | Inhibition Cylinder | 4.37 | 2.89 | 0.67 | 11.53 |
|  |  | Inhibition Boxes | 3.00 | 2.46 | -0.36 | 9.18 |
|  |  | Inhibition Grid | -3.21 | 2.45 | -9.26 | -0.01 |
|  | WM | WM Boxes | 1.00 |  |  |  |
|  |  | WM Grid | 3.12 | 6.05 | -11.55 | 15.37 |

Table S24. Model comparisons between the 1-factor model and all competing models for run 1 for the CFA without the Working Memory Updating task.

| **Model** | **WAIC difference** | **SE difference** | **p difference** |
| --- | --- | --- | --- |
| MF2012 | -1.44 | 0.60 | .016 |
| 3 factors | -1.80 | 1.84 | .328 |
| 3 independent factors | -7.37 | 3.75 | .049 |
| 2factors 1 | -3.15 | 2.66 | .236 |
| 2factors 2 | -5.53 | 4.14 | .182 |
| 2factors 3 | -3.20 | 3.84 | .405 |

Table S25. Model comparisons between the 1-factor model and all competing models for run 2 for the CFA without the Working Memory Updating task.

| **Model** | **WAIC difference** | **SE difference** | **p difference** |
| --- | --- | --- | --- |
| MF2012 | -1.56 | 0.58 | .007 |
| 3 factors | -1.58 | 1.83 | .388 |
| 3 independent factors | -7.38 | 3.74 | .048 |
| 2factors 1 | -4.23 | 2.76 | .125 |
| 2factors 2 | -5.70 | 4.14 | .168 |
| 2factors 3 | -2.74 | 3.84 | .475 |

Table S26. Proportion of variance in performance explained by the shared factor on each of the seven EF tasks for run 1 for the CFA without the Working Memory Updating task (median and 95% credible interval).

| **Task** | **Proportion of variance explained (median)** | **95% credible interval** | |
| --- | --- | --- | --- |
| Inhibition Boxes | .034 | .001 | .130 |
| Inhibition Cylinder | .003 | .001 | .016 |
| Inhibition Grid | .037 | .001 | .129 |
| Shifting Boxes | .079 | .010 | .211 |
| Shifting Shelf | .470 | .203 | .902 |
| Shifting Tray | .174 | .053 | .369 |
| WM Boxes | .035 | .001 | .134 |
| WM Grid | .096 | .014 | .246 |

Table S27. Proportion of variance in performance explained by the shared factor on each of the seven EF tasks for run 2 for the CFA without the Working Memory Updating task (median and 95% credible interval).

| **Task** | **Proportion of variance explained (median)** | **95% credible interval** | |
| --- | --- | --- | --- |
| Inhibition Boxes | .035 | .001 | .132 |
| Inhibition Cylinder | .003 | .001 | .015 |
| Inhibition Grid | .037 | .001 | .128 |
| Shifting Boxes | .080 | .011 | .213 |
| Shifting Shelf | .464 | .199 | .873 |
| Shifting Tray | .176 | .054 | .375 |
| WM Boxes | .034 | .001 | .131 |
| WM Grid | .097 | .014 | .242 |

Table S28. Variance-covariance matrix for the 1-factor model in run 1 for the CFA without the Working Memory Updating task.

| Task | Inhibition Boxes | Inhibition Cylinder | Inhibition Grid | Shifting Boxes | Shifting Shelf | Shifting Tray | WM Boxes | WM Grid |
| --- | --- | --- | --- | --- | --- | --- | --- | --- |
| Inhibition Boxes | 1.049 |  |  |  |  |  |  |  |
| Inhibition Cylinder | 0.179 | 0.995 |  |  |  |  |  |  |
| Inhibition Grid | -0.065 | -0.018 | 1.047 |  |  |  |  |  |
| Shifting Boxes | 0.095 | 0.026 | -0.100 | 1.079 |  |  |  |  |
| Shifting Shelf | 0.230 | 0.063 | -0.242 | 0.353 | 1.355 |  |  |  |
| Shifting Tray | 0.142 | 0.039 | -0.150 | 0.219 | 0.527 | 1.158 |  |  |
| WM Boxes | 0.063 | 0.017 | -0.067 | 0.097 | 0.234 | 0.145 | 1.043 |  |
| WM Grid | 0.104 | 0.028 | -0.110 | 0.160 | 0.386 | 0.239 | 0.106 | 1.094 |

Table S29. Variance-covariance matrix for the 1-factor model in run 2 for the CFA without the Working Memory Updating task.

| Task | Inhibition Boxes | Inhibition Cylinder | Inhibition Grid | Shifting Boxes | Shifting Shelf | Shifting Tray | WM Boxes | WM Grid |
| --- | --- | --- | --- | --- | --- | --- | --- | --- |
| Inhibition Boxes | 1.049 |  |  |  |  |  |  |  |
| Inhibition Cylinder | 0.017 | 0.998 |  |  |  |  |  |  |
| Inhibition Grid | -0.066 | -0.018 | 1.046 |  |  |  |  |  |
| Shifting Boxes | 0.098 | 0.026 | -0.101 | 1.082 |  |  |  |  |
| Shifting Shelf | 0.232 | 0.062 | -0.240 | 0.354 | 1.354 |  |  |  |
| Shifting Tray | 0.146 | 0.039 | -0.151 | 0.223 | 0.529 | 1.160 |  |  |
| WM Boxes | 0.064 | 0.017 | -0.066 | 0.098 | 0.232 | 0.146 | 1.045 |  |
| WM Grid | 0.106 | 0.028 | -0.110 | 0.162 | 0.384 | 0.242 | 0.106 | 1.092 |

#### **CFA with adjusted priors**

In the main analysis, the default priors of blavaan ^4^ were chosen as these were deemed to result in a relatively conservative model fit. In order to get a more even weighting across the 0-1 range, we ran a new analysis with Gamma (1, 1) and a second analysis with Gamma (1, 2). All models contained all nine EF tasks.

##### **Gamma (1,1) prior**

Most models converged. Some Rhat values for the 2factor1 model were large (the largest Rhat was 1.61), indicating convergence issues for this model. In addition, the Gelman-Rubin psfr (potential scale reduction factor) was larger than 1.2 for this model, further indicating convergence issues. We received a number of warnings when fitting the models. For all but the 9-factor model there was a warning for the presence of divergent transitions, although the percentages of divergent transitions were small: occurring for between 0.08% and 18.14% of the samples. In addition, we received warnings that the Bulk or Tail Effective sample sizes were low for the 3-factor, 3 independent factors, and 2factor1 models. Factor loadings for all models can be found in Table S30.

We then compared each model against the 9-factor baseline model (Table S31). We found the 1-factor, 3-factor model, and 2factor3 models to fit the data significantly better than the baseline model. We investigated whether the 1-factor model would fit the data significantly better than the competing models and found that the 1-factor model had a better fit than the MF2012 model (Table S32). The proportion of variance in performance explained by the shared factor on each of the nine EF tasks is given in Table S33.

We also compared the variance-covariance matrix of the 1-factor model (Table S34) with the correlations between tasks from Table S7 in order to check whether the 1-factor-model could pick up the correlations between the tasks and found no qualitative differences to the results from the main analysis.

Table S30. Factor loadings obtained for the CFA with the prior adjusted to Gamma (1,1).

| **Model** | **Latent factor** | **Task** | **Estimate** | **Post.sd** | **95% credible interval** | |
| --- | --- | --- | --- | --- | --- | --- |
| 1 factor | Common | Inhibition Cylinder | 1.00 |  |  |  |
|  |  | Inhibition Boxes | 4.22 | 3.05 | -0.98 | 11.02 |
|  |  | Inhibition Grid | -4.33 | 3.03 | -11.23 | 0.74 |
|  |  | Shifting Shelf | 14.67 | 5.31 | 5.58 | 26.14 |
|  |  | Shifting Boxes | 6.83 | 3.48 | 1.42 | 14.75 |
|  |  | Shifting Tray | 9.69 | 4.18 | 2.99 | 19.26 |
|  |  | WM Updating | 2.33 | 2.73 | -2.45 | 8.44 |
|  |  | WM Boxes | 4.07 | 2.98 | -0.88 | 10.67 |
|  |  | WM Grid | 7.34 | 3.62 | 1.65 | 15.52 |
| MF2012 | WM | WM Updating | 1.00 |  |  |  |
|  |  | WM Boxes | -1.98 | 6.86 | -16.49 | 13.09 |
|  |  | WM Grid | 1.55 | 6.80 | -13.01 | 16.47 |
|  | Shifting | Shifting Boxes | 1.00 |  |  |  |
|  |  | Shifting Shelf | 1.26 | 7.58 | -15.31 | 16.73 |
|  |  | Shifting Tray | 1.14 | 7.67 | -15.74 | 16.70 |
|  | Common | Inhibition Cylinder | 1.00 |  |  |  |
|  |  | Inhibition Grid | -3.01 | 5.07 | -11.96 | 9.54 |
|  |  | Inhibition Boxes | 3.32 | 5.05 | -8.96 | 12.53 |
|  |  | Shifting Shelf | 10.77 | 10.72 | -19.13 | 26.24 |
|  |  | Shifting Boxes | 5.03 | 6.36 | -11.74 | 15.94 |
|  |  | Shifting Tray | 6.83 | 7.59 | -13.41 | 19.10 |
|  |  | WM Updating | 2.03 | 4.68 | -8.44 | 11.70 |
|  |  | WM Boxes | 3.00 | 5.15 | -9.44 | 12.50 |
|  |  | WM Grid | 5.81 | 7.20 | -13.19 | 18.11 |
| 3 factor | WM | WM Updating | 1.00 |  |  |  |
|  |  | WM Boxes | 1.52 | 4.28 | -9.36 | 10.35 |
|  |  | WM Grid | 5.21 | 7.41 | -13.93 | 18.63 |
|  | Shifting | Shifting Boxes | 1.00 |  |  |  |
|  |  | Shifting Shelf | 4.99 | 3.51 | 1.29 | 14.75 |
|  |  | Shifting Tray | 2.81 | 2.17 | 0.74 | 8.97 |
|  | Inhibition | Inhibition Cylinder | 1.00 |  |  |  |
|  |  | Inhibition Grid | -0.34 | 8.00 | -16.43 | 17.34 |
|  |  | Inhibition Boxes | 0.98 | 4.35 | -9.12 | 10.64 |
| 3 independent factors | WM | WM Updating | 1.00 |  |  |  |
|  |  | WM Boxes | -1.48 | 6.92 | -16.65 | 12.99 |
|  |  | WM Grid | 2.28 | 7.17 | -13.28 | 16.98 |
|  | Shifting | Shifting Boxes | 1.00 |  |  |  |
|  |  | Shifting Shelf | 4.48 | 4.61 | -4.87 | 15.06 |
|  |  | Shifting Tray | 3.61 | 4.51 | -5.66 | 14.42 |
|  | Inhibition | Inhibition Cylinder | 1.00 |  |  |  |
|  |  | Inhibition Grid | 1.84 | 7.29 | -14.29 | 17.32 |
|  |  | Inhibition Boxes | 1.92 | 7.32 | -14.73 | 17.25 |
| 2 factors 1 | WM + Shifting | WM Updating | 1.00 |  |  |  |
|  |  | WM Boxes | 0.49 | 5.16 | -9.87 | 9.41 |
|  |  | WM Grid | 1.31 | 7.97 | -13.75 | 14.13 |
|  |  | Shifting Boxes | 0.98 | 6.56 | -11.78 | 11.82 |
|  |  | Shifting Shelf | 1.92 | 13.38 | -22.15 | 21.63 |
|  |  | Shifting Tray | 1.17 | 9.58 | -16.65 | 15.95 |
|  | Inhibition | Inhibition Cylinder | 1.00 |  |  |  |
|  |  | Inhibition Grid | 1.55 | 7.38 | -14.86 | 16.71 |
|  |  | Inhibition Boxes | 1.81 | 7.31 | -14.72 | 16.77 |
| 2 factors 2 | WM + Inhibition | WM Updating | 1.00 |  |  |  |
|  |  | WM Boxes | 0.76 | 7.00 | -14.16 | 13.86 |
|  |  | WM Grid | 3.09 | 11.35 | -19.29 | 21.98 |
|  |  | Inhibition Grid | 1.25 | 7.40 | -14.51 | 15.07 |
|  |  | Inhibition Cylinder | 2.21 | 10.58 | -19.16 | 19.93 |
|  |  | Inhibition Boxes | 0.87 | 6.44 | -12.92 | 13.64 |
|  | Shifting | Shifting Boxes | 1.00 |  |  |  |
|  |  | Shifting Shelf | 4.63 | 3.81 | 0.88 | 14.44 |
|  |  | Shifting Tray | 3.83 | 3.82 | 0.65 | 14.26 |
| 2 factors 3 | Inhibition + Shifting | Shifting Boxes | 1.00 |  |  |  |
|  |  | Shifting Shelf | 10.73 | 5.63 | 2.32 | 23.33 |
|  |  | Shifting Tray | 6.14 | 3.83 | 1.23 | 15.59 |
|  |  | Inhibition Grid | -3.17 | 2.48 | -9.12 | -0.06 |
|  |  | Inhibition Cylinder | 4.46 | 3.06 | 0.70 | 11.97 |
|  |  | Inhibition Boxes | 3.03 | 2.50 | -0.35 | 9.31 |
|  | WM | WM Updating | 1.00 |  |  |  |
|  |  | WM Boxes | -1.64 | 6.87 | -16.44 | 12.78 |
|  |  | WM Grid | 2.31 | 7.05 | -12.80 | 16.86 |

Table S31. Model estimates for all models and their comparison against the 9-factor baseline model for the CFA with the prior adjusted to Gamma (1,1).

| **Model** | **WAIC** | **SE** | **WAIC difference** | **SE difference** | **p difference** |
| --- | --- | --- | --- | --- | --- |
| 1-factor | 3749.75 | 113.82 | -12.94 | 5.83 | .026 |
| MF2012 | 3754.64 | 113.57 | -10.50 | 5.50 | .056 |
| 3 factors | 3751.68 | 113.57 | -11.98 | 5.67 | .034 |
| 3 independent factors | 3763.32 | 113.65 | -6.16 | 4.39 | .160 |
| 2factors 1 | 3755.38 | 113.54 | -10.13 | 5.43 | .062 |
| 2factors 2 | 3759.16 | 113.50 | -8.24 | 4.74 | .082 |
| 2factors 3 | 3753.46 | 113.70 | -11.09 | 5.20 | .033 |

Table S32. Model comparisons between the 1-factor model and all competing models for the CFA with the prior adjusted to Gamma (1,1).

| **Model** | **WAIC difference** | **SE difference** | **p difference** |
| --- | --- | --- | --- |
| MF2012 | -2.44 | 0.92 | .008 |
| 3 factors | -0.96 | 1.91 | .614 |
| 3 independent factors | -6.78 | 3.85 | .078 |
| 2factors1 | -2.81 | 2.60 | .278 |
| 2factors2 | -4.70 | 4.20 | .262 |
| 2factors3 | -1.85 | 3.95 | .639 |

Table S33. Proportion of variance in performance explained by the shared factor on each of the nine EF tasks for the CFA with the Gamma (1,1) prior (median and 95% credible interval).

| **Task** | **Proportion of variance explained (median)** | **95% credible interval** | |
| --- | --- | --- | --- |
| Inhibition Boxes | .035 | .001 | .132 |
| Inhibition Cylinder | .002 | .001 | .009 |
| Inhibition Grid | .035 | .001 | .130 |
| Shifting Boxes | .087 | .012 | .227 |
| Shifting Shelf | .418 | .181 | .765 |
| Shifting Tray | .176 | .053 | .363 |
| WM Boxes | .031 | .000 | .127 |
| WM Grid | .105 | .014 | .262 |
| WM Updating | .011 | .000 | .075 |

Table S34. Variance-covariance matrix for the 1-factor-model in run 1 for the CFA with the prior adjusted to Gamma (1,1).

| Task | Inhibition Boxes | Inhibition Cylinder | Inhibition Grid | Shifting Boxes | Shifting Shelf | Shifting Tray | WM Boxes | WM Grid | WM Updating |
| --- | --- | --- | --- | --- | --- | --- | --- | --- | --- |
| Inhibition Boxes |  |  |  |  |  |  |  |  |  |
| Inhibition Cylinder | 0.013 |  |  |  |  |  |  |  |  |
| Inhibition Grid | -0.055 | -0.013 |  |  |  |  |  |  |  |
| Shifting Boxes | -0.086 | 0.020 | -0.088 |  |  |  |  |  |  |
| Shifting Shelf | -0.185 | 0.044 | -0.190 | 0.299 |  |  |  |  |  |
| Shifting Tray | 0.122 | 0.029 | -0.125 | 0.198 | 0.425 |  |  |  |  |
| WM Boxes | 0.051 | 0.012 | -0.053 | 0.083 | 0.178 | 0.118 |  |  |  |
| WM Grid | 0.093 | 0.022 | -0.095 | 0.150 | 0.322 | 0.213 | 0.089 |  |  |
| WM Updating | 0.029 | 0.007 | -0.030 | 0.047 | 0.102 | 0.067 | 0.028 | 0.051 |  |

##### **Gamma (1,2) prior**

All models converged. We received some warnings when fitting the models. For all but the 9-factor model there was a warning for the presence of divergent transitions, although the percentages of divergent transitions were small: occurring for between 0.22% and 13.12% of the samples. In addition, we received warnings that the Bulk or Tail Effective sample sizes were low for the 3-factor, MF2012, 3 independent factors, 2factor1, and 2factor2 models. Factor loadings for all models can be found in Table S35. We then compared each model against the 9-factor baseline model and found that the 1-factor and 3-factor models the data significantly better than the baseline model (Table S36). We found that the 1-factor model had a better fit than the MF2012 model (Table S37). The proportion of variance in performance explained by the shared factor on each of the nine EF tasks is given in Table S38. We also compared the variance-covariance matrix of the 1-factor model (Table S39) with the correlations between tasks from Table S7 in order to check whether the 1-factor-model could pick up the correlations between the tasks and found no qualitative differences to the results from the main analysis.

Table S35. Factor loadings obtained for the CFA with the prior adjusted to Gamma (1,2).

| **Model** | **Latent factor** | **Task** | **Estimate** | **Post.sd** | **95% credible interval** | |
| --- | --- | --- | --- | --- | --- | --- |
| 1 factor | Common | Inhibition Cylinder | 1.00 |  |  |  |
|  |  | Inhibition Boxes | 4.24 | 3.11 | -1.10 | 11.14 |
|  |  | Inhibition Grid | -4.37 | 3.02 | -11.18 | 0.61 |
|  |  | Shifting Shelf | 14.90 | 5.44 | 5.81 | 26.93 |
|  |  | Shifting Boxes | 6.84 | 3.51 | 1.36 | 14.92 |
|  |  | Shifting Tray | 9.69 | 4.22 | 3.14 | 19.28 |
|  |  | WM Updating | 2.30 | 2.66 | -2.37 | 8.22 |
|  |  | WM Boxes | 4.10 | 2.98 | -0.86 | 10.98 |
|  |  | WM Grid | 7.40 | 3.70 | 1.57 | 15.82 |
| MF2012 | WM | WM Updating | 1.00 |  |  |  |
|  |  | WM Boxes | -1.92 | 7.05 | -17.13 | 13.41 |
|  |  | WM Grid | 1.59 | 7.08 | -14.09 | 16.54 |
|  | Shifting | Shifting Boxes | 1.00 |  |  |  |
|  |  | Shifting Shelf | 0.85 | 7.74 | -16.14 | 16.18 |
|  |  | Shifting Tray | 0.98 | 7.73 | -15.93 | 16.73 |
|  | Common | Inhibition Cylinder | 1.00 |  |  |  |
|  |  | Inhibition Grid | -3.29 | 4.89 | -12.10 | 9.04 |
|  |  | Inhibition Boxes | 3.49 | 4.79 | -8.26 | 12.61 |
|  |  | Shifting Shelf | 11.56 | 9.61 | -17.14 | 25.93 |
|  |  | Shifting Boxes | 5.41 | 5.90 | -10.22 | 15.81 |
|  |  | Shifting Tray | 7.37 | 7.08 | -12.28 | 19.22 |
|  |  | WM Updating | 2.16 | 4.59 | -8.18 | 11.86 |
|  |  | WM Boxes | 3.12 | 4.81 | -8.38 | 12.27 |
|  |  | WM Grid | 6.29 | 6.47 | -10.85 | 18.45 |
| 3 factor | WM | WM Updating | 1.00 |  |  |  |
|  |  | WM Boxes | 1.47 | 4.58 | -9.84 | 10.83 |
|  |  | WM Grid | 5.10 | 7.81 | -14.63 | 18.94 |
|  | Shifting | Shifting Boxes | 1.00 |  |  |  |
|  |  | Shifting Shelf | 5.33 | 3.68 | 1.41 | 15.32 |
|  |  | Shifting Tray | 3.02 | 2.32 | 0.76 | 9.48 |
|  | Inhibition | Inhibition Cylinder | 1.00 |  |  |  |
|  |  | Inhibition Grid | -0.59 | 8.26 | -17.06 | 17.05 |
|  |  | Inhibition Boxes | 1.02 | 4.56 | -9.47 | 11.04 |
| 3 independent factors | WM | WM Updating | 1.00 |  |  |  |
|  |  | WM Boxes | -1.46 | 7.24 | -17.04 | 13.79 |
|  |  | WM Grid | 2.34 | 7.30 | -13.08 | 17.54 |
|  | Shifting | Shifting Boxes | 1.00 |  |  |  |
|  |  | Shifting Shelf | 4.45 | 4.90 | -7.57 | 15.31 |
|  |  | Shifting Tray | 3.54 | 4.76 | -8.14 | 14.15 |
|  | Inhibition | Inhibition Cylinder | 1.00 |  |  |  |
|  |  | Inhibition Grid | 1.58 | 7.60 | -15.42 | 16.95 |
|  |  | Inhibition Boxes | 1.68 | 7.59 | -15.08 | 17.18 |
| 2 factors 1 | WM + Shifting | WM Updating | 1.00 |  |  |  |
|  |  | WM Boxes | 4.10 | 2.84 | -0.26 | 10.43 |
|  |  | WM Grid | 7.05 | 3.48 | 1.70 | 15.19 |
|  |  | Shifting Boxes | 5.76 | 3.15 | 0.92 | 13.06 |
|  |  | Shifting Shelf | 12.45 | 5.17 | 4.22 | 23.91 |
|  |  | Shifting Tray | 8.22 | 3.87 | 2.53 | 17.38 |
|  | Inhibition | Inhibition Cylinder | 1.00 |  |  |  |
|  |  | Inhibition Grid | 1.78 | 7.52 | -14.93 | 16.83 |
|  |  | Inhibition Boxes | 1.56 | 7.39 | -15.15 | 16.78 |
| 2 factors 2 | WM + Inhibition | WM Updating | 1.00 |  |  |  |
|  |  | WM Boxes | 0.76 | 7.13 | -14.50 | 14.26 |
|  |  | WM Grid | 2.93 | 11.38 | -19.40 | 21.82 |
|  |  | Inhibition Grid | 1.21 | 7.46 | -14.28 | 15.20 |
|  |  | Inhibition Cylinder | 2.21 | 10.72 | -19.25 | 20.40 |
|  |  | Inhibition Boxes | 0.85 | 6.61 | -13.28 | 14.07 |
|  | Shifting | Shifting Boxes | 1.00 |  |  |  |
|  |  | Shifting Shelf | 4.46 | 4.88 | -7.28 | 15.16 |
|  |  | Shifting Tray | 3.61 | 4.82 | -8.06 | 14.76 |
| 2 factors 3 | Inhibition + Shifting | Shifting Boxes | 1.00 |  |  |  |
|  |  | Shifting Shelf | 11.39 | 5.68 | 2.70 | 24.28 |
|  |  | Shifting Tray | 6.63 | 4.01 | 1.34 | 16.57 |
|  |  | Inhibition Grid | -3.39 | 2.55 | -9.68 | 0.04 |
|  |  | Inhibition Cylinder | 4.75 | 3.15 | 0.73 | 12.67 |
|  |  | Inhibition Boxes | 3.21 | 2.62 | -0.42 | 9.62 |
|  | WM | WM Updating | 1.00 |  |  |  |
|  |  | WM Boxes | -1.61 | 7.16 | -16.91 | 13.13 |
|  |  | WM Grid | 2.21 | 7.16 | -12.93 | 17.04 |

Table S36. Model estimates for all models and their comparison against the 9-factor baseline model for the CFA with the prior adjusted to Gamma (1,2).

| **Model** | **WAIC** | **SE** | **WAIC difference** | **SE difference** | **p difference** |
| --- | --- | --- | --- | --- | --- |
| 1-factor | 3749.94 | 113.85 | -12.75 | 5.86 | .029 |
| MF2012 | 3753.94 | 113.86 | -10.75 | 5.58 | .054 |
| 3 factors | 3750.99 | 113.54 | -12.22 | 5.70 | .032 |
| 3 independent factors | 3763.30 | 113.65 | -6.07 | 4.39 | .167 |
| 2factors 1 | 3754.87 | 113.64 | -10.28 | 5.62 | .067 |
| 2factors 2 | 3759.97 | 113.55 | -7.73 | 4.71 | .101 |
| 2factors 3 | 3753.95 | 113.75 | -10.74 | 5.20 | .039 |

Table S37. Model comparisons between the 1-factor model and all competing models for the CFA with the prior adjusted to Gamma (1,2).

| **Model** | **WAIC difference** | **SE difference** | **p difference** |
| --- | --- | --- | --- |
| MF2012 | -2.003 | 0.860 | .020 |
| 3 factors | -0.525 | 1.951 | .788 |
| 3 independent factors | -6.684 | 3.865 | .084 |
| 2factors 1 | -2.469 | 2.533 | .330 |
| 2factors 2 | -5.018 | 4.209 | .233 |
| 2factors 3 | -2.005 | 3.952 | .612 |

Table S38. Proportion of variance in performance explained by the shared factor on each of the nine EF tasks for the CFA with the Gamma (1,2) prior (median and 95% credible interval).

| **Task** | **Proportion of variance explained (median)** | **95% credible interval** | |
| --- | --- | --- | --- |
| Inhibition Boxes | .034 | .001 | .135 |
| Inhibition Cylinder | .002 | .001 | .008 |
| Inhibition Grid | .036 | .001 | .130 |
| Shifting Boxes | .086 | .012 | .226 |
| Shifting Shelf | .425 | .178 | .777 |
| Shifting Tray | .172 | .052 | .363 |
| WM Boxes | .031 | .001 | .124 |
| WM Grid | .103 | .014 | .264 |
| WM Updating | .011 | .000 | .073 |

Table S39. Variance-covariance matrix for the 1-factor in run 1 for the CFA with the prior adjusted to Gamma (1,2).

| Task | Inhibition Boxes | Inhibition Cylinder | Inhibition Grid | Shifting Boxes | Shifting Shelf | Shifting Tray | WM Boxes | WM Grid | WM Updating |
| --- | --- | --- | --- | --- | --- | --- | --- | --- | --- |
| Inhibition Boxes | 1.035 |  |  |  |  |  |  |  |  |
| Inhibition Cylinder | 0.012 | 1.000 |  |  |  |  |  |  |  |
| Inhibition Grid | -0.053 | -0.012 | 1.032 |  |  |  |  |  |  |
| Shifting Boxes | 0.082 | 0.019 | -0.085 | 1.056 |  |  |  |  |  |
| Shifting Shelf | 0.179 | 0.042 | -0.185 | 0.289 | 1.186 |  |  |  |  |
| Shifting Tray | 0.117 | 0.027 | -0.120 | 0.188 | 0.410 | 1.098 |  |  |  |
| WM Boxes | 0.049 | 0.012 | -0.051 | 0.080 | 0.173 | 0.113 | 1.031 |  |  |
| WM Grid | 0.089 | 0.021 | -0.092 | 0.144 | 0.313 | 0.203 | 0.086 | 1.062 |  |
| WM Updating | 0.028 | 0.006 | -0.028 | 0.045 | 0.097 | 0.063 | 0.027 | 0.048 | 1.016 |

### **Chimpanzees**

Table S40. Correlations (and sample sizes) of the EF tasks for all chimpanzees (all variables mean centered by site).

|  | Inhibition Boxes | Inhibition Cylinders | Inhibition Grid | Shifting Boxes | Shifting Shelf | Shifting Tray | WM Boxes | WM Grid |
| --- | --- | --- | --- | --- | --- | --- | --- | --- |
| Inhibition Cylinders | 0.32* (51) |  |  |  |  |  |  |  |
| Inhibition Grid | 0.1 (52) | 0.23 (52) |  |  |  |  |  |  |
| Shifting Boxes (CD) | -0.19 (53) | 0 (51) | -0.06 (52) |  |  |  |  |  |
| Shifting Shelf | -0.22 (50) | -0.07 (48) | -0.06 (49) | 0.07 (50) |  |  |  |  |
| Shifting Tray | -0.25 (52) | 0.01 (50) | -0.07 (51) | 0.3 * (52) | 0.08 (50) |  |  |  |
| WM Boxes | -0.14 (53) | -0.09 (51) | 0 (52) | 0.19 (53) | 0.22 (50) | 0.31 * (52) |  |  |
| WM Grid | 0.21 (53) | -0.25 (51) | 0.22 (52) | 0 (53) | 0.13 (50) | -0.11 (52) | 0.24 (53) |  |
| WM Updating | -0.2 (53) | -0.03 (51) | 0.17 (52) | 0.04 (53) | 0.2 (50) | -0.11 (52) | 0.11 (53) | 0.05 (53) |

Notes. Significant positive correlations between tasks are highlighted, * = p < .05.

#### **Domain-specific models**

Table S41. Model estimates of the chimpanzee data for all 1-factor domain-specific models and their comparison against their respective 3-factor baseline models.

| Models | WAIC | SE | WAIC difference | SE difference | p difference |
| --- | --- | --- | --- | --- | --- |
| WM model | 461.32 | 20.31 | -0.32 | 0.43 | 0.46 |
| Inhibition model | 458.18 | 19.8 | -0.81 | 1.55 | 0.6 |
| Shifting model | 448.81 | 17.8 | -0.2 | 0.46 | 0.66 |

Table S42. Factor loadings of the chimpanzee data for the one-factor domain-specific models.

| **Model** | **Task** | **Estimate** | **Post.sd** | **95% credible interval** | |
| --- | --- | --- | --- | --- | --- |
| WM model | WM Updating | 1.00 |  |  |  |
|  | WM Boxes | 1.88 | 7.22 | -14.45 | 16.59 |
|  | WM Grid | 0.85 | 7.43 | -16.00 | 16.53 |
| Inhibition model | Inhibition Cylinder | 1.00 |  |  |  |
|  | Inhibition Grid | 3.22 | 6.24 | -11.99 | 16.18 |
|  | Inhibition Boxes | 1.71 | 5.82 | -12.55 | 14.51 |
| Shifting model | Shifting Boxes | 1.00 |  |  |  |
|  | Shifting Shelf | 0.83 | 6.57 | -14.50 | 15.61 |
|  | Shifting Tray | 2.21 | 6.67 | -13.50 | 15.86 |

#### **CFA**

Table S43. Model estimates of the chimpanzee data for all models and their comparison against the 9-factor baseline model.

| Models | WAIC | SE | WAIC difference | SE difference | p difference |
| --- | --- | --- | --- | --- | --- |
| 1 factor | 1369.83 | 45.95 | -0.4 | 0.67 | 0.55 |
| MF 2012 | 1371.3 | 46.18 | -1.14 | 1.01 | 0.26 |
| 3 factors | 1370.34 | 45.75 | -0.66 | 1.28 | 0.61 |
| 3 independent factors | 1368.89 | 45.64 | -0.06 | 1.76 | 0.97 |
| 2 factors 1 | 1368.52 | 45.5 | -0.25 | 1.63 | 0.88 |
| 2 factors 2 | 1370.55 | 46.16 | -0.77 | 0.92 | 0.4 |
| 2 factors 3 | 1371.06 | 45.94 | -1.02 | 0.7 | 0.15 |

Table S44. Factor loadings of the chimpanzee data for all investigated models.

| **Model** | **Latent Factor** | **Task** | **Estimate** | **Post.sd** | **95% credible interval** | |
| --- | --- | --- | --- | --- | --- | --- |
| 1 factor | Common | Inhibition Boxes | 1.00 |  |  |  |
|  |  | Inhibition Cylinder | 0.77 | 8.48 | -16.59 | 17.31 |
|  |  | Inhibition Grid | 0.22 | 8.28 | -16.57 | 17.09 |
|  |  | Shifting Boxes | -1.15 | 9.36 | -18.98 | 17.66 |
|  |  | Shifting Shelf | -1.06 | 9.14 | -18.50 | 17.40 |
|  |  | Shifting Tray | -1.33 | 10.01 | -20.32 | 18.42 |
|  |  | WM Boxes | -1.51 | 11.14 | -21.44 | 20.43 |
|  |  | WM Grid | -0.60 | 9.63 | -20.69 | 18.23 |
|  |  | WM Updating | -0.51 | 8.36 | -17.42 | 16.32 |
| MF 2012 | WM | WM Boxes | 1.00 |  |  |  |
|  |  | WM Grid | 1.50 | 7.23 | -14.89 | 16.83 |
|  |  | WM Updating | 0.60 | 7.17 | -15.44 | 15.98 |
|  | Shifting | Shifting Boxes | 1.00 |  |  |  |
|  |  | Shifting Shelf | 0.37 | 6.99 | -15.46 | 15.51 |
|  |  | Shifting Tray | 1.91 | 7.15 | -14.49 | 16.65 |
|  | Common | Inhibition Boxes | 1.00 |  |  |  |
|  |  | Inhibition Cylinder | 0.72 | 8.48 | -16.57 | 17.62 |
|  |  | Inhibition Grid | 0.30 | 8.28 | -16.77 | 17.16 |
|  |  | Shifting Shelf | -1.11 | 9.25 | -18.93 | 17.36 |
|  |  | Shifting Boxes | -0.99 | 9.13 | -18.47 | 17.65 |
|  |  | Shifting Tray | -1.45 | 10.09 | -20.40 | 18.56 |
|  |  | WM Boxes | -1.69 | 10.94 | -22.02 | 19.61 |
|  |  | WM Grid | -0.07 | 9.35 | -18.25 | 19.21 |
|  |  | WM Updating | -0.37 | 8.60 | -17.57 | 17.46 |
| 3 Factors | WM | WM Boxes | 1.00 |  |  |  |
|  |  | WM Grid | 1.49 | 7.33 | -15.06 | 16.38 |
|  |  | WM Updating | 0.54 | 6.63 | -13.90 | 14.95 |
|  | Shifting | Shifting Boxes | 1.00 |  |  |  |
|  |  | Shifting Shelf | 0.78 | 6.94 | -14.68 | 15.65 |
|  |  | Shifting Tray | 1.45 | 6.73 | -13.66 | 15.27 |
|  | Inhibition | Inhibition Boxes | 1.00 |  |  |  |
|  |  | Inhibition Cylinder | 2.47 | 6.04 | -12.30 | 15.21 |
|  |  | Inhibition Grid | 1.43 | 5.78 | -12.64 | 14.38 |
| 3 independent factors | WM | WM Boxes | 1.00 |  |  |  |
|  |  | WM Grid | 1.75 | 7.04 | -14.57 | 16.20 |
|  |  | WM Updating | 0.77 | 6.96 | -15.07 | 16.01 |
|  | Shifting | Shifting Boxes | 1.00 |  |  |  |
|  |  | Shifting Shelf | 0.64 | 6.39 | -14.09 | 14.90 |
|  |  | Shifting Tray | 2.32 | 6.74 | -13.53 | 16.37 |
|  | Inhibition | Inhibition Boxes | 1.00 |  |  |  |
|  |  | Inhibition Cylinder | 3.20 | 5.81 | -10.82 | 15.40 |
|  |  | Inhibition Grid | 1.69 | 5.78 | -12.62 | 14.48 |
| 2 factors: WM+Shifting, Inhibition | WM + Shifting | WM Boxes | 1.00 |  |  |  |
|  |  | WM Grid | 0.28 | 8.03 | -16.25 | 17.17 |
|  |  | WM Updating | 0.17 | 8.26 | -16.79 | 17.32 |
|  |  | Shifting Boxes | 1.25 | 9.22 | -17.56 | 18.93 |
|  |  | Shifting Shelf | 0.74 | 8.47 | -16.96 | 17.74 |
|  |  | Shifting Tray | 1.32 | 9.81 | -18.48 | 20.33 |
|  | Inhibition | Inhibition Boxes | 1.00 |  |  |  |
|  |  | Inhibition Cylinder | 3.19 | 6.12 | -11.78 | 15.94 |
|  |  | Inhibition Grid | 1.65 | 5.83 | -12.63 | 14.45 |
| 2 factors: WM + Inhibition, Shifting | WM + Inhibition | Inhibition Boxes | 1.00 |  |  |  |
|  |  | Inhibition Cylinder | 1.08 | 8.87 | -16.30 | 19.34 |
|  |  | Inhibition Grid | 0.49 | 8.62 | -18.30 | 17.57 |
|  |  | WM Boxes | -0.35 | 8.56 | -18.06 | 16.65 |
|  |  | WM Grid | 0.34 | 10.55 | -19.77 | 21.07 |
|  |  | WM Updating | -0.34 | 7.42 | -16.04 | 14.89 |
|  | Shifting | Shifting Boxes | 1.00 |  |  |  |
|  |  | Shifting Shelf | 0.83 | 6.37 | -13.83 | 15.06 |
|  |  | Shifting Tray | 2.39 | 6.68 | -13.31 | 16.19 |
| 2 factors: Shifting + Inhibition, WM | Shifting + Inhibition | Inhibition Boxes | 1.00 |  |  |  |
|  |  | Inhibition Cylinder | 0.93 | 8.38 | -16.26 | 18.04 |
|  |  | Inhibition Grid | 0.97 | 8.60 | -16.94 | 18.29 |
|  |  | Shifting Boxes | -1.38 | 9.19 | -19.09 | 17.33 |
|  |  | Shifting Shelf | -0.83 | 7.74 | -16.54 | 15.58 |
|  |  | Shifting Tray | -1.51 | 9.37 | -19.44 | 17.70 |
|  | WM | WM Boxes | 1.00 |  |  |  |
|  |  | WM Grid | 1.73 | 7.23 | -14.64 | 16.62 |
|  |  | WM Updating | 0.75 | 7.05 | -14.86 | 15.93 |

## Exploratory Factor Analysis

### **Children**

Table S45. Correlations for the EF tasks for the 95 children who had valid data on all nine tasks (all variables mean centered by site).

|  | Inhibition Boxes | Inhibition Cylinder | Inhibition Grid | Shifting Boxes | Shifting Shelf | Shifting Tray | WM Boxes | WM Grid | WM Updating |
| --- | --- | --- | --- | --- | --- | --- | --- | --- | --- |
| Inhibition Boxes |  |  |  |  |  |  |  |  |  |
| Inhibition Cylinder | 0.19 |  |  |  |  |  |  |  |  |
| Inhibition Grid | 0.16 | 0.00 |  |  |  |  |  |  |  |
| Shifting Boxes | 0.09 | 0.23* | -0.13 |  |  |  |  |  |  |
| Shifting Shelf | 0.16 | 0.28** | -0.06 | 0.28** |  |  |  |  |  |
| Shifting Tray | 0.04 | 0.16 | -0.20* | 0.18 | 0.36*** |  |  |  |  |
| WM Boxes | 0.05 | 0.11 | 0.04 | 0.10 | 0.08 | 0.04 |  |  |  |
| WM Grid | 0.05 | 0.14 | 0.08 | 0.10 | 0.28** | 0.13 | 0.04 |  |  |
| WM Updating | 0.01 | 0.00 | -0.02 | 0.08 | -0.03 | -0.06 | -0.24* | 0.18 |  |

Notes. Significant correlations between tasks are highlighted, * = p < .05; ** = p < .01; *** = p < .001.


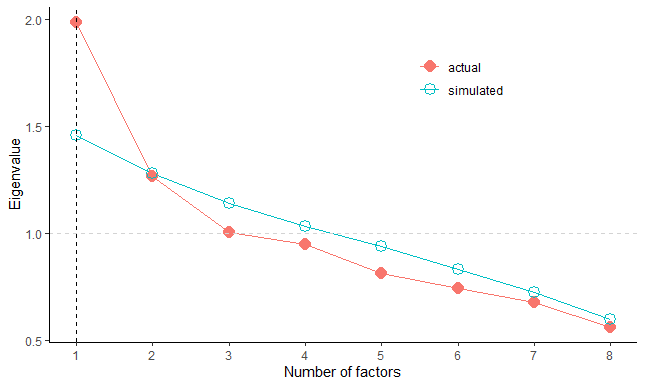


Fig. S31. Scree plot showing the eigenvalues of the actual data and the results of the parallel analysis (simulated values) for the child sample. The parallel analysis is based on using maximum likelihood factoring and 100 iterations. The parallel analysis suggested retaining one factor.


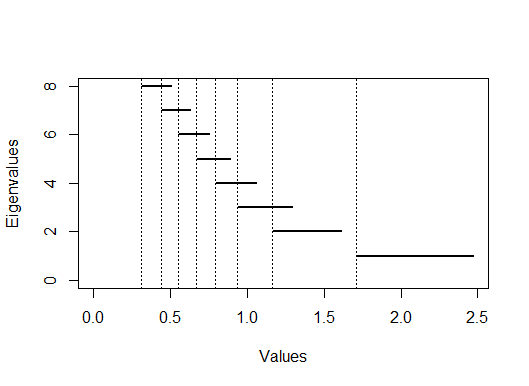


Fig. S32. 90% Confidence intervals of the eigenvalues of the actual data for the child sample (factor 1: [1.79; 2.48]; factor 2: [1.15; 1.67]; factor 3: [0.94; 1.24]).

Table S46. Factor loadings of the EFA (varimax rotation) of the children data retaining either 1 factor or 2 factors.

|  | **1-factor model** | **2-factor model** | |
| --- | --- | --- | --- |
| **Task** | **Factor 1** | **Factor 1** | **Factor 2** |
| Inhibition Boxes | 0.21 | 0.23 | -0.25 |
| Inhibition Cylinders | 0.41 | 0.42 | 0.06 |
| Inhibition Grid | -0.10 | -0.10 | 0.84 |
| Shifting Boxes | 0.40 | 0.40 | -0.10 |
| Shifting Shelf | 0.74 | 0.74 | 0.01 |
| Shifting Tray | 0.47 | 0.47 | -0.19 |
| WM Boxes | 0.13 | 0.14 | 0.06 |
| WM Grid | 0.34 | 0.34 | 0.13 |

### **Chimpanzees**


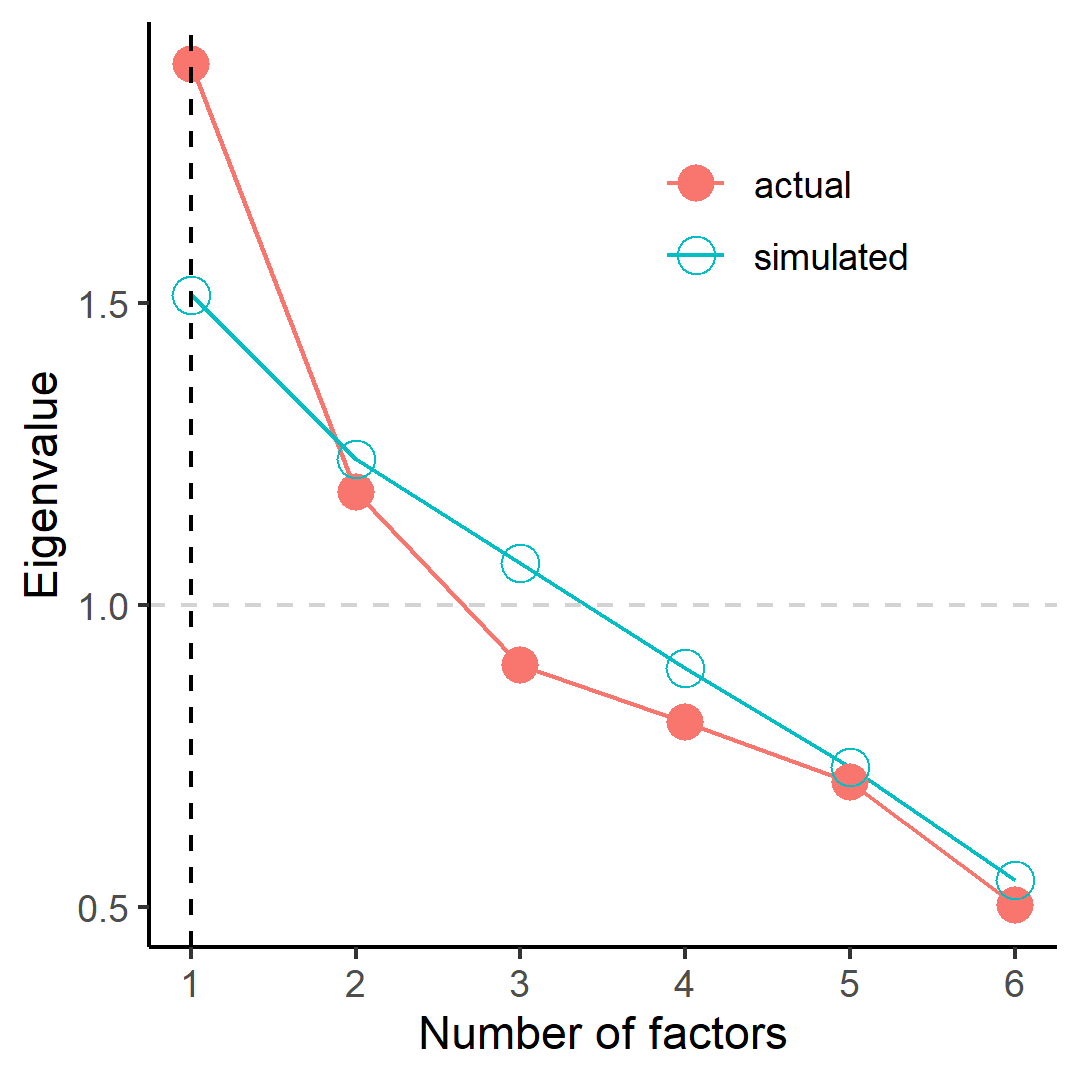


Fig. S33. Scree plot showing the eigenvalues of the actual data and the results of the parallel analysis (simulated values). The parallel analysis is based on using maximum likelihood factoring and 100 iterations. The parallel analysis suggests retaining one factor.


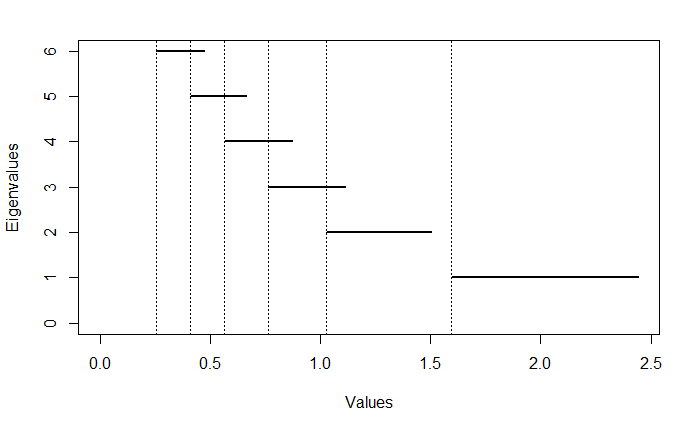


Fig. S34. 90% Confidence intervals of the eigenvalues of the actual data for the chimpanzee sample (factor 1: [1.60; 2.42]; factor 2: [1.02; 1.50]; factor 3: [0.75; 1.12]).

Table S47. Factor loadings of the exploratory factor analysis (varimax rotation) of the chimp data retaining either 1 factor or 2 factors.

|  | **1-factor model** | **2-factor model** | |
| --- | --- | --- | --- |
| **Task** | **Factor 1** | **Factor 1** | **Factor 2** |
| Inhibition Boxes | -0.41 | 0.97 | -0.25 |
| Inhibition Cylinders | -0.15 | 0.34 | -0.01 |
| Shifting Boxes | 0.41 | -0.08 | 0.67 |
| Shifting Shelf | 0.32 | -0.08 | 0.39 |
| Shifting Tray | 0.62 | -0.15 | 0.26 |
| WM Boxes | 0.55 | 0.01 | 0.57 |

## Supplementary references

1. Mayer, C. The evolutionary origins of executive functions : behavioural control in humans and chimpanzees. (University of St Andrews, 2015).

2. Völter, C. J., Mundry, R., Call, J. & Seed, A. M. Chimpanzees flexibly update working memory contents and show susceptibility to distraction in the self-ordered search task. *Proc. R. Soc. B* (2019) doi:10.1098/rspb.2019.0715.

3. Gioia, G. A., Espy, K. A. & Isquith, P. K. *BRIEF-P: Behavior rating inventory of executive function–preschool version*. (Psychological Assessment Resources (PAR), 2003).

4. Merkle, E. C. & Rosseel, Y. blavaan: Bayesian structural equation models via parameter expansion. *Journal of Statistical Software* **85**, 1–30 (2018).
